# Insect Pollinated Crops, Insect Pollinators and US Agriculture: Trend Analysis of Aggregate Data for the Period 1992–2009

**DOI:** 10.1371/journal.pone.0037235

**Published:** 2012-05-22

**Authors:** Nicholas W. Calderone

**Affiliations:** Department of Entomology, Cornell University, Ithaca, New York, United States of America; Ghent University, Belgium

## Abstract

In the US, the cultivated area (hectares) and production (tonnes) of crops that require or benefit from insect pollination (directly dependent crops: apples, almonds, blueberries, cucurbits, etc.) increased from 1992, the first year in this study, through 1999 and continued near those levels through 2009; aggregate yield (tonnes/hectare) remained unchanged. The value of directly dependent crops attributed to all insect pollination (2009 USD) decreased from $14.29 billion in 1996, the first year for value data in this study, to $10.69 billion in 2001, but increased thereafter, reaching $15.12 billion by 2009. The values attributed to honey bees and non-*Apis* pollinators followed similar patterns, reaching $11.68 billion and $3.44 billion, respectively, by 2009. The cultivated area of crops grown from seeds resulting from insect pollination (indirectly dependent crops: legume hays, carrots, onions, etc.) was stable from 1992 through 1999, but has since declined. Production of those crops also declined, albeit not as rapidly as the decline in cultivated area; this asymmetry was due to increases in aggregate yield. The value of indirectly dependent crops attributed to insect pollination declined from $15.45 billion in 1996 to $12.00 billion in 2004, but has since trended upward. The value of indirectly dependent crops attributed to honey bees and non-*Apis* pollinators, exclusive of alfalfa leafcutter bees, has declined since 1996 to $5.39 billion and $1.15 billion, respectively in 2009. The value of alfalfa hay attributed to alfalfa leafcutter bees ranged between $4.99 and $7.04 billion. Trend analysis demonstrates that US producers have a continued and significant need for insect pollinators and that a diminution in managed or wild pollinator populations could seriously threaten the continued production of insect pollinated crops and crops grown from seeds resulting from insect pollination.

## Introduction

Flowering plants (Angiosperms) play critical roles in many natural and agricultural ecosystems, providing food, fiber and shelter for wildlife and humankind alike [1]. In humans, high levels of fruit and vegetable consumption are associated with decreased risk of chronic disease [2]–[5]. Additionally, there is growing interest in the use of plants as fuel sources [6]–[11]. Pollination is an essential step in the reproductive process of the world's nearly 300,000 species of flowering plants because it is usually required for the production of seeds [1], [12]–[17]. Pollination is the transfer of pollen, bearing the male gamete, from the anther of a flower to the stigma of a flower. After landing on a receptive stigma, a pollen grain germinates and a pollen tube develops, growing through the supporting style to the ovary. Genetic material in the pollen grain travels through the pollen tube to the ovary where it unites with an egg, the female gamete, in a process called fertilization. The fertilized egg develops into a seed, and that process is often accompanied by the development of fruit from surrounding tissue [18]. Depending on the species, from one to several hundred eggs must be fertilized to ensure a high quality fruit because each egg requires a separate pollen grain for fertilization. Plants with incompletely pollinated flowers have fewer seeds and reduced fitness, and they produce inferior fruit with reduced market value [19], [20].

Pollination can result from the action of abiotic forces such as wind and water, but 80% of the Angiosperms rely on animals, including bats, flies, butterflies, beetles and other insects [1]. The majority of pollinators are insects, and the majority of those are bees (*Anthophila*) [13], of which there are approximately 17,000 described species and as many as 30,000 species worldwide [1], [21]. With rare exception, bees collect pollen and nectar from flowers for food, transferring pollen in the process. North America is home to nearly 4,500 species of bees [21]. Most are solitary, but there are 49 known species of the primitively eusocial bumble bee in the US, 41 of which are also found in Canada; an additional 11 species are found in Mexico. The highly eusocial western honey bee, *Apis mellifera*, was introduced to North America from Europe and Africa beginning in 1622 [22], [23]. It is the only species of honey bee in North America.

Recent events affecting the health of honey bees and other insect pollinators [1], both in the US and abroad, have renewed interest in the pollination services they provide in both natural and agricultural ecosystems [14], [24]–[28]. This concern is driven, in part, by data showing that the global cultivation of pollinator-dependent crops is increasing [29]–[31] while certain populations of native and managed pollinator species are declining or at risk [1], [32], [33]. Threats to native pollinator populations include agricultural intensification, habitat alteration and fragmentation, exotic pathogens, nutritional stress, pesticides and the loss of genetic variability, the latter being especially significant for the haplodiploid bees [25], [34]–[47]; however, the impact of anthropogenic disturbances on bee abundance and species richness has not been well documented on a global level [48]. Additionally, the nature of the impact of declining pollinator populations is controversial. Crops that provide the majority of global calories do not require pollination [49], [50] while those that provide other nutrients do require pollination [51].

Globally, the population of managed honey bees is increasing, albeit not at a rate that matches the global growth in the production of pollinator-dependent crops [30]; however, that growth is not shared by managed honey bees in the US [52]. Although the US honey bee population has a history of occasional precipitous, short-term losses [53], there has been a gradual, sustained decline since the peak of 5.9 million colonies in 1947 [52]. The number of managed colonies in the US reached a low of 2.3 million in 2008, although there were increases in 2009 and 2010 (methods for estimating colony numbers are discussed elsewhere [54]).

**Figure 1:**
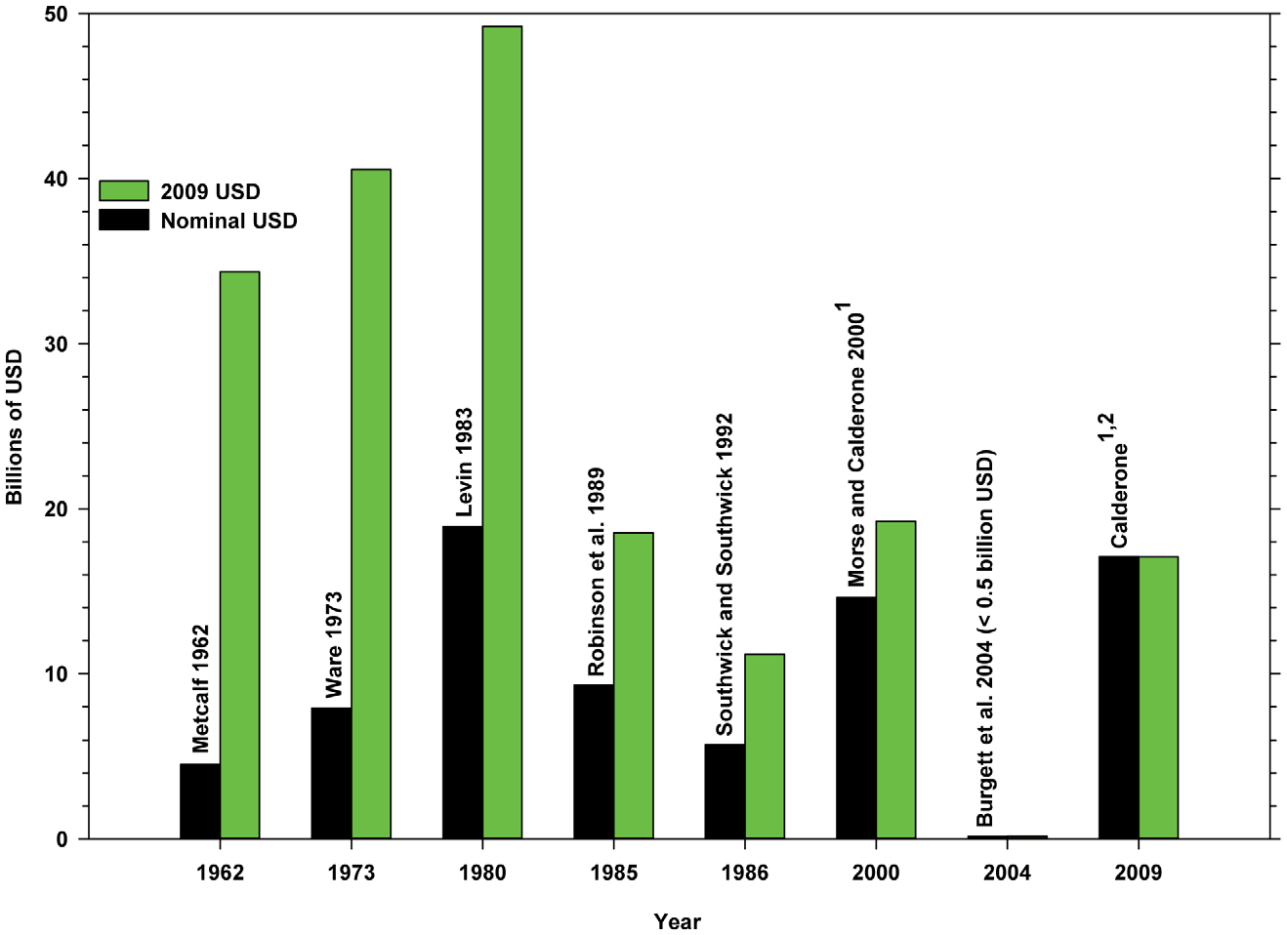
Historical estimates of the value of honey bees to US agriculture. ^1^Includes both directly dependent crops (apples, almonds, cherries, oranges, squash, vegetable and legume seeds, etc.) and indirectly dependent crops (field crops and vegetables); ^2^present study.

Because honey bees and other insects play a pivotal role in many agricultural cropping systems, several estimates of the value they contribute to US agriculture have been published (Fig. 1; billion = B): $4.5 B in 1957 [55] (Metcalf), $7.9 B in 1972 [56] (Ware), $18.9 B in 1980 [57] (Levin), $1.6–5.7 B in 1986 [58] (Southwick and Southwick), $9.3 B in 1985 [59], [60] (Robinson, Nowogrodzki, Morse), $14.6 B in 1996–1998 [61] (Morse and Calderone) and $150 million in 2004 [62] (Burgett, Rucker and Thurman). Inflation adjusted equivalents (2009 USD) are $34.36 B (Metcalf), $40.55 B (Ware), $49.21 B (Levin), $3.13 B–$11.16 B (Southwick and Southwick), $18.54 B (Robinson, Nowogrodzki, Morse), $19.22 B (Morse and Calderone) and $170.36 million (Burgett, Rucker and Thurman). The annual value of native pollinators for the period 2001–2003 is estimated at $3.07 B (∼$3.66 B 2009 USD) [63] (Losey and Vaughan).

The variation in the above estimates can be attributed to the different approaches taken by the various authors. Metcalf [55] reported the total gross value of a group of 30 insect pollinated crops deemed to depend ‘almost exclusively’ upon insects for production but did not differentiate among the contributions of honey bees, non-*Apis* bees and other insects. Levin [57] included the total gross value of crops that require or benefit directly from bee pollination (directly dependent crops, hereafter DD crops: e.g. apples, almonds, cherries, oranges, squash, vegetable and legume seeds, etc.), the total value of crops that do not require pollination but that are grown from seeds that result from pollination (indirectly dependent crops, hereafter ID crops: including field crops (legume hay, sugar beets, etc.) and vegetables (asparagus, broccoli, carrots, onions, etc.)) and 10% of the value of beef and dairy production resulting from the consumption of legume hay by cattle. Robinson, Nowogrodzki and Morse [59], [60] and Morse and Calderone [61] present combined values for DD and ID crops but reduce the total gross values to reflect the estimated proportion due to honey bees; they do not include commodities further along the food chain. Southwick and Southwick [58] base their estimate of value on an analysis of supply and demand functions, defining value as “the surplus realized by consumers of these crops that would be lost if honey bees were depleted.” Burgett, Rucker and Thurman [62] count only the value of pollination fees paid to beekeepers.

Several studies document the increasing cultivation and production of animal-pollinated crops on a global level [29]–[31], [64]; however, studies specific to the US are lacking. Previous studies of insect pollination and US agriculture focus primarily on honey bees, a single year, or both. While those studies provide snapshots of the relationships between insect pollinators and US agriculture, they do not reveal trends in those relationships. Here, I present a comprehensive analysis of trends in aggregate production, cultivated area and farmgate value for 58 pollinator-dependent crops over an 18 year period from 1992–2009. I distinguish between, and report separately, statistics for DD and ID crops; and I present values for both honey bees and non-*Apis* pollinators. The primary goal in modeling these trends is to quantify the degree of dependence of US agriculture on insect pollinators and to determine if that dependence is declining, stable or increasing. To illuminate the contributions of individual crops, I present three, single-year snapshots (2002, 2007 and 2010). Additionally, I discuss dependency coefficients and valuation methods, two issues relevant to efforts to quantify the contributions of insect pollinators to agriculture. Lastly, I examine the question of a pollinator shortage in the US.

## Materials and Methods

### US population and farm data

#### General methods and sources of US population and farm data

Data on land in farms and the value of cropland were obtained from USDA National Agricultural Statistics Service (NASS: Farms and Land in Farms - Final Estimates 1993–97, 1998–2002, 2003–2007; Farms and Land in Farms 02-26-1999, 02-12-2010; Agricultural Land Values and Cash Rents – Final Estimates 1993–2003, 2004–2008; Land Values and Cash Rents 2010 Summary; and the 1997, 2002 and 2007 NASS Census of Agriculture reports) [65]–[87]. Acres were converted to hectares. Nominal values in USD were converted to 2009 USD (Table 1) using the CPI Index from the US Department of Labor, Bureau of Labor Statistics [88].

**Table 1:** General farm and US population data.

Year	US Population 1	THIF 1 ^,^ 2	Cropland value (nominal USD per hectare)	Cropland value (2009 USD per hectare)
**1992**	256.51	395.99	na	na
**1993**	259.92	392.08	na	na
**1994**	263.13	390.90	na	na
**1995**	266.28	389.52	na	na
**1996**	269.39	387.96	na	na
**1997**	272.65	386.88	3,138.24	4,194.81
**1998**	275.85	385.29	3,311.21	4,358.15
**1999**	279.04	383.83	3,484.19	4,486.72
**2000**	282.17	382.46	3,607.74	4,494.72
**2001**	285.08	381.24	3,731.29	4,520.05
**2002**	287.80	380.53	3,928.98	4,685.44
**2003**	290.33	379.09	4,101.95	4,782.72
**2004**	293.05	377.27	4,373.77	4,967.36
**2005**	295.75	375.52	5,090.37	5,591.77
**2006**	298.59	374.65	5,683.42	6,048.13
**2007**	301.58	372.90	6,251.77	6,468.70
**2008**	304.37	372.27	6,820.11	6,795.84
**2009**	307.01	372.23	6,597.71	6,597.71

1 millions;

2 hectares; THIF = total hectares in farms; na = not available.

#### Trend analysis for US population and general farm data

I examined trends for the following variables for general farm and population data: 1) US population; 2) total hectares in farms; and 3) value of US cropland (2009 USD).

### Crop data

#### General methods and sources of crop data

I obtained data for 58 pollinator-dependent crops from 1992 to 2009. Data for production, units of production, cultivated acres (planted acres when available, otherwise harvested/bearing acres) and the value of production were obtained from NASS (Final Estimates for 1986–2007, Annual Reports for 2008 and 2009, and the 2002 and 2007 Census of Agriculture (COA) reports) [89]–[108]. Production data for each crop in crop-specific units (e.g. cwt, boxes, etc.) were converted to common units (tonnes); cultivated acres were converted to hectares. Aggregate yield for each year was estimated by dividing total aggregate production in tonnes summed over all crops by the corresponding total aggregate number of cultivated hectares. Nominal values in USD were converted to 2009 USD.

For each year, the number of hectares of DD crops expressed as a percentage of total hectares in farms (Table 2) was calculated by dividing the annual aggregate number of hectares of DD crops by the corresponding total number of hectares in farms. For each year, the total number of hectares of DD crops expressed as hectares per person was calculated by dividing the aggregate number of hectares of DD crops by the corresponding estimate for the US population (Table 2). Corresponding estimates for production were calculated using the same method (Table 3). Equivalent estimates were calculated for ID crops (Table 2 and Table 3).

**Table 2:** Hectares of Directly and Indirectly Dependent Crops.

Year	HDD 1 ^,^ 2	HDD as % THIF 4	HDD crops per person	HID as % THIF 4	HID 1 ^,^ 3	HID crops per person	US Population 1
**1992**	26.65	6.73	0.1039	3.80	15.03	0.0586	256.51
**1993**	26.52	6.76	0.1020	4.07	15.96	0.0614	259.92
**1994**	28.38	7.26	0.1079	4.09	15.98	0.0607	263.13
**1995**	28.68	7.36	0.1077	4.41	17.16	0.0645	266.28
**1996**	28.99	7.47	0.1076	4.07	15.79	0.0586	269.39
**1997**	31.60	8.17	0.1159	4.08	15.77	0.0578	272.65
**1998**	32.63	8.47	0.1183	3.81	14.69	0.0532	275.85
**1999**	33.42	8.71	0.1198	4.18	16.03	0.0574	279.04
**2000**	33.26	8.70	0.1179	4.07	15.57	0.0552	282.17
**2001**	33.45	8.77	0.1173	4.20	16.02	0.0562	285.08
**2002**	32.97	8.67	0.1146	3.96	15.07	0.0523	287.80
**2003**	32.89	8.68	0.1133	3.99	15.13	0.0521	290.33
**2004**	33.21	8.80	0.1133	3.92	14.80	0.0505	293.05
**2005**	32.66	8.70	0.1104	4.09	15.34	0.0519	295.75
**2006**	33.44	8.92	0.1120	3.85	14.44	0.0483	298.59
**2007**	29.34	7.87	0.0973	3.62	13.50	0.0448	301.58
**2008**	33.81	9.08	0.1111	3.28	12.21	0.0401	304.37
**2009**	34.11	9.16	0.1111	3.32	12.35	0.0402	307.01

1 millions;

2 HDD = hectares directly dependent crops;

3 HID = hectares indirectly dependent crops;

4 THIF = total hectares in farms.

**Table 3:** Production of Directly and Indirectly Dependent Crops.

Year	Tonnes DD crops 1	Tonnes DD crops per person	Tonnes ID crops 1	Tonnes ID crops per person	US Population 1
**1992**	98.9255	0.4251	107.6731	0.4627	256.51
**1993**	92.0909	0.3906	106.3243	0.4509	259.92
**1994**	112.7269	0.4722	113.8044	0.4768	263.13
**1995**	102.1451	0.4228	112.4924	0.4657	266.28
**1996**	107.7844	0.4410	107.0707	0.4381	269.39
**1997**	119.8173	0.4844	109.8278	0.4440	272.65
**1998**	119.9575	0.4793	113.6954	0.4543	275.85
**1999**	114.9755	0.4542	117.9397	0.4659	279.04
**2000**	121.9736	0.4765	114.4079	0.4469	282.17
**2001**	124.3230	0.4807	107.5862	0.4160	285.08
**2002**	118.8422	0.4552	101.8749	0.3902	287.80
**2003**	110.3651	0.4190	107.9457	0.4098	290.33
**2004**	130.5823	0.4912	108.1939	0.4070	293.05
**2005**	127.0099	0.4734	105.7034	0.3940	295.75
**2006**	127.2814	0.4699	106.4888	0.3931	298.59
**2007**	112.2107	0.4101	103.6566	0.3789	301.58
**2008**	121.8626	0.4413	97.3146	0.3524	304.37
**2009**	130.3399	0.4680	100.7376	0.3617	307.01

1 millions; DD = directly dependent crops; ID = indirectly dependent crops.

#### Partitioning value data

Partitioning value among honey bees and non-*Apis* pollinators was based on published coefficients of dependency [59], [60]. The proportion attributed to non-*Apis* pollinators was calculated as the difference between the portion of total crop value attributed to all insect pollinators and the portion attributed to honey bees [63]. In the case of ID crops, the assignment was based on the dependency coefficients for the production of the seeds used to produce those crops [59], [60]. For alfalfa hay, I generated a preliminary revision of the estimated proportions of value due to honey bees, leafcutter bees and other insect pollinators based on a review of production data for alfalfa seed (see Text S1).

### Trend analysis for annual US crop and colony data

I examined trends for the following variables aggregated over all crops on an annual basis: 1–2) total number of cultivated hectares for both DD crops and ID crops; 3–4) total number of cultivated hectares for both crop groups as a percentage of total hectares in farms; 5–6) total production in tonnes for both groups; 7–8) aggregate yield for both groups; 9–10) number of cultivated hectares per person for both groups; 11–12) total production in tonnes per person for both groups; 13–14) total value (2009 USD) of production for both groups; 15–21) portions of total value for both groups attributed to insect pollination, honey bees, alfalfa leafcutter bees and other insects.

### General analysis

#### Trends

Data were analyzed using regression analysis (PROC AUTOREG [109] with corrections for serial autocorrelation and/or heteroscedacity of variances where required to satisfy the assumptions of the analysis) with year as the independent variable. Trend analysis was limited to the period from 1992 through 2009 when there were no changes in the actual crops considered. Analysis of crop values was further limited to the period from 1996 to 2009 due to the inability to model data over the entire period from 1992 to 2009 (data for 1992–1995 are provided for informational purposes). Separate analyses were performed for DD and ID crops.

#### Data for individual crops

I report data for individual crops for the years 2002 and 2007 to illuminate the contributions of individual crops. Those years were selected because they are the most recent for which NASS Final Estimates and COA data were available [86], [87]. Using COA data allowed for the inclusion of data for crops not available on an annual basis (alfalfa and non-alfalfa legume seed production, pumpkins and squash) and makes totals for most variables slightly higher than corresponding values presented in the trend analyses for those years. Data for individual crops for 2010 [107], [108], [110]–[112] are also presented.

### Decline in the number of honey bee colonies and the pollinator shortage

The decline in the number of honey bee colonies [113]–[119], the number of colonies required to meet current recommendations (colonies per hectare) and their relationship to the adequacy of pollination services are analyzed.

### Other issues and underestimates

#### Vegetable seeds

Data for vegetable seeds are no longer collected by NASS and are not included in any current estimates. Previous estimates [59], [60] attribute 100% of vegetable seed production to insect pollination, with 90% of that due to honey bee pollination and 10% to other insects.Morse and Calderone [61] estimated that vegetable seed was worth an average of $61 million between 1996 and 1998.This could translate into an underestimate of $81.19 million (2009 USD) for DD crops for 2009, assuming no change in production.

#### Cotton lint

Cotton lint is produced from seed that requires insect pollination, making it a crop that benefits indirectly from pollination. However, lint production also benefits directly from having honey bees and other pollinators present during bloom [120], [121]. Therefore, value data are included for both direct and indirect contributions; however, to avoid duplication of data for production and cultivated hectares, those metrics are reported only as an indirect crop.

#### Tomatoes

Tomatoes are not included in the present study; however, fresh and processed tomatoes were valued at approximately $2.5 billion in 2009 [122] (2009 USD) with some undetermined proportion due to non-*Apis* insect pollinators [123].

#### Bumblebees

Bumblebees are a major pollinator of many greenhouse crops, including tomatoes [124], [125], peppers [126] and some berries [127]–[129]. They are also highly efficient pollinators of many field crops, including blueberries and cranberries (*Vaccinium* spp.) [130], [131]. Bumblebees are available commercially, typically as nests of 150 or 300 workers or as ‘quads’ with 600–1,200 bees; however, national data on the economic contributions of wild and managed bumblebees are not available. This results in an underestimate of the value of insect pollination and the value of non-*Apis* pollinators in particular.

## Results

### Results of Trend Analysis for US population and general farm data

**Figure 2:**
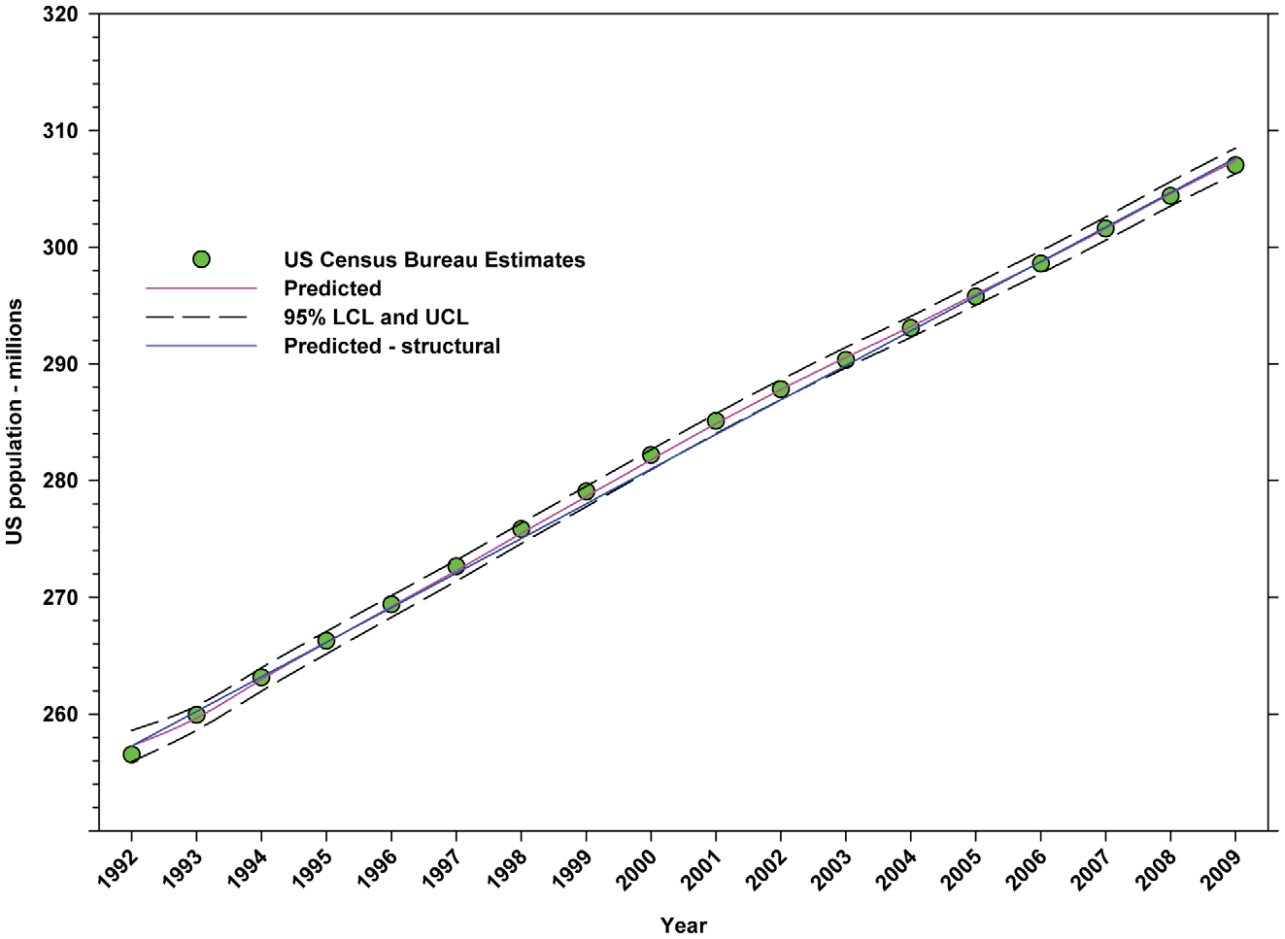
Estimates for the US population. Predicted values (pink) include adjustments for serial autocorrelation. Predicted – structural values (blue) are based solely on the structural elements of the model.

**Figure 3:**
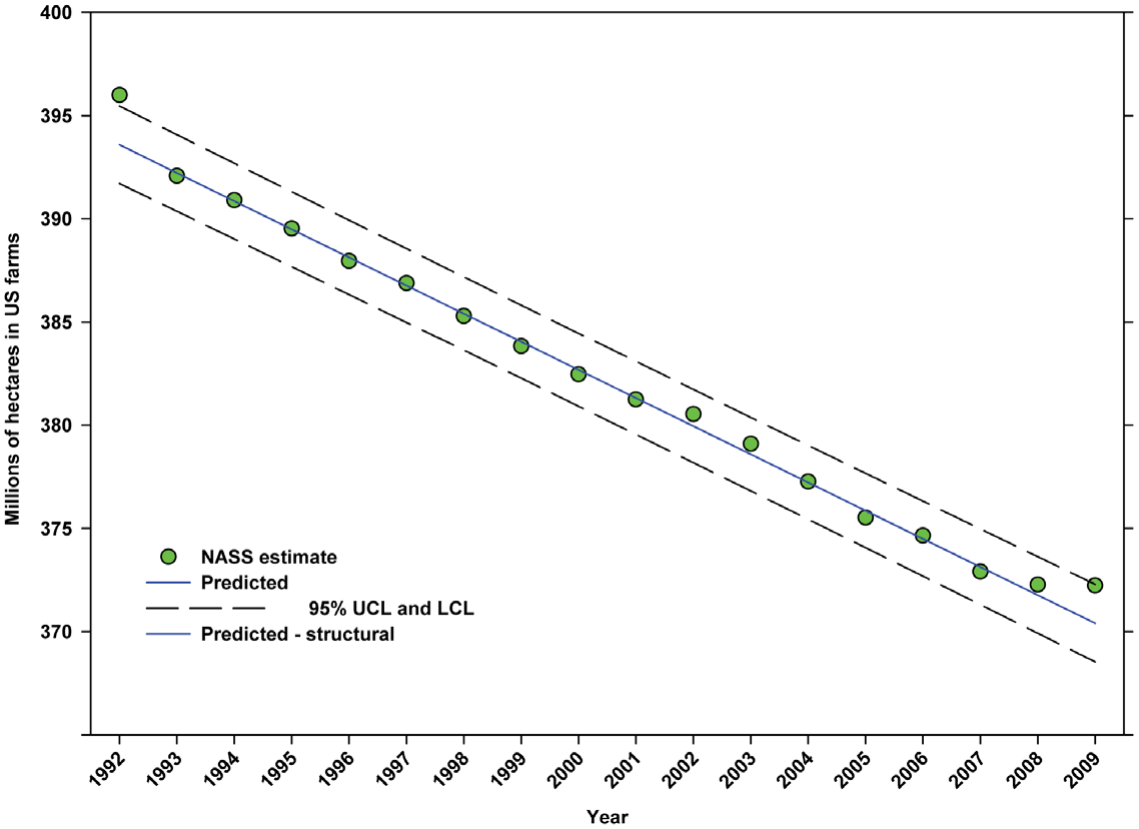
Total hectares in farms in the United States. Predicted values (blue) include adjustments for serial autocorrelation and are the same as the predicted – structural values (also blue) based solely on the structural elements of the model.

**Figure 4:**
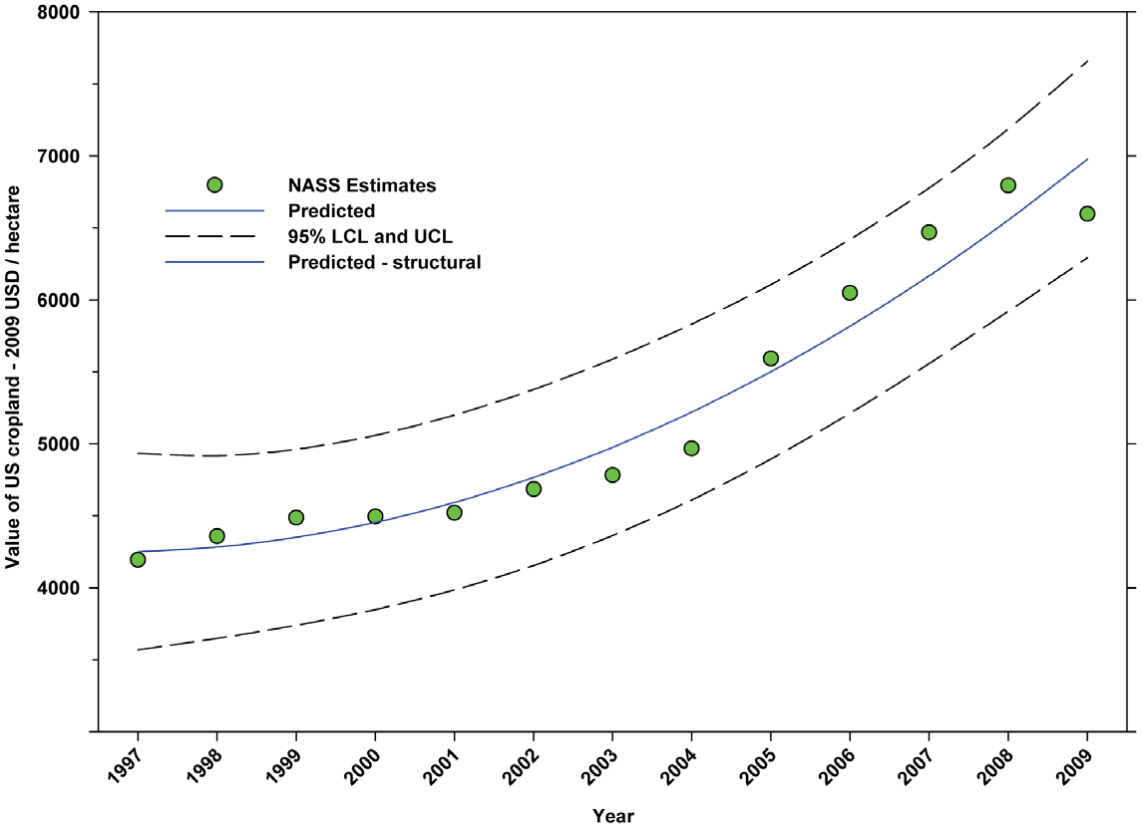
Value of cropland (2009 USD/hectare) in the United States. Predicted values (blue) include adjustments for serial autocorrelation and are the same as the predicted – structural values (also blue) based solely on the structural elements of the model.

Between 1992 and 2009, the US population increased in a linear manner from 256.51 million to 307.01 million, an increase of 19.69% (Fig. 2; Table 4). Between 1992 and 2009, the total number of hectares in farms declined from 395.99 million to 372.23 million, a decline of 6.00% (Fig. 3; Table 4). The value (2009 USD) of cropland rose from $4,194.81 per hectare in 1997 to $6,597.71 in 2009 (Fig. 4; Table 4), an increase of 57.28%.

**Table 4:** Results of the analyses of farm data in Table 1.

Variable	*y* -intercept	*B* _1_*x*	*B* _2_x^2^
**US population** 1			
Estimate ± SE	257.2553±0.4119	2.9637±0.0375	na
*t*	624.52	79.06	na
*P*>|*t*|	<0.0001	<0.0001	na
Total *R* ^2^	0.9997	na	na
**Number of hectares in farms** 1			
Estimate ± SE	393.5825±0.2048	−1.3633±0.0197	na
*t*	1921.86	−69.31	na
*P*>|*t*|	<0.0001	<0.0001	na
Total *R* ^2^	0.9900	na	na
**Value of cropland per hectare (2009 USD)**			
Estimate ± SE	4,251±424.8061	14.2960±118.9520	17.7343±7.8747
*t*	10.01	0.12	2.25
*P*>|*t*|	<0.0001	0.9043	0.0243
Total *R* ^2^	0.9534	na	na

1 millions; x = year; na = not applicable; *df* = 1 all effects.

### Results of Trend Analysis for Crops

#### Total number of cultivated hectares

**Figure 5:**
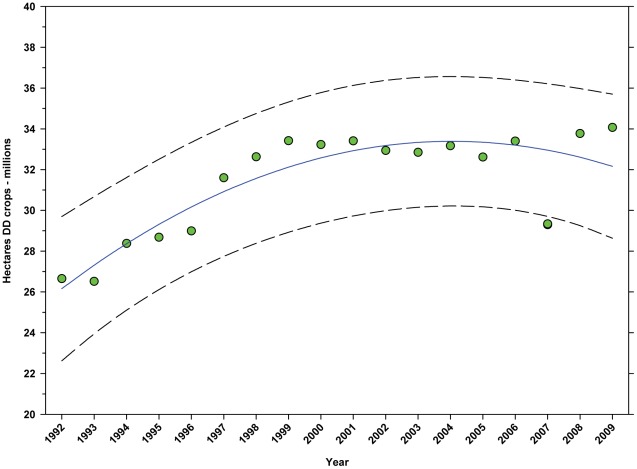
Number of hectares of directly dependent crops in the United States. Predicted values (blue) include adjustments for serial autocorrelation and are the same as the predicted – structural values (also blue) based solely on the structural elements of the model. DD = directly dependent.

**Figure 6:**
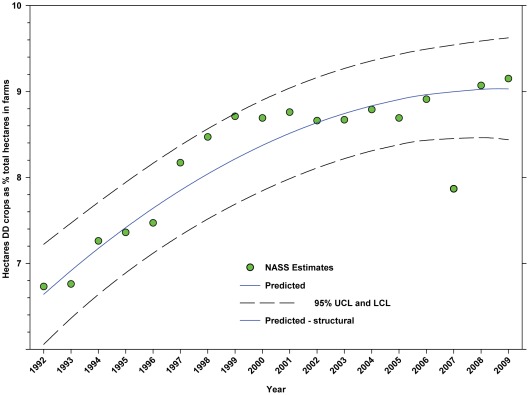
Hectares of directly dependent crops as a percentage of total hectares in farms. Predicted values (blue) include adjustments for serial autocorrelation and are the same as the predicted – structural values (also blue) based solely on the structural elements of the model. DD = directly dependent.

The number of hectares of DD crops increased from 26.65 million in 1992 to 34.07 million in 2009, an increase of 27.84% (Fig. 5; Table 5) with most of that increase coming between 1992 and 2004 followed by a slight decline. The reduction in 2007 (data not included in analysis) was due to a transient reduction in hectares in soybeans and, to a lesser extent, peanuts. The percentage of total hectares in farms used for the production of DD crops increased from 6.73% in 1992 to 9.15% in 2009, an increase of 35.96% (Fig. 6; Table 5). The rate of increase slowed around 1999 but maintains an upward trend.

**Table 5:** Results of the analyses of aggregate data summed over all crops for each year.

Variable	y-intercept	*B* _1_x	*B* _2_x^2^
**Number of hectares of DD crops** 1			
Estimate ± SE	26.1611±1.5039	1.2009±0.3781	−0.0499±0.0185
*t*	17.40	3.18	−2.69
*P*>|*t*|	<0.0001	<0.0015	<0.0071
Total *R* ^2^	0.7694	na	na
**Hectares of DD crops as a % total farm hectares**			
Estimate ± SE	6.6394±0.1189	0.2841±0.0347	−0.008438±0.001910
*t*	55.85	8.19	−4.42
*P*>|*t*|	<0.0001	<0.0001	<0.0001
Total *R* ^2^	0.9190	na	na
**Number of hectares of ID crops** 1			
Estimate ± SE	15.6404±0.1231	0.1617±0.0475	−0.0194±0.003536
*t*	127.04	3.40	−5.47
*P*>|*t*|	<0.0001	<0.0007	<0.0001
Total *R* ^2^	0.9185	na	na
**Hectares of ID crops as a % total farm hectares**			
Estimate ± SE	3.9633±0.0325	0.0602±0.0123	−0.005318±0.000909
*t*	121.96	4.90	−5.85
*P*>|*t*|	<0.0001	<0.0001	<0.0001
Total *R* ^2^	0.8898	na	na
**Production DD crops** 1			
Estimate ± SE	97.0807±4.1440	3.8967±1.2232	−0.1403±0.0683
*t*	23.43	3.19	−2.05
*P*>|*t*|	<0.0001	<0.0014	<0.0400
Total *R* ^2^	0.6477	na	na
**Production ID crops** 1			
Estimate ± SE	108.2111±2.8688	1.1554±0.6792	−0.1019±0.0372
*t*	37.72	1.70	−2.74
*P*>|*t*|	<0.0001	<0.0889	<0.0061
Total *R* ^2^	0.5777	na	na

1 millions; DD = directly dependent crops; ID = indirectly dependent crops; x = year; na = not applicable; *df* = 1 all effects.

**Figure 7:**
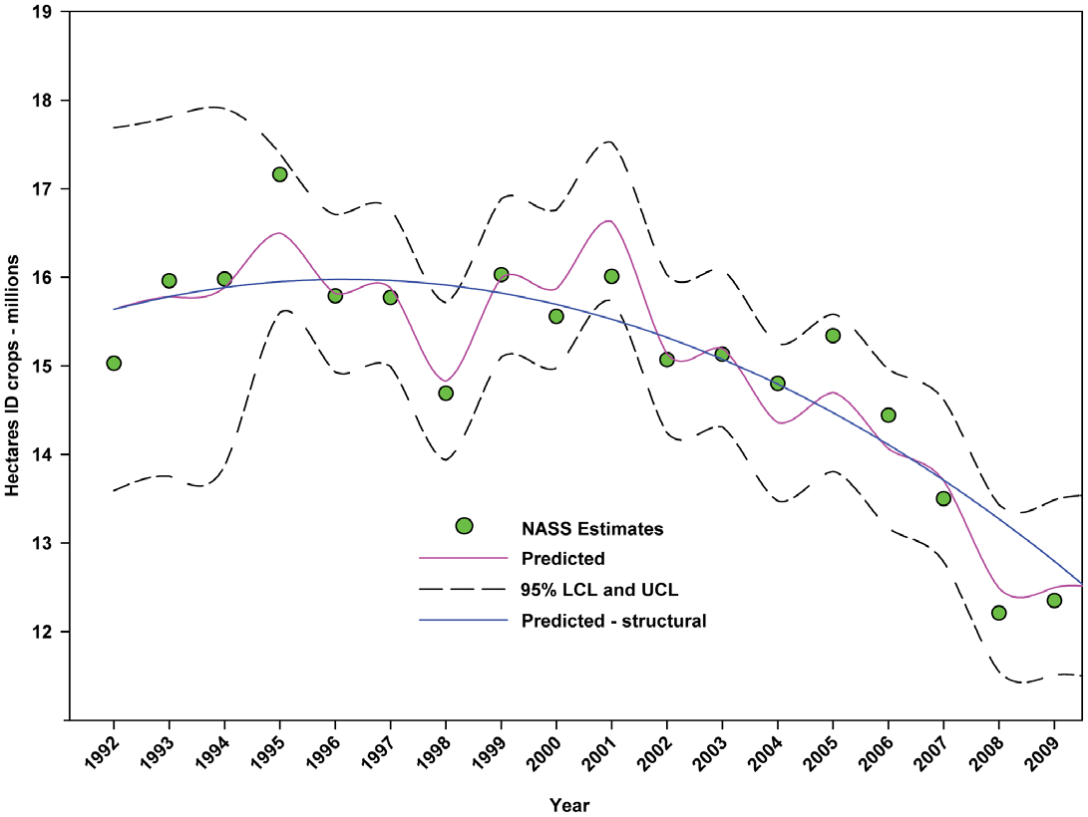
Number of hectares of indirectly dependent crops in the United States. Predicted values (pink) include adjustments for serial autocorrelation. Predicted – structural values (blue) are based solely on the structural elements of the model. ID = indirectly dependent.

**Figure 8:**
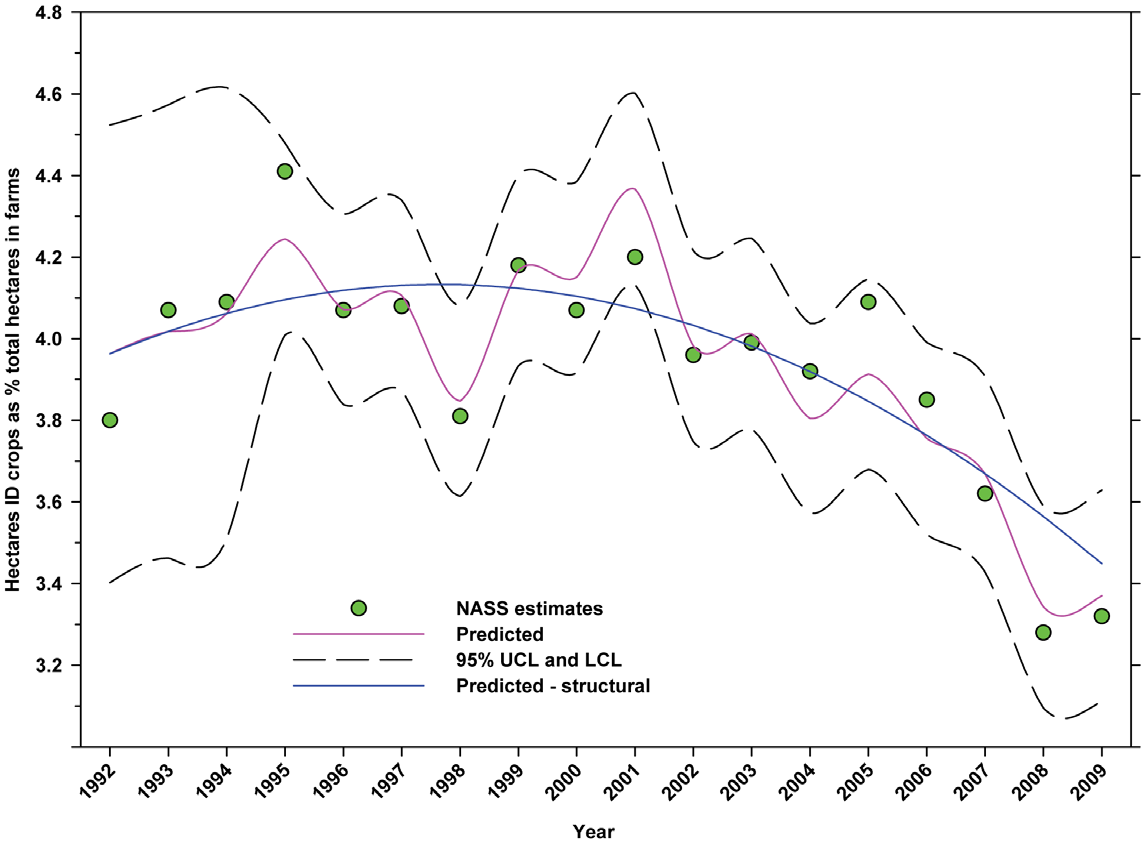
Hectares of indirectly dependent crops as a percentage of total hectares in farms. Predicted values (pink) include adjustments for serial autocorrelation. Predicted – structural values (also blue) are based solely on the structural elements of the model. ID = indirectly dependent.

Over the same period, the number of hectares of ID crops declined from 15.03 million to 12.35 million, a decline of 17.83%. There was a slight increase between 1992 and 1996 followed by an accelerating decline thereafter (Fig. 7; Table 5). The number of hectares used for ID crops as a percentage of total hectares in farms declined from 3.80% in 1992 to 3.32% in 2009, a decline of 12.63% (Fig. 8; Table 5).

#### Total production

**Figure 9:**
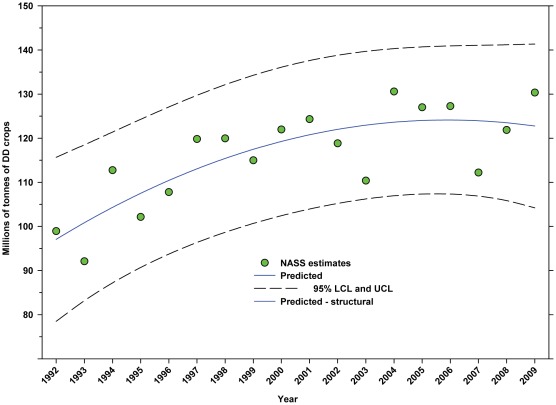
Total production (tonnes) of directly dependent crops. Predicted values (blue) include adjustments for serial autocorrelation and are the same as the predicted – structural values (also blue) based solely on the structural elements of the model. DD = directly dependent.

**Figure 10:**
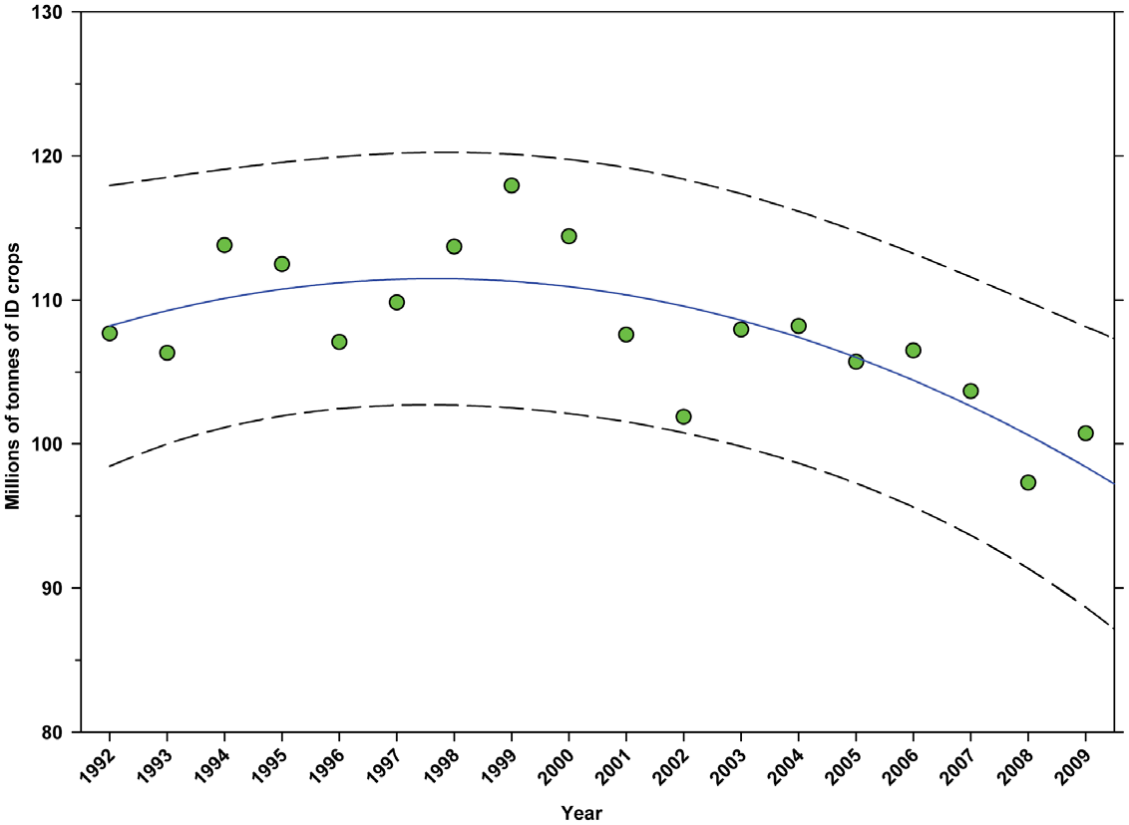
Total production (tonnes) of indirectly dependent crops. Predicted values (blue) include adjustments for serial autocorrelation and are the same as the predicted – structural values (also blue) based solely on the structural elements of the model. ID = indirectly dependent.

There was an increase in the production of DD crops from 98.93 million tonnes in 1992 to 130.34 million tonnes in 2009, an increase of 31.75% (Fig. 9; Table 5), although the rate of increase slowed around 1999. Production of ID crops decreased over the same period from 107.67 million tonnes in 1992 to 100.74 million tonnes in 2009, a decline of 6.44% (Fig. 10; Table 5). Production increased between 1992 and 1999 but declined thereafter.

#### Yield

**Figure 11:**
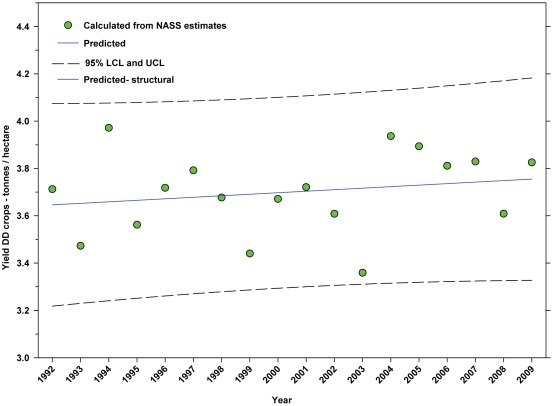
Yield of directly dependent crops. Predicted values (blue) include adjustments for serial autocorrelation and are the same as the predicted – structural values (also blue) based solely on the structural elements of the model. DD = directly dependent.

**Figure 12:**
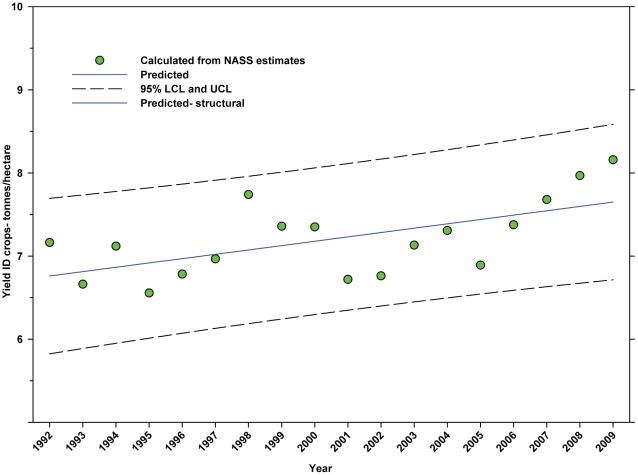
Yield of indirectly dependent crops. Predicted values (blue) include adjustments for serial autocorrelation and are the same as the predicted – structural values (also blue) based solely on the structural elements of the model. ID = indirectly dependent.

For the period from 1992–2009, the yield of DD crops ranged between 3.97 tonnes per hectare (1994) and 3.36 tonnes per hectare (2003); but there was no significant trend (Fig. 11; Table 6). For the same period, the yield of ID crops exhibited a significant increasing linear trend from 7.16 tonnes per hectare in 1992 to 8.16 tonnes/hectare in 2009 (Fig. 12; Table 6).

**Table 6:** Results of the analyses of aggregate data summed over all crops for each year.

Variable	y-intercept	*B* _1_x	*B* _2_x^2^	
**Yield** 1 **of DD crops**				
Estimate ± SE	3.6460±0.0832	0.006412±0.009121	na	
*t*	43.82	0.70	na	
*P*>|*t*|	<0.0001	<0.4821	na	
Total *R* ^2^	0.0429	na	na	
**Yield** 1 **of ID crops**				
Estimate ± SE	6.7602±0.1760	0.0524±0.0168	na	
*t*	38.42	3.11	na	
*P*>|*t*|	<0.0001	0.0019	na	
Total *R* ^2^	0.3701	na	na	

1 Yield calculated as tonnes/hectare from production data and cultivated hectares; DD = directly dependent crops; ID = indirectly dependent crops; *df* = 1 all effects; na = not applicable.

#### Response to changes in US population

**Figure 13:**
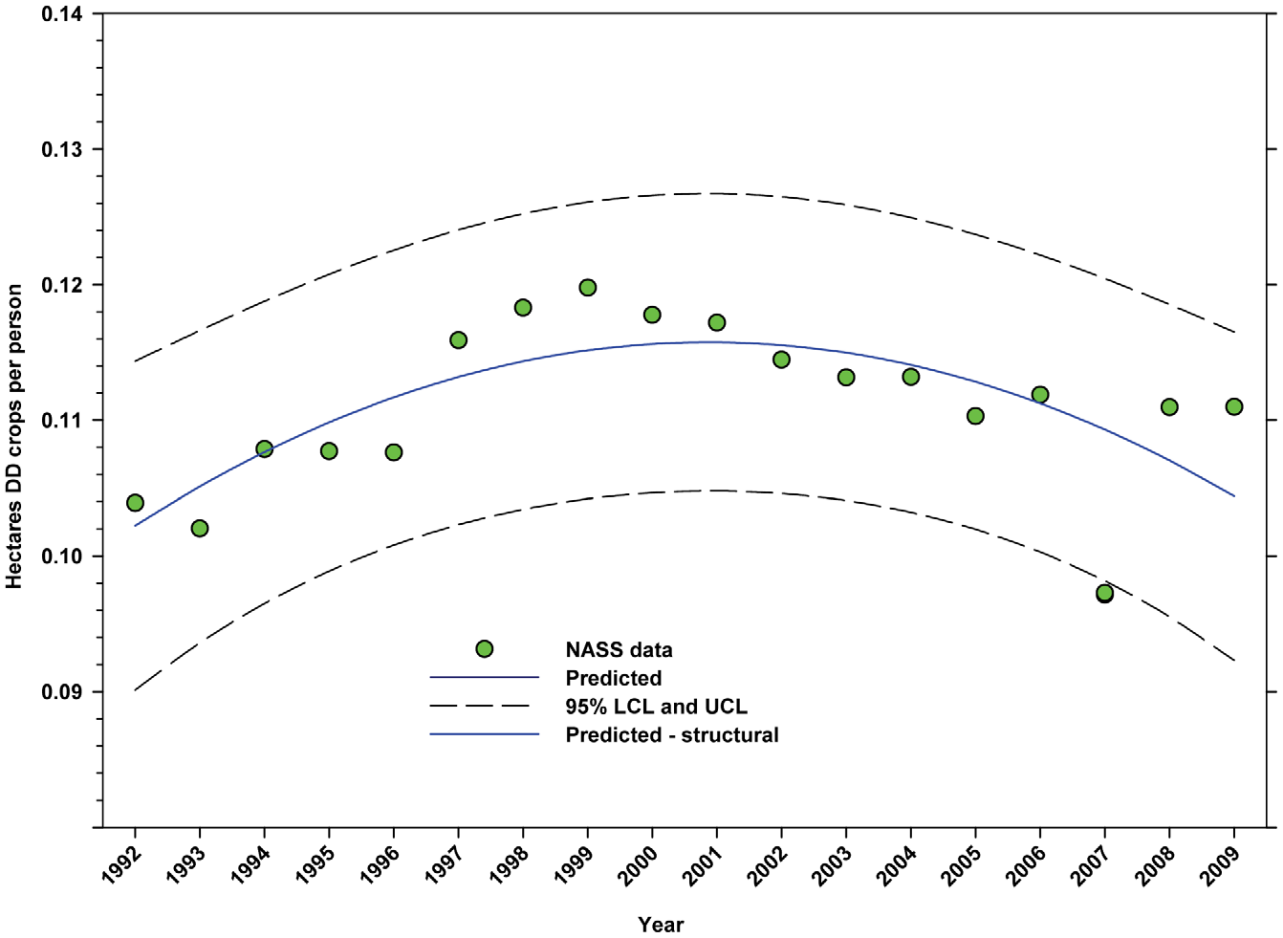
Hectares of directly dependent crops per person in the United States. Predicted values (blue) include adjustments for serial autocorrelation and are the same as the predicted – structural values (also blue) based solely on the structural elements of the model. DD = directly dependent.

**Figure 14:**
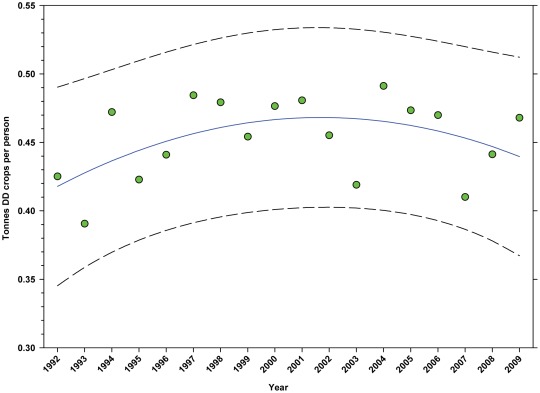
Tonnes of directly dependent crops per person in the United States. Predicted values (blue) include adjustments for serial autocorrelation and are the same as the predicted – structural values (also blue) based solely on the structural elements of the model. DD = directly dependent.

**Figure 15:**
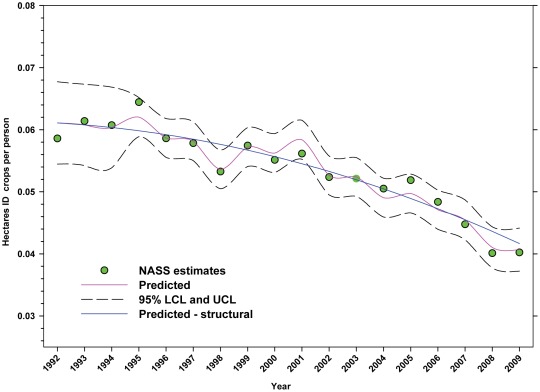
Hectares of indirectly dependent crops per person in the United States. Predicted values (blue) include adjustments for serial autocorrelation and are the same as the predicted – structural values (also blue) based solely on the structural elements of the model. ID = indirectly dependent.

**Figure 16:**
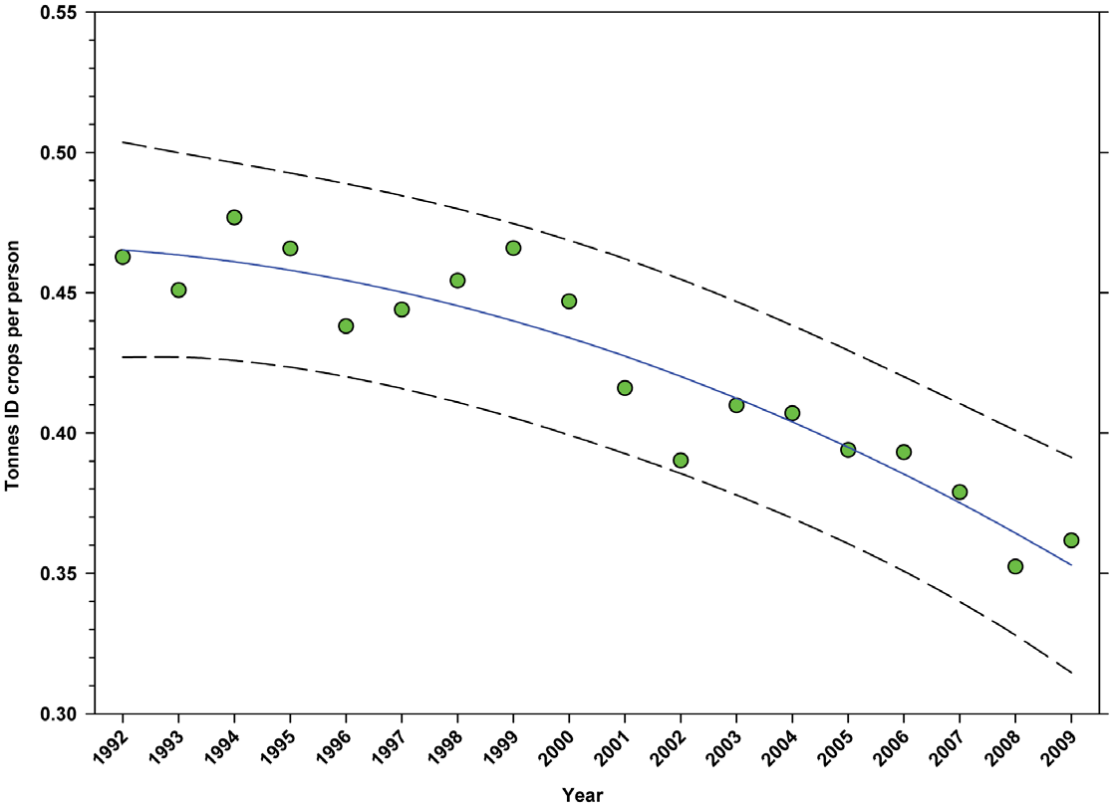
Tonnes of indirectly dependent crops per person in the United States. Predicted values (blue) include adjustments for serial autocorrelation and are the same as the predicted – structural values (also blue) based solely on the structural elements of the model. ID = indirectly dependent.

The number of hectares of DD crops expressed as hectares per person (Table 2) rose from 1992 to 1999 when it peaked at 0.1198, but declined to 0.1110 by 2009 (Fig. 13; Table 7). The production of DD crops expressed as tonnes per person (Table 3) rose from 1992 to 2001 when it reached 0.48, but has since trended downward (Fig. 14; Table 7). The number of hectares of ID crops expressed as hectares per person (Table 2) declined steadily from 1992 through 2009 from 0.06 to 0.04 (Fig. 15; Table 7). Production of ID crops expressed as tonnes per person followed a similar pattern, reaching a high of 0.48 in 1994 and declining to 0.36 by 2009 (Fig. 16; Table 7).

**Table 7:** Results of analyses of aggregate date summed over all crops for each year.

Variable	y-intercept	*B* _1_x	*B* _1_x
**Hectares of DD crops per person**			
Estimate ± SE	0.1022±0.004824	0.003047±0.001218	−0.000172±0.0000601
*t*	21.20	2.50	−2.86
*P*>|*t*|	<0.0001	<0.0001	0.0042
Total *R* ^2^	0.5084	na	na
**Hectares of ID crops per person**			
Estimate ± SE	0.0611±0.000421	−0.000266±0.000178	−0.000052±0.0000136
*t*	145.06	−1.50	−3.79
*P*>|*t*|	<0.0001	<0.1356	<0.0001
Total *R* ^2^	0.9639	na	na
**Tonnes of DD crops per person**			
Estimate ± SE	0.4179±0.0152	0.0.0104±0.004644	−0.000535±0.000265
*t*	27.55	2.24	−2.02
*P*>|*t*|	<0.0001	<0.0253	<0.0432
Total *R* ^2^	0.2573	na	na
**Tonnes of ID crops per person**			
Estimate ± SE	0.4653±0.0106	−0.001517±0.002586	−0.000299±0.000146
*t*	43.99	−0.59	−2.05
*P*>|*t*|	<0.0001	<0.5576	<0.0407
Total *R* ^2^	0.8793	na	na

DD = directly dependent crops; ID = indirectly dependent crops; x = year; na = not applicable; *df* = 1 all effects.

#### Total value (2009 USD)

**Figure 17:**
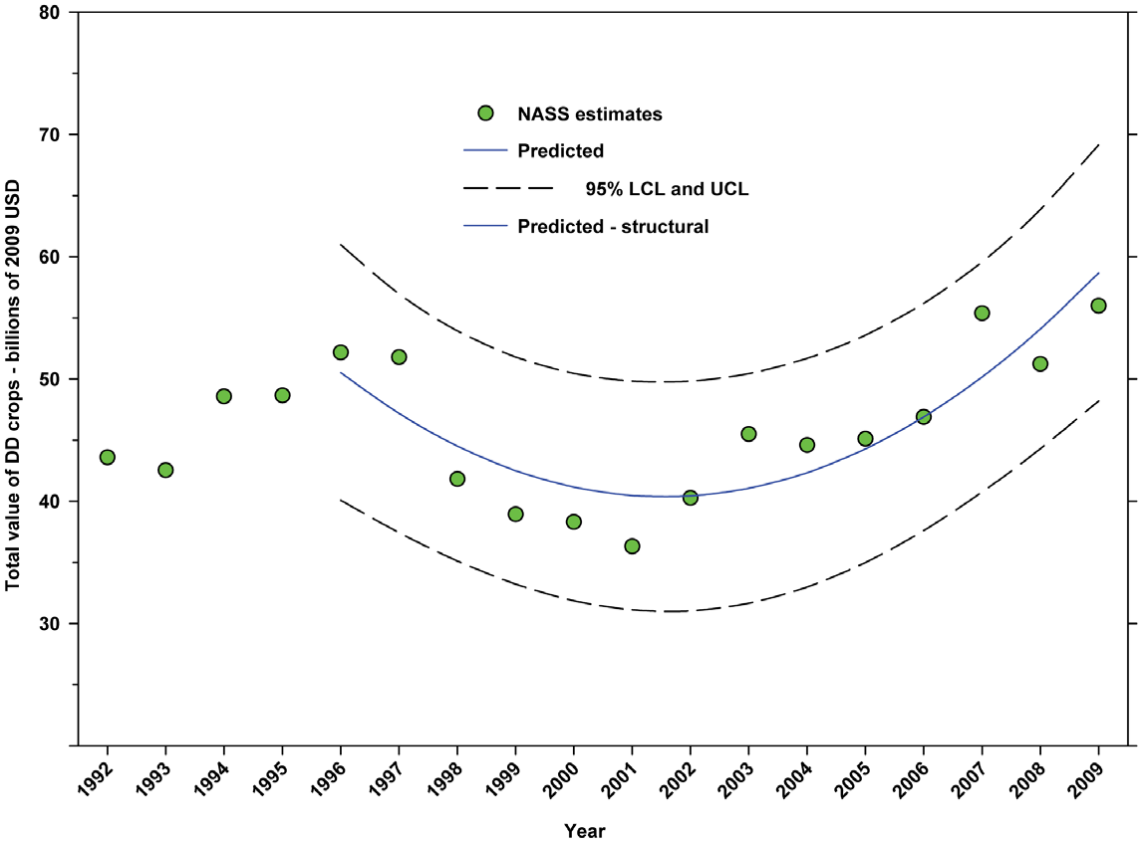
Total value of directly dependent crops. Predicted values (blue) include adjustments for serial autocorrelation and are the same as the predicted – structural values (also blue) based solely on the structural elements of the model. DD = directly dependent.

**Figure 18:**
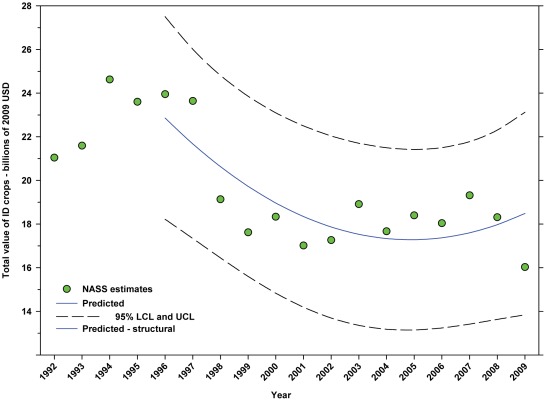
Total value of indirectly dependent crops. Predicted values (blue) include adjustments for serial autocorrelation and are the same as the predicted – structural values (also blue) based solely on the structural elements of the model. DID = indirectly dependent.

The total value of DD crops decreased from $52.18 B in 1996 to $36.30 B in 2001, but increased thereafter, reaching $55.99 B in 2009, an increase of 7.30% since 1996 and 54.24% from the low in 2001 (Fig. 17; Table 8). The total value of ID crops declined from $23.95 B in 1996 through 2001, but has since increased, reaching $16.03 B in 2009. Overall, this reflects a decline of 33.07% (Fig. 18; Table 8); however, the value of $16.03 B in 2009 was well below the trend line, and the value in 2008 was $18.31 B.

**Table 8:** Statistics for aggregate values from 1996–2009.

Variable	*y* -intercept	*B* _1_x	*B* _2_x^2^
**Total value DD crops - billions of 2009 USD**			
Estimate ± SE	50.5281±2.3798	−3.6651±0.8850	0.3302±0.0655
*t*	21.23	−4.14	5.04
*P*>|*t*|	<0.0001	<0.0001	<0.0001
Total *R* ^2^	0.7475	na	na
**Total value ID crops - billions of 2009 USD**			
Estimate ± SE	22.8607±0.9418	−1.2576±0.3803	0.0708±0.0270
*t*	24.27	−3.31	2.62
*P*>|*t*|	<0.0001	<0.0021	<0.0088
Total *R* ^2^	0.5985	na	na
**Value DD crops due to insect pollination - billions of 2009 USD**			
Estimate ± SE	13.6784±0.7478	−0.6670±0.2633	0.0677±0.0184
*t*	18.29	−2.53	3.68
*P*>|*t*|	<0.0001	<0.0113	<0.0002
Total *R* ^2^	0.6539	na	na
**Value ID crops due to insect pollination -billions of 2009 USD**			
Estimate ± SE	16.0180±0.7502	−0.9604±0.1861	0.0614±0.0115
*t*	21.35	−5.16	5.34
*P*>|*t*|	<0.0001	<0.0001	<0.0001
Total *R* ^2^	0.5206	na	na

DD = directly dependent crops; ID = indirectly dependent crops; x = year; na = not applicable; *df* = 1 all effects.

#### Total value attributed to insect pollination (2009 USD)

**Figure 19:**
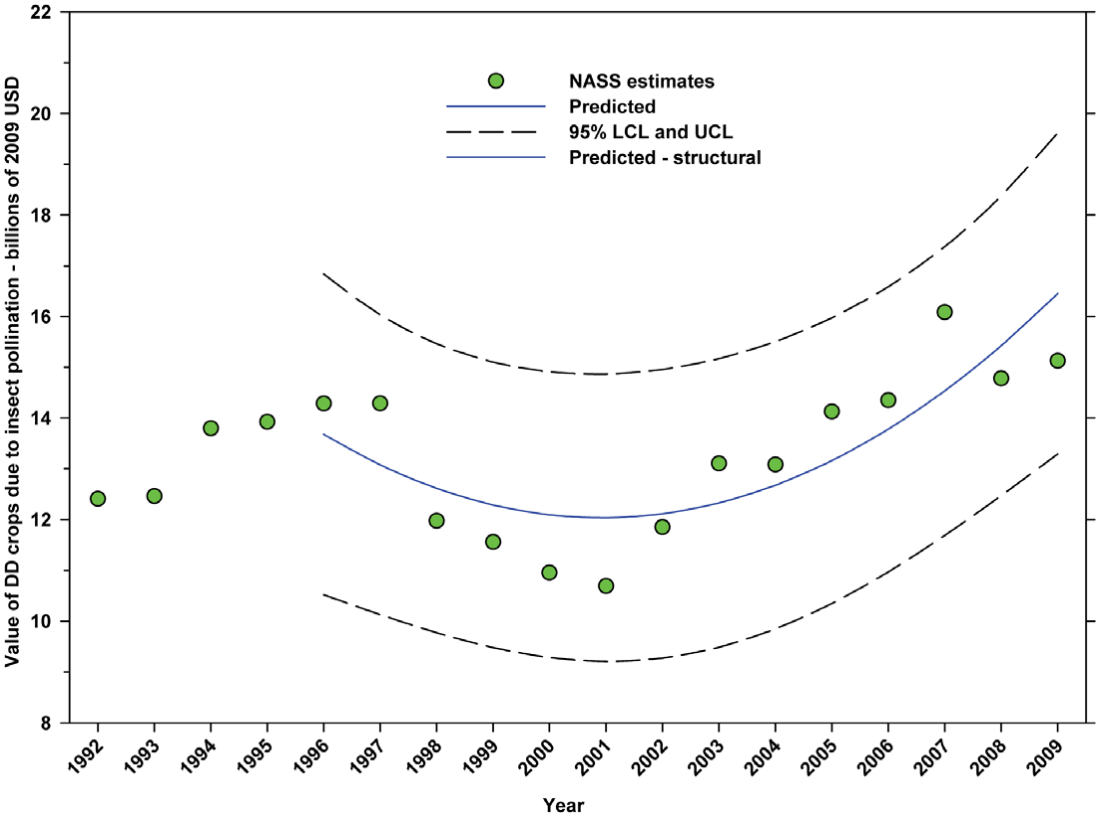
Value of directly dependent crops attributed to insect pollination. Predicted values (blue) include adjustments for serial autocorrelation and are the same as the predicted – structural values (also blue) based solely on the structural elements of the model. DD = directly dependent.

**Figure 20:**
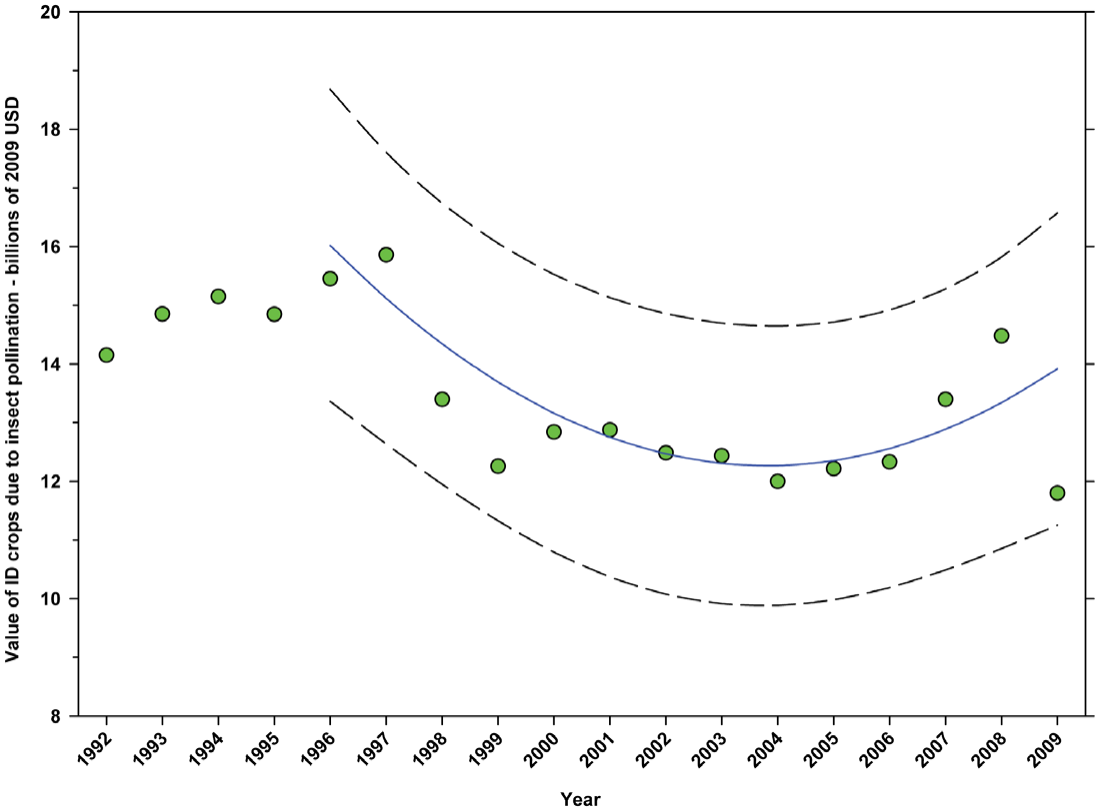
Value of indirectly dependent crops attributed to insect pollination. Predicted values (blue) include adjustments for serial autocorrelation and are the same as the predicted – structural values (also blue) based solely on the structural elements of the model. ID = indirectly dependent.

The value of DD crops attributed to insect pollination decreased from $14.29 B in 1996 to $10.69 B in 2001, but increased thereafter, reaching $15.12 B in 2009, an increase of 41.44% since the low in 2001 (Fig. 19; Table 8). The value of ID crops attributed to insect pollination declined from $15.45 B in 1996 to $11.80 B in 2009, a decline of 23.63% (Fig. 20; Table 8); although the 2009 value was below the trend line. This metric has increased since 2004.

#### Total value attributed to honey bees (2009 USD)

**Figure 21:**
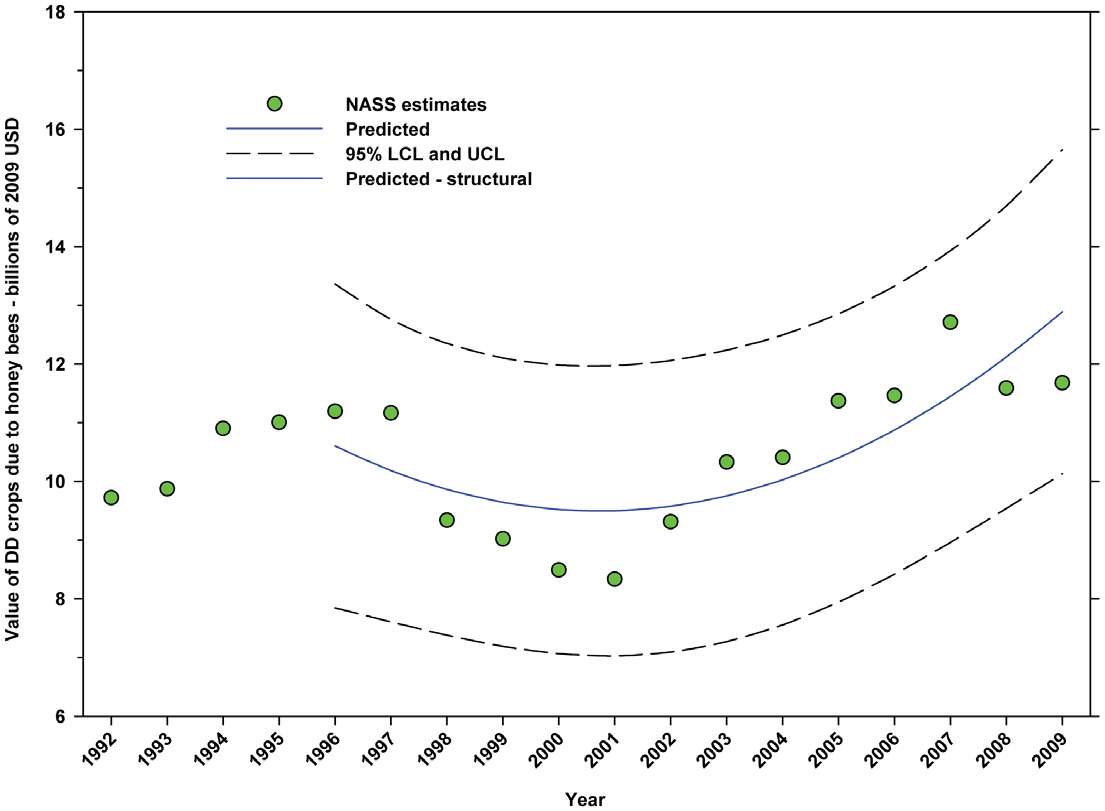
Value of directly dependent crops attributed to honey bees (*A. mellifera*). Predicted values (blue) include adjustments for serial autocorrelation and are the same as the predicted – structural values (also blue) based solely on the structural elements of the model. DD = directly dependent.

**Figure 22:**
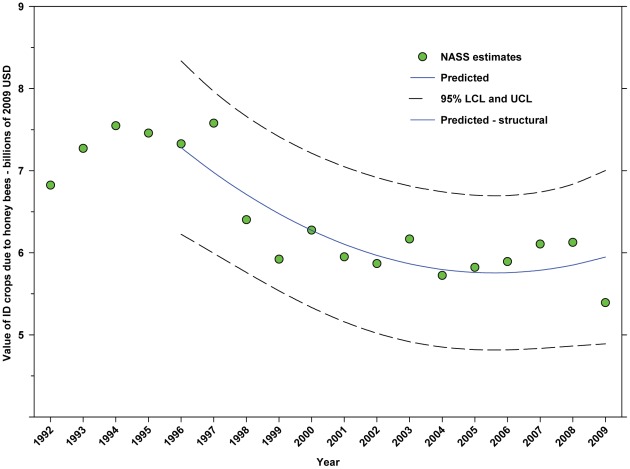
Value of indirectly dependent crops attributed to honey bees (*A. mellifera*). Predicted values (blue) include adjustments for serial autocorrelation and are the same as the predicted – structural values (also blue) based solely on the structural elements of the model. ID = directly dependent.

The value of DD crops attributed to honey bee pollination decreased from $11.20 B in 1996 to $8.33 B in 2001, but increased thereafter, reaching $11.68 B in 2009, an increase of 40.22% from the low in 2001 (Fig. 21; Table 9). The value of ID crops attributed to honey bees decreased from $7.33 B in 1996 to $5.39 B in 2009, a decrease of 26.47% (Fig. 22; Table 9). The decline occurred between 1996 and 2004 and values trended upward thereafter with the exception of 2009 which was below the trend line.

**Table 9:** Statistics for aggregate values from 1996–2009.

Variable	*y* -intercept	*B* _1_x	*B* _2_x^2^
**Value DD crops due to ** ***A. mellifera*** ** - billions of 2009 USD**			
Estimate ± SE	10.6028±0.6554	−0.4686±0.2261	0.0496±0.0157
*t*	16.18	−2.07	3.15
*P*>|*t*|	<0.0001	<0.0382	<0.0016
Total *R* ^2^	0.5972	na	na
**Value ID crops due to ** ***A. mellifera -*** **billions of 2009 USD**			
Estimate ± SE	7.2810±0.2377	−0.3186±0.1074	0.0166±0.007319
*t*	30.63	−2.97	2.27
*P*>|t|	<0.0001	<0.0030	0.0232
Total *R* ^2^	0.6933	na	na
**Value ID crops due to ** ***M. rotundata -*** ** billions of 2009 USD**			
Estimate ± SE	6.8753±0.7324	−0.4772±0.2132	0.0324±0.0142
*t*	9.39	−2.24	2.29
*P*>|t|	<0.0001	<0.0252	<0.0221
Total *R* ^2^	0.4016	na	na
**Value DD crops due to other non-** ***Apis*** ** insect pollinators - billions of 2009 USD**			
Estimate ± SE	3.0755±0.1126	−0.1988±0.0410	0.0182±0.003054
*t*	27.32	−4.84	5.95
*P*>|t|	<0.0001	≪0.0001	<0.0001
Total *R* ^2^	0.8269	na	na
**Value ID crops due to other non-** ***Apis*** ** insect pollinators - billions of 2009 USD**			
Estimate ± SE	1.5184±0.0528	−0.0737±0.0233	0.004328±0.001627
*t*	28.75	−3.16	2.66
*P*>|t|	<0.0001	<0.0016	<0.0078
Total *R* ^2^	0.6265	na	na

DD = directly dependent crops; ID = indirectly dependent crops; x = year; na = not applicable; *df* = 1 all effects.

#### Total value attributed to M. rotundata (2009 USD)

**Figure 23:**
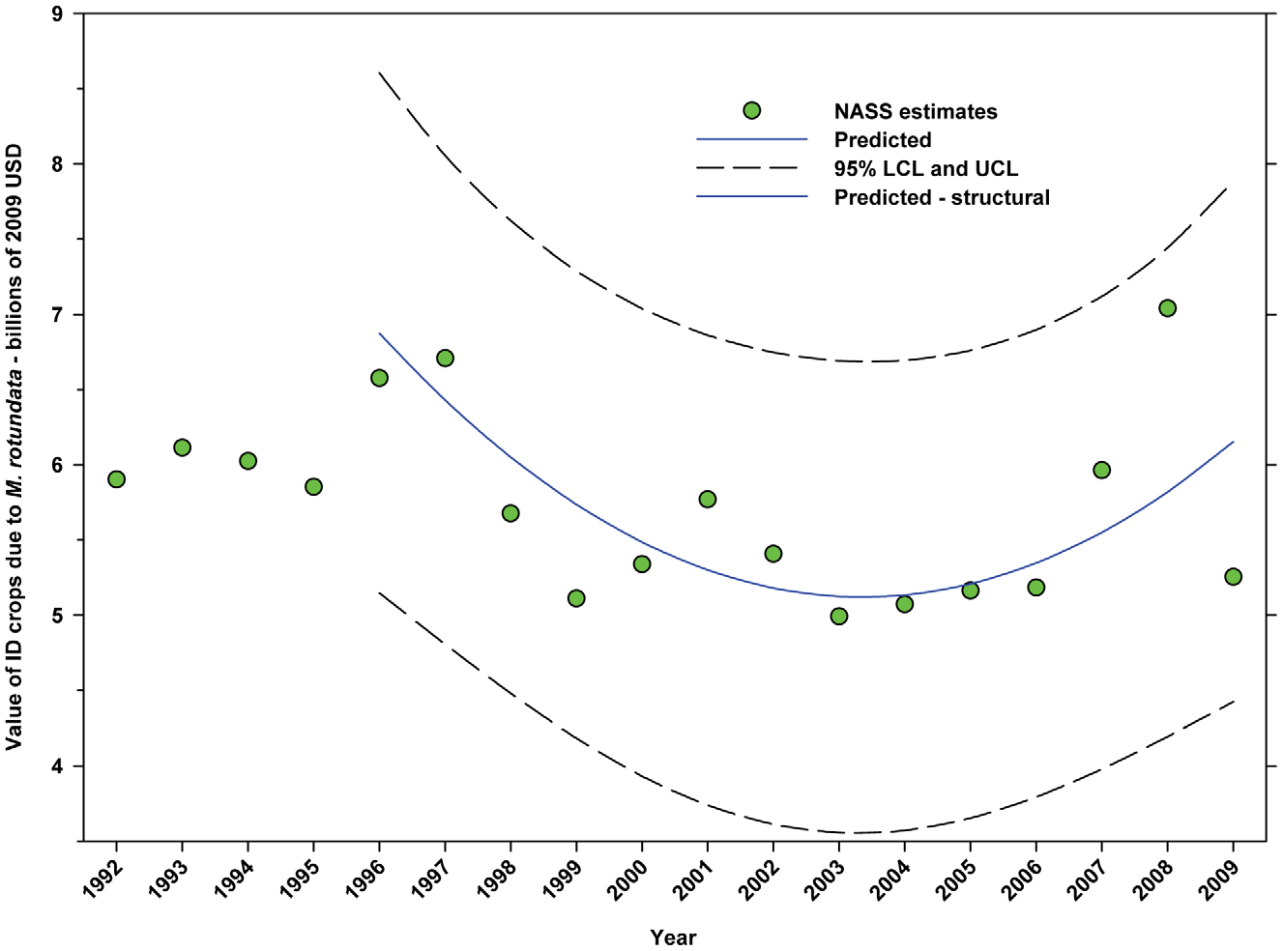
Value of indirectly dependent crops attributed to alfalfa leafcutter bees (*M. rotundata*). Predicted values (blue) include adjustments for serial autocorrelation and are the same as the predicted – structural values (also blue) based solely on the structural elements of the model. ID = indirectly dependent.

The leafcutter bee is responsible for the major portion of alfalfa seed (data not available on annual basis) and, indirectly, alfalfa hay. The value of alfalfa hay attributed to leafcutter bees ranged between $4.99 B (2003) and $7.04 B (2008) (Fig. 23; Table 9) with a decline to $5.26 B in 2009. With that exception, the overall trend has been increasing since 2003.

#### Total value attributed to other insects (2009 USD)

**Figure 24:**
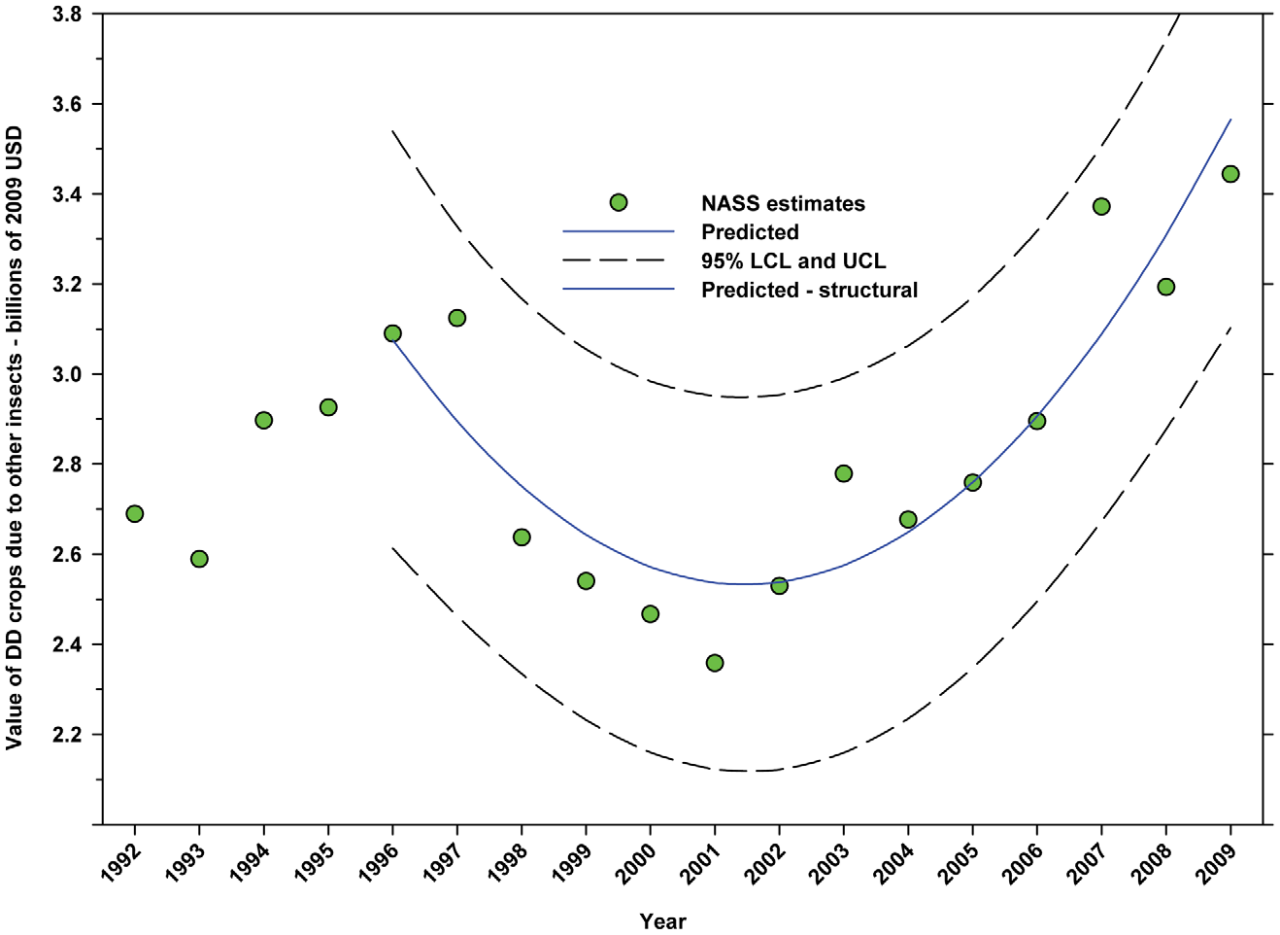
Value of directly dependent crops attributed to other insects. Predicted values (blue) include adjustments for serial autocorrelation and are the same as the predicted – structural values (also blue) based solely on the structural elements of the model. DD = directly dependent.

**Figure 25:**
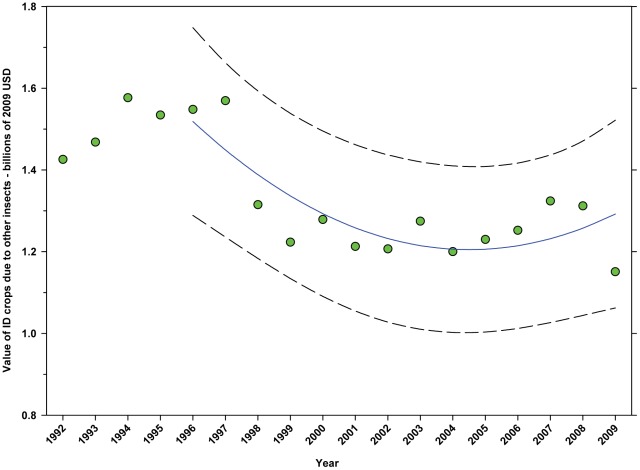
Value of indirectly dependent crops attributed to other insects. Predicted values (blue) include adjustments for serial autocorrelation and are the same as the predicted – structural values (also blue) based solely on the structural elements of the model. ID = indirectly dependent.

The value of DD crops attributable to insect pollinators other than honey bees or leafcutter bees decreased from $3.09 B in 1996 to $2.36 B in 2001, but increased thereafter, reaching $3.44 B in 2009, increase of 45.76% from the low in 2001 (Fig. 24; Table 9). The value of ID crops attributable to insect pollination other than honey bees or leafcutter bees decreased over the same period from $1.55 B to $1.15 B, a decline of 25.81% (Fig. 25; Table 9). The decline occurred between 1996 and 2000; values have been relatively stable or increasing since.

### Statistics for individual crops for 2002 and 2007

Data for individual crops for 2002 and 2007 are presented as Text S2. Values for production, cultivated hectares and value of production are slightly greater than those shown in the trend analyses because they include data on alfalfa and non-alfalfa legume seed, pumpkins and squash, none of which were available for the trend analyses. Data for 2010 (data for legume seed production not available) are presented as Text S3.

### Decline in the number of honey bee colonies and the pollinator shortage

An analysis of the decline in the number of honey bee colonies, the number of colonies required to meet current recommendations (colonies/hectare) and their relationship to the adequacy of pollination services are presented as Text S4.

#### Other hive products

National data on the US honey bee queen and package industries, nucs (starter colonies), pollination rental fees and hive products other than honey are not available. I place a tentative estimate of $300–$500 million (2009 USD) on the value of those products and services but do not include that estimate in any calculation.

## Discussion

### Summary of data for DD Crops

The number of cultivated hectares of DD crops increased from 26.65 million in 1992 (first year for production, cultivated area and yield data in this study) to 34.07 million in 2009, an increase of 27.84% (Fig. 5). As a percentage of total farm hectares, this represents an absolute increase from 6.73% to 9.15% and a relative increase of 35.96% (Fig. 6); this growth occurred as the price of cropland was also rising (Fig. 4), reflecting the relatively high value of those crops [28]. Production increased from 98.93 million tonnes in 1992 to 130.34 million tonnes in 2009, an increase of 31.75% (Fig. 9). The majority of increases in each metric occurred between 1992 and 2000/2001 with flat or significantly reduced rates of increase thereafter. Aggregate yield was flat over the study period (Fig. 11). US trends differ somewhat from those in other developed countries that show steady increases in yield and cultivated acres and more modest but continuing increases in production over the same period. They differ significantly from trends in the developing world where those metrics continue to increase rapidly [29], [31]. The cultivated area and production of DD crops in the US, measured as hectares or tonnes per person, kept pace with growth in the population through 2000–2001, but neither kept pace thereafter (Fig. 13 and Fig. 14) even though per capita consumption of fruits and vegetables remained relatively steady [2]–[5]. These results are consistent with land use patterns reflecting rising cropland values and growing access to imported food [132]–[135].

The total value (2009 USD) of DD crops declined between 1996 (first year for value data in this study) and 2001 from $52.18 B to $36.30 B, but rose thereafter, reaching $55.99 B in 2009 (Fig. 17), an increase of 54.24% from 2001. Revenues attributed to insect pollination decreased from $14.29 B in 1996 to $10.69 B in 2001, but increased thereafter, reaching $15.12 B in 2009 (Fig. 19), an increase of 41.44% from 2001. Revenues attributed to honey bees decreased from $11.20 B in 1996 to $8.33 B in 2001, but increased thereafter, reaching $11.68 B in 2009 (Fig. 21), an increase of 40.22% since 2001. Revenues attributed to insect pollinators other than honey bees decreased from $3.09 B in 1996 to $2.36 B in 2001, but increased thereafter, reaching $3.44 B in 2009 (Fig. 24), an increase of 45.76% from 2001.

### Summary of data for ID Crops

The number of hectares used for production of ID crops was relatively steady between 1992 and the early 2000's, but declined from a high of 16.03 million hectares in 1999 to 12.35 million in 2009, a reduction of 22.96% (Fig. 7). As a percentage of total farm hectares, this represents an absolute decline from 3.80% to 3.32% and a relative decline of 12.63% (Fig. 8). This may be due, in part, to the rising value of cropland (Fig. 4) and the fact that the value of ID crops tends to be less than that of DD crops [28]. Total production followed a similar pattern, declining from a high of 117.94 tonnes in 1999 to 100.74 tonnes in 2009, a reduction of 14.58% (Fig. 10). The fact that the decline in production (14.58%) was less than the decline in hectares (22.96%) can be explained, in part, by the increase in yield over the same period (Fig. 12). US trends are similar to those in other developed countries that show steady increases in yields of ID crops with declines in both production and cultivated area over the same period. They differ significantly from trends in the developing world where yield and production continue to increase rapidly while cultivated area also continues to increase, albeit at a somewhat slower rate [29], [31]. Trend analysis revealed that neither hectares nor production of ID crops, measured as hectares or tonnes per person, kept pace with the growth in the US population (Fig. 15 and Fig. 16). As with DD crops, these results are consistent with land use patterns reflecting increasing cropland values and the availability of imported food [132]–[135].

The total value of ID crops declined from $23.95 B in 1996 to $17.01 B in 2001, but increased thereafter, ranging between $16.02 B (2009) and $19.32 B (2007) (Fig. 18). Revenues attributed to insect pollination declined from $15.45 B to $11.99 B between 1996 and 2004, but have since risen with the exception of 2009 which saw a large decline from $14.48 B in 2008 to $11.80 B in 2009 (Fig. 20). Revenues attributed to honey bees declined from $7.33 B in 1996 to $5.39 B in 2009 with values otherwise running between $6.40 B and $5.39 B since 1998 (Fig. 22). The value attributed to insect pollinators other than honey bees or leafcutter bees decreased over the same period from $1.55 B to $1.15 B (Fig. 25), although 2009 was well below the trend line. The value of alfalfa hay attributed to leafcutter bees ranged between $4.99 B (2003) and $ 7.04 B (2008) with decreasing values between 1996 and 2003 and increasing values thereafter (Fig. 23).

### Dependency coefficients and value estimates

Two topics that influence efforts to quantify the contributions of insect pollinators to US agriculture are: 1) the accuracy of the dependency coefficients for partitioning value among the various pollinators [16], [136], and 2) the interpretation of value [58], [137]. With the exception of the coefficients for alfalfa seed and hay production, dependency coefficients used here come from Robinson, Nowogrodzki and Morse [59], [60] who based estimates on a review of 275 studies conducted prior to 1989. To the degree that those estimates are sensitive to changes in management practices (e.g., selection of crop varieties; the use of pesticides, fertilizers and growth regulators; the size of fields or orchards) and local environmental factors (e.g., land-use patterns; the abundance and diversity of non-*Apis* pollinators), they may not reflect the current contributions of the various pollinator groups. In addition, the methodology of those studies was not usually designed to capture the contributions of non-*Apis* bees and other insects. Current research emphasizes the diversity and abundance of pollinator species combined with measures of blossom density, visits per blossom, pollen grains deposited per visit and yield [138]–[140]. Such studies promise to increase the accuracy of estimates of dependency coefficients in a variety of landscape situations.

The second topic involves the estimation of value. Most studies estimate the value of honey bee pollination as the increase in gross farmgate value over and above that expected in the absence of honey bees (see Mburu and colleagues [137] for discussion of valuation methods). However, this method has certain limitations. It focuses on gross rather than net income [141]; and it neglects to account for other inputs such as chemicals, fuel, equipment, labor, water and land [142]. Further, it differs from the way value is often used by economists because it does not account for the response of markets to changes in supply [28], [58], [142]–[144]. If honey bee populations were reduced or eliminated, it is argued, markets would adjust through some combination of factors, including the use of alternative pollinators, changes in the price of goods, and other changes in grower and consumer behavior, until a new equilibrium is established. The actual value of honey bees would be the difference between the original farmgate revenues and the new farmgate revenues received after market adjustments had produced a new steady state; therefore, a simple accounting approach provides only one perspective on value. It may be useful to think of value as used herein as an historical accounting of the additional gross revenues that have accrued to growers as a result of their having used honey bees, *caeteris paribus*.

A reduction in the availability of pollinators and pollinator dependent crops may have other consequences that are difficult to value. While a change in pollinator availability may lead to market adjustments involving changes in grower production and consumer consumption patterns, all such patterns are not equivalent. Assuming that current patterns without pollinator shortages reflect consumer preferences, changes in those patterns imposed by a loss of pollinators would necessarily reflect less desirable choices. Additionally, while the majority of calories are derived from crops that do not require animal pollination [29], [145], the elimination of crops that do require animal pollination would result in a diet that is culturally impoverished and nutritionally inadequate due to a loss of micronutrients [51], [146].

### Non-*Apis* options for growers

One option available to growers in the event of a sustained loss of honey bees would be to use other pollinators. Non-*Apis* bees, both managed and wild, have great potential as commercial pollinators. Some are more efficient than honey bees on certain crops [145]; management systems for a few are well developed; and protocols for the development of systems for additional species have been proposed [147], [148]. The horned-faced bee, *Osmia cornifrons*, was introduced to the US in 1977 from Japan [149] where it has been successfully used for apple pollination [150], [151]. The blue orchard bee, *O. lignaria*, is useful on a variety of crops including almonds and cherries [147], [152], [153]. Management systems for both are well-developed; however, as with the honey bee, each has its own suite of pests, pathogens, predators and parasites. Scaling production to levels sufficient to replace honey bees on selected crops will take time, and difficulties may arise along the way.

Bumble bees are excellent generalist pollinators and are available commercially. Bumble bees forage at lower temperatures [154] and provide superior pollination on a bee-for-bee basis for some crops, including blueberries and cranberries [130]; however, they are expensive compared to honey bees (approximately 1.00–2.00 USD per bumble bee versus 0.01–0.02 USD per honey bee). As with other non-*Apis* managed pollinators, supply questions remain unanswered.

If production of alfalfa leafcutter bees could be increased, they may increase their contribution to alfalfa seed production and possibly other crops [155]–[157]. However, leafcutter bee production is hampered by a number of parasites and pathogens, production is difficult to sustain in the US [158], [159] and reserve capacity in Canada, the primary source of leafcutter bees for US alfalfa seed growers, is not known. The other commercial alfalfa seed pollinator, *N. melanderi*, requires conditions that would be expensive to duplicate outside of the Pacific Northwest.

If losses extended to other insect pollinators, grower options are very limited. A recent study valued insect pollination for deciduous fruit tree crops in South Africa as equal to the change in net income that growers would receive if insect pollinators were replaced by other means - the replacement cost method [160]. Substituting pollen dusting and hand pollination for insect pollinators was found to be effective, albeit more expensive. Replacement costs using these methods are sensitive to crop values and local labor rates, making them more or less attractive for different cropping systems and different countries. In addition, it may not be possible to collect and distribute pollen from some crops in the manner used for deciduous fruit trees.

Clearly, markets would adjust to a loss of honey bees and other insect pollinators; however, the above discussion suggests that the nature of those adjustments and the time-scale over which they would occur are difficult to predict and would vary from crop to crop. The use of managed non-*Apis* pollinators may be possible for some crops but not for others; and where such use is possible, it may take considerable time to develop reliable, cost-effective management systems and sufficient populations. Further, there is no guarantee that the new equilibrium would include either the same diversity and abundance of insect-pollinated crops or the same level of affordability for those products. In brief, marketplace options for pollinators are simply not equivalent to grower options for most other inputs or most commodities in general. Hence, a precipitous loss of pollinators would likely have a major impact on production and prices, at least in the near term, with crops grown in large monocultures most seriously affected [161].

The concern over the sustainable production of insect-pollinated crops arises in part from the fact that the total number of colonies in the US has trended downward since 1947 [52]. This trend has continued in recent years. The number of colonies declined from 3.53 million in 1989 (five years after detection of the tracheal mite *A. woodi* in the US [162] and two years after detection of *V. destructor* [163]) to 2.30 million in 2008, a decline of 34.81% (Fig. S1 in Text S4); however, there were increases to 2.46 and 2.68 million colonies in 2009 and 2010, respectively. Despite those increases, the overall trajectory maintains a downward trend; and the numbers are already well below the number required to satisfy estimated number of recommended colony rentals (8.98 million in 2009 not including colonies for cotton lint, and 30.40 million including colonies for cotton lint (see Fig. S2 and Text S4 for discussion of underestimates of the contributions of wild bees). Interestingly, the long-term downward trend was underway well before the arrival of parasitic mites CCD. This suggests that the downward trend may be independent of recent, large losses being reported with the primary impact of those losses being an increase in operating costs for beekeepers and pollination rental fees [164]–[169].

Regardless of the cause, the decline in colony numbers does not yet appear to have reduced the production or yield of insect-pollinated crops. The cultivated area of DD crops increased from 1992 through 2004, declining slightly thereafter (Fig. S3 and Text S4). That might suggest a response by growers to maintain production in the face of a decline in the honey bee population [58], [64]; however, other data do not support that hypothesis. The production of DD crops actually increased between 1992 and 2003, after which there was a slight downward trend (Fig. S4 and Text S4). The most rapid growth occurred as the number of colonies declined most rapidly. Additionally, the aggregate yield of DD crops remained steady from 1992 through 2009 despite a declining number of colonies (Fig. S5 and Text S4). These findings suggest that the decline of managed honey bee colonies has not yet resulted in a pollinator shortage. However, aggregate data mask variation among crops; and shortages may disproportionately affect crops with differing degrees of dependency on insect pollinators [64]; therefore, this conclusion should be considered tentative pending further analysis.

Honey bees provide the major share of crop pollination in the US, especially in large cropping systems. There are several reasons for this. Honey bees are an established commodity that fit into a familiar business model in which producers purchase inputs rather than relying on natural ecosystem services [170]. In addition, each colony provides thousands of pollinators; colony management is well developed, so numbers have been adequate and reliable; honey bees are available any time crops are in bloom; honey bees pollinate a large number of crops; honey bees have extended foraging ranges making them suitable for large monocultures; foragers exhibit floral constancy on any single trip to the field; and colonies are easily transported by truck.

While those same factors support a continuing and prominent role for honey bees, the increase in colony rental fees and concerns over possible shortages have provided growers with considerable impetus to diversify their pollinator portfolio. Many growers are experimenting with bumble bees; interest in protecting and enhancing populations of native bees has increased; and recently, one major almond grower established a program to develop a population of several million *O. lignaria*. From a systems perspective, pollinator diversification is highly desirable because it provides redundancy in a critical component of all pollinator-dependent cropping systems, thereby increasing system reliability. To maintain its competitive position, the beekeeping industry will need to develop a sustainable, market-based system of bee breeding and colony management that can continue to provide an adequate and reliable supply of high quality, healthy pollinators at competitive prices.

## Supporting Information

Text S1 **Alfalfa production: s**upporting text for “Insect pollinated crops, insect pollinators and US agriculture: Trend analysis of aggregate data for the period 1992–2009.”
(PDF)

Text S2 **Individual crops for 2002 and 2007: s**upporting text for “Insect pollinated crops, insect pollinators and US agriculture: Trend analysis of aggregate data for the period 1992–2009.”
(PDF)

Text S3 **Update for individual crops for 2010: s**upporting text for “Insect pollinated crops, insect pollinators and US agriculture: Trend analysis of aggregate data for the period 1992–2009.”
(PDF)

Text S4 **Decline in number of honey bee colonies and the pollinator shortage: s**upporting text for “Insect pollinated crops, insect pollinators and US agriculture: Trend analysis of aggregate data for the period 1992–2009.”
(PDF)

Figure S1 **Number of managed colonies of honey bees in the United States.** Predicted values (blue) include adjustments for serial autocorrelation and are the same as the predicted – structural values (also blue) based solely on the structural elements of the model.
(TIF)

Figure S2 **Number of managed colonies required to meet current recommendations for pollination.** Data includes recommendations for all crops except cotton lint. Predicted values (blue) include adjustments for serial autocorrelation and are the same as the predicted – structural values (also blue) based solely on the structural elements of the model.
(TIF)

Figure S3 **Predicted values for the number of managed colonies and hectares of directly dependent crops.** DD = directly dependent.
(TIF)

Figure S4 **Predicted values for the number of managed colonies and tonnes of directly dependent crops.** DD = directly dependent.
(TIF)

Figure S5 **Predicted values for the number of managed colonies and yield of directly dependent crops.** DD = directly dependent.
(TIF)
