# Supplementary material for: Insect Pollinated Crops, Insect Pollinators and US Agriculture: Trend Analysis of Aggregate Data for the Period 1992–2009
Source: PLoS One. 2012 May 22;7(5):e37235. doi: 10.1371/journal.pone.0037235 (PMC3358326; doi:10.1371/journal.pone.0037235)
Supplement: Text S1 — Alfalfa production: supporting text for “Insect pollinated crops, insect pollinators and US agriculture: Trend analysis of aggregate data for the period 1992–2009.” (PDF) [file pone.0037235.s001.pdf]

Supporting Text (S1) for:
Insect pollinated crops, insect pollinators and US agriculture: Trend analysis of aggregate data for the period 1992 – 2009

Alfalfa production

Nicholas W. Calderone
Dyce Laboratory for Honey Bee Studies
Department of Entomology
Cornell University
Ithaca, New York USA

This file includes:

Introduction
Materials and Methods
Results
References
Supplemental Tables S1-S3

Introduction

In the US, alfalfa seed is produced in three general regions: California (CA), the Pacific Northwest (PNW: ID, MT, NV, OR, UT, WA and WY) and other alfalfa-seed producing states (OTHER: AZ, IA, IN, KS, MI, MN, MO, NE, ND, NM, NY, OK, SD and TX). Alfalfa seed is required for the production of alfalfa hay. Alfalfa seed is produced using honey bees, alfalfa leafcutter bees (*Megachile rotundata*), alkali bees (*Nomia melanderi*) and other wild solitary bees and insects with each pollinator playing a greater or lesser role in each region. The apportionment of direct and indirect production (and value) of alfalfa seed and alfalfa hay, respectively, among those pollinators requires estimates of the relative contribution each region makes to total US seed production and the relative contribution each pollinator makes to alfalfa seed production in each region.

Materials and Methods

Data on alfalfa seed production were obtained from the National Agricultural Statistics Service (NASS) Census of Agriculture for 1997 and 2002 [1,2]. Estimates for alfalfa seed production for the years 1992, 1997, 2002 and 2007 are given in Table S1 and Table S2. The total contributions of each region to total US alfalfa seed in those years were relatively constant; therefore, I used the production weighted means for each region as an estimate of the contribution of each region to total US seed production for all years. Weighted four-year means were 0.64795 for the PNW, 0.31122 for CA, and 0.04083 for OTHER states. Prior to 1990, honey bees were the primary pollinator of alfalfa seed in CA, while the leafcutter bee and, to a lesser extent, the alkali bee, were used in the PNW. Growers in OTHER states do not manage alkali bees, and they may or

may not rent honey bees. I assume that half of the seed production in OTHER states is due to honey bees and half to other insects, primarily bumblebees and solitary bees.

The estimate of the proportion of US seed dependent on honey bees in 1988 is calculated as the sum of 0.31122 (the weighted average CA seed production as a proportion of total US seed production) plus 0.02042 (half of the weighted average of alfalfa seed production in OTHER states). An estimate of 0.05 for the dependence of US alfalfa seed on *N. melanderi* is based on published estimates [3], and this is combined with an estimate of 0.02042 (the other half of the weighted average of alfalfa seed production in OTHER states) to give 0.07042, the total proportion due to bumble bees and solitary bees, exclusive of *M. rotundata*. The proportion attributed to *M. rotundata* was calculated as total production less the proportions due to honey bees, bumble bees, alkali bees and other insect pollinators.

Starting around 1990, managed leafcutter bees have been used for alfalfa seed production in increasing numbers in CA; although, they are generally used to compliment honey bees rather than to replace them [4]. Hence, the relative contributions of those two species to the production of alfalfa seed and alfalfa hay have changed over time, with a growing portion due to *M. rotundata*. Based on published reports [4,5], and on an admitted scarcity of hard data, I estimate that the relative contribution of honey bees in CA has decreased by 1% each year since 1990. Estimates of the relative contributions of each pollinator to alfalfa seed production are given in Table S3. These revised estimates should be considered tentative and will require refinement as additional data become available.

References

1. NASS (1999) 1997 Census of Agriculture, ac97-a-51. National Agricultural Statistics Service, USDA. Washington, D.C. 629 p. USDA Census of Agriculture website. Available: http://www.agcensus.usda.gov/Publications/1997/Vol_1_Chapter_1_U._S._National_Level_Data/. Accessed: 4/21/2012.
2. NASS (2004) 2002 Census of Agriculture ac-02-a-51. National Agricultural Statistics Service, USDA. Washington, D.C. 663 p. USDA Census of Agriculture website. Available: http://www.agcensus.usda.gov/Publications/2002/USVolume104.pdf. Accessed: 8/11/2011.
3. Losey J, Vaughan M (2006) The economic value of ecological services provided by insects. Bioscience 56: 311-323.
4. Mueller SC (2008) Alfalfa seed production in California. In: Summers C, Putnam D, editors. Irrigated alfalfa management for mediterranean and desert zones. Pub. Number 8308. . Oakland: University of California Agriculture and Natural Resources Communication Services.
5. Anon (1995) Use of the alfalfa leafcutter bee growing in California. Seed Trade News 116: 14.

Table S1. Alfalfa seed production in 1992 and 1997.

**1997**				**1992**			
**State**	**Production (lbs)**	**Production (kgs)**	**Proportion US Total**	**State**	**Production (lbs)**	**Production (kgs)**	**Proportion US Total**
UT	1,234,694	560,048	0.01476	UT	1,046,414.00	474,645	0.01440
WA	10,990,656	4,985,278	0.13142	WA	13,564,621.00	6,152,809	0.18660
WY	2,010,084	911,759	0.02404	WY	864,720.00	392,230	0.01190
OR	6,524,648	2,959,531	0.07802	OR	703,437.00	319,074	0.00968
NV	7,636,279	3,463,758	0.09131	NV	5,245,571.00	2,379,351	0.07216
MT	3,442,626	1,561,549	0.04117	MT	2,466,195.00	1,118,647	0.03393
ID	22,819,512	10,350,757	0.27287	ID	21,695,511.00	9,840,918	0.29846
**PNW sub-total**	**54,658,499**	**24,792,678**	**0.65360**	**PNW sub-total**	**45,586,469.00**	**20,677,675**	**0.62711**
**CA sub-total**	**26,329,096**	**11,942,677**	**0.31484**	**CA sub-total**	**25,082,048.00**	**11,377,026**	**0.34504**
AZ	735,702	333,709	0.00880	AZ	299,757.00	135,967	0.00412
CO	311,599	141,339	0.00373	CO	118,513.00	53,757	0.00163
IA	29,750	13,494	0.00036	IA	11,840.00	5,371	0.00016
IN	5,720	2,595	0.00007	IN	4,720.00	na	0.00006
KS	294,269	133,478	0.00352	KS	134,970.00	61,221	0.00186
MI	2,680	1,216	0.00003	MI	na	na	na
MN	39,440	17,890	0.00047	MN	144,730.00	65,648	0.00199
MO	na	na	na	MO	na	na	na
NE	78,615	35,659	0.00094	NE	184,285.00	83,590	0.00254
ND	36,765	16,676	0.00044	ND	99,026.00	44,917	0.00136
NM	112,843	51,185	0.00135	NM	245,787.00	111,487	0.00338
NY	na	na	na	NY	na	na	na
OH	15,660	7,103	0.00019	OH	5,850.00	2,654	0.00008
OK	158,143	71,732	0.00189	OK	152,804.00	69,311	0.00210
PA	9,480	na	0.00011	PA	6,545.00	(na)	0.00009
SD	614,546	278,753	0.00735	SD	400,075.00	181,471	0.00550
TX	156,760	71,105	0.00187	TX	214,980.00	97,513	0.00296
Not specified	37,556	17,035	0.00045	-	-	-	-
**OTHER sub-total**	**2,639,528**	**1,192,970**	**0.03156**	**OTHER sub-total**	**2,023,882**	**912,908**	**0.02784**
**Totals**	**83,627,123**	**37,928,325**	**∑=1.00**	**Totals**	**72,692,399**	**32,967,608**	**∑=1.00**

CA = California; PNW = Pacific Northwest (UT, WA, WY, OR, NV, MT and ID); OTHER = other alfalfa seed producing states (source: 1997 Census of Agriculture Table 28); na = not available; lbs = pounds; kgs = kilograms

Table S2. Alfalfa seed production in 2002 and 2007.

**2007**				**2002**			
**State**	**Production (lbs)**	**Production (kgs)**	**Proportion US Total**	**State**	**Production (lbs)**	**Production (kgs)**	**Proportion US Total**
UT	2,077,813	942,480	0.03345	UT	830,889	376,885	0.01432
WA	10,860,608	4,926,289	0.17485	WA	11,887,387	5,392,028	0.20488
WY	5,915,819	2,683,370	0.09524	WY	2,400,315	1,088,765	0.04137
OR	3,183,375	1,443,955	0.05125	OR	3,783,887	1,716,342	0.06522
NV	4,237,101	1,921,917	0.06821	NV	4,695,737	2,129,950	0.08093
MT	3,729,635	1,691,734	0.06004	MT	2,024,033	918,086	0.03489
ID	9,346,709	4,239,596	0.15047	ID	13,910,135	6,309,531	0.23975
**PNW sub-total**	**39,351,060**	**17,849,341**	**0.63352**	**PNW sub-total**	**39,532,383**	**17,931,587**	**0.68136**
**CA sub-total**	**19,083,458**	**8,656,111**	**0.30723**	**CA sub-total**	**15,543,144**	**7,050,252**	**0.26789**
AZ	1,902,669	863,036	0.03063	AZ	574,020	260,371	0.00989
IN	na	na	na	CO	58,293	26,441	0.00100
KS	22,430	10,174	0.00036	IA	12,467	5,655	0.00021
MI	15,610	7,081	0.00025	IN	na	na	na
MN	63,461	28,785	0.00102	KS	1,133,502	514,148	0.01954
MO	40,540	18,389	0.00065	MI	8,870	4,023	0.00015
NE	21,216	9,623	0.00034	MN	59,245	26,873	0.00102
ND	34,784	15,778	0.00056	MO	29,709	13,476	0.00051
NM	29,907	13,566	0.00048	NE	109,428	49,636	0.00189
NY	6,180	2,803	0.00010	ND	38,482	17,455	0.00066
OK	281,121	127,514	0.00453	NM	75,036	34,036	0.00129
PA	na	na	na	NY	101,827	46,188	0.00176
SD	428,447	194,340	0.00690	OH	2,520	1,143	0.00004
TX	79,885	36,235	0.00129	OK	335,878	152,352	0.00579
CO, IA, OH	754,471	342,222	0.01215	PA	na	na	na
-	-	-	-	SD	327,576	148,586	0.00565
-	-	-	-	TX	77,600	35,199	0.00134
**OTHER sub-total**	**3,680,721**	**1,669,547**	**0.05926**	**OTHER sub-total**	**2,944,453**	**1,335,581**	**0.05075**
**Totals**	**62,115,239**	**28,174,998**	**∑=1.00**	**Totals**	**58,019,980**	**26,317,420**	**∑=1.00**

CA = California; PNW = Pacific Northwest (UT, WA, WY, OR, NV, MT and ID); OTHER = other alfalfa seed producing states (source: 1997 Census of Agriculture Table 27); na = not available; lbs = pounds; kgs = kilograms

Table S3. Proportion of California (CA) and US alfalfa seed crop attributed to *A. mellifera, M. rotundata, N. melanderi* and other pollinating insects.

**YEAR**	**Proportion of CA seed dependent on *A. mellifera***	**Proportion of US seed dependent on *A. mellifera***	**Proportion of US seed dependent on *M. rotundata***	**Proportion of US seed dependent on *N. melanderi***	**Proportion of US seed dependent on *N. melanderi* and other insect pollinators**
**1988**	1.0000	0.3316	0.5979	0.0500	0.0704
**1989**	1.0000	0.3316	0.5979	0.0500	0.0704
**1990**	1.0000	0.3316	0.5979	0.0500	0.0704
**1991**	0.9900	0.3285	0.6011	0.0500	0.0704
**1992**	0.9800	0.3254	0.6042	0.0500	0.0704
**1993**	0.9700	0.3223	0.6073	0.0500	0.0704
**1994**	0.9600	0.3192	0.6104	0.0500	0.0704
**1995**	0.9500	0.3161	0.6135	0.0500	0.0704
**1996**	0.9400	0.3130	0.6166	0.0500	0.0704
**1997**	0.9300	0.3099	0.6197	0.0500	0.0704
**1998**	0.9200	0.3067	0.6229	0.0500	0.0704
**1999**	0.9100	0.3036	0.6260	0.0500	0.0704
**2000**	0.9000	0.3005	0.6291	0.0500	0.0704
**2001**	0.8900	0.2974	0.6322	0.0500	0.0704
**2002**	0.8800	0.2943	0.6353	0.0500	0.0704
**2003**	0.8700	0.2912	0.6384	0.0500	0.0704
**2004**	0.8600	0.2881	0.6415	0.0500	0.0704
**2005**	0.8500	0.2850	0.6446	0.0500	0.0704
**2006**	0.8400	0.2818	0.6477	0.0500	0.0704
**2007**	0.8300	0.2787	0.6509	0.0500	0.0704
**2008**	0.8200	0.2756	0.6540	0.0500	0.0704
**2009**	0.8100	0.2725	0.6571	0.0500	0.0704

The estimate of the proportion of US seed dependent on honey bees is calculated as the sum of 0.3112 (weighted average CA seed production as a % of total US seed production based on 1992, 1997, 2002 and 2007) plus 0.0204 (half of the weighted average of alfalfa seed production in OTHER states from Table 5). An estimate of 0.05 for the dependence of US alfalfa seed on *N. melanderi* is based on Losey and Vaughan (2006), and this is combined with an estimate of 0.0204 (the other half of the weighted average of alfalfa seed production in OTHER states from Table 5) to give 0.0704, the estimate of the proportion of seed due to insects other than honey bees and leafcutter bees. Value for the proportion of production attributed to *M. rotundata* was calculated as total production less the proportions due to honey bees and other insects. Based on conversations with CA field crop specialist, I assumed that the increase in the use of *M. rotundata* starting in 1990 results in a decrease in reliance on honey bees of 1% per year.
