# Supplementary material for: Insect Pollinated Crops, Insect Pollinators and US Agriculture: Trend Analysis of Aggregate Data for the Period 1992–2009
Source: PLoS One. 2012 May 22;7(5):e37235. doi: 10.1371/journal.pone.0037235 (PMC3358326; doi:10.1371/journal.pone.0037235)
Supplement: Text S2 — Individual crops for 2002 and 2007: supporting text for “Insect pollinated crops, insect pollinators and US agriculture: Trend analysis of aggregate data for the period 1992–2009.” (PDF) [file pone.0037235.s002.pdf]

Supporting Text (S2) for:

Insect pollinated crops, insect pollinators and US agriculture: Trend analysis of aggregate data for the period 1992 – 2009

Individual crops for 2002 and 2007

Nicholas W. Calderone
Department of Entomology
Cornell University
Ithaca, New York USA

This file includes:

Introduction
Materials and Methods
Results
References
Supplemental Tables S4-S11

Introduction

Aggregate data mask the contributions of individual crops to the trends reported in the originating manuscript. To provide the reader with a perspective on the contributions of individual crops, data for individual crops for 2002 and 2007 are reported here. Those years were selected because they are the most recent for which NASS Final Estimates and COA data were available. Using COA data allowed for the inclusion of data for crops not available on an annual basis (alfalfa and non-alfalfa legume seed production, pumpkins and squash) and makes values for most metrics slightly higher than corresponding values for those years reported in the trends in the originating manuscript.

Materials and Methods

Statistics for individual crops for 2002 and 2007

Data for individual crops for both directly dependent crops (DD Crops: e.g. apples, almonds, cherries, oranges, squash, vegetable and legume seeds, etc.), and indirectly dependent crops (ID Crops: including field crops (legume hay, sugar beets, etc.) and vegetables (asparagus, broccoli, carrots, onions, etc.)) were obtained from the National Agricultural Statics Service (NASS) Census of Agriculture (COA) for 2002 and 2007 [1,2]. Those data are reported in Table S4-S9.

Non-alfalfa legume seed production**:** Since annual data for non-alfalfa legume seed and hay production and values are not available in the NASS Annual Reports or Final Estimates, they are not included in any trend analysis. However, production data are available in the 2002 and 2007 COA reports [1,2], and some market value data are available for CA, MT and WA alfalfa seed production in state agricultural reports (Tables S10-S11). To estimate the value of those crops in

those two years, a nominal value of $1.00 per pound was assigned to each of the legume seeds. Data for all seeds were combined and presented as non-alfalfa legume seed in the statistics for 2002 and 2007.

Results

***Production*:** Total production of DD Crops was 119.67 million tonnes in 2002 (Tables S4 and S5) compared to 113.05 million tonnes in 2007 (Tables S6 and S7). Total production of ID Crops was 101.88 million tonnes in 2002 (Table S8) compared to 103.66 million tonnes in 2007 (Table S9).

***Cultivated acres*:** The number of cultivated hectares of DD Crops was 32.54 million in 2002 (Tables S4 and S5) compared to 29.41 million in 2007 (Tables S6 and S7). The reduction in cultivated hectares in 2007 was out of line with the general trend due to a reduction in hectares across a wide range of crops, most notably a decline in hectares of soybeans. The number of cultivated hectares of ID Crops was 15.07 million in 2002 (Table S8) compared to 13.50 million in 2007 (Table S9).

***Total value (2009 USD)*:** The total value of DD Crops was $40.71 B in 2002 (Tables S4 and S5) compared to $55.78 B in 2007 (Tables S6 and S7), an increase of 37.02 %. The corresponding values for ID Crops were $17.26 B (Table S8) and $19.31 B (Table S9), an increase of 11.88 %.

***Value attributed to insect pollination (2009 USD)*:** The value of DD Crops attributed to insect pollination was $12.26 B in 2002 (Tables S4 and S5) compared to $16.45 B in 2007 (Tables 6

and 7), an increase of 34.18 %. The value of ID Crops attributed to insect pollination was $12.48 B in 2002 (Table 8) compared to $13.40 B in 2007 (Table 9), an increase of 7.37 %.

Value attributed to honey bee pollination (2009 USD)**:** The value of DD Crops attributed to honey bees was $9.39 B in 2002 (Tables S4 and S5) compared to $12.78 B in 2007 (Tables S6 and S7), an increase of 36.10 %. The value of ID Crops attributed to honey bees was $5.87 B in 2002 (Table S8) compared to $6.10 B in 2007 (Table S9), an increase of 3.92 %.

Value attributed to M. rotundata (2009 USD)**:** The value of DD Crops attributed to *M. rotundata* (alfalfa seed) was $43.96 million in 2002 and $48.13 million in 2007 (Tables S5 and S7, respectively), an increase of 9.49 %. The value of ID Crops attributed to *M. rotundata* (alfalfa hay) was $5.41 B in 2002 (Table S8) and $5.96 B in 2007 (Table S9), an increase of 10.17 %.

Value attributed to non-Apis insect pollinators (2009 USD)**:** The value of DD Crops attributed to non-*Apis* insect pollinators (other than *M. rotundata*) was $2.82 B in 2002 (Tables S4 and S5) compared to $3.63 B in 2007 (Tables S6 and S7), an increase of 28.72 %. The value of ID Crops attributed to non-*Apis* insect pollinators was $1.21 B in 2002 (Table S8) compared to $1.32 B in 2007 (Table S9), an increase of 9.09 %.

Production and cultivated area of non-alfalfa legume seeds by state (2009 USD): Statistics on the production and cultivated area of non-alfalfa legume seeds are given in Tables S10 and S11.

References

1. NASS (2009) 2007 Census of Agriculture ac-07-a-51. National Agricultural Statistics Service, USDA. Washington, D.C. 739 p. USDA NASS Agriculture Census website. Available: http://www.agcensus.usda.gov/Publications/2007/Full_Report/usv1.pdf. Accessed: 8/11/2011.

2. NASS (2004) 2002 Census of Agriculture ac-02-a-51. National Agricultural Statistics Service, USDA. Washington, D.C. 663 p. USDA NASS Agriculture Census website. Available: http://www.agcensus.usda.gov/Publications/2002/USVolume104.pdf. Accessed: 8/11/2011.

Table S4. Production, cultivated area and value data[1] for crops requiring or benefiting from pollination in 2002 – PART I.

Commodity	Hectares (1,000's)	Production (1,000's tonnes)	Total value	Total value due to insect pollinators	Total value due to honey bees	Total value due to *M. rotundata*	Total value due to other insect pollinators
**BERRIES**							
blackberry	2.63	21.41	24,669.97	19,735.97	17,762.38	0.00	1,973.60
blueberry [cultivated]	16.94	87.09	232,026.71	232,026.71	208,824.04	0.00	23,202.67
blueberry [wild]	9.31	28.30	21,298.67	21,298.67	19,168.80	0.00	2,129.87
boysenberries	0.45	2.27	3,959.22	3,167.37	2,850.64	0.00	316.74
cranberry	15.94	258.09	217,975.08	217,975.08	196,177.57	0.00	21,797.51
loganberries	0.03	0.09	250.43	125.22	100.17	0.00	25.04
raspberry [all (CA)]	0.97	13.24	48,716.24	38,972.99	35,075.69	0.00	3,897.30
raspberry [black (OR)]	0.49	1.36	1,420.31	1,136.25	1,022.62	0.00	113.62
raspberry [red]	4.73	37.10	50,730.43	40,584.34	36,525.91	0.00	4,058.43
strawberry	19.26	854.79	1,385,284.13	277,056.83	27,705.68	0.00	249,351.14
**CITRUS**							
grapefruit	55.16	2,199.02	348,406.18	278,724.94	250,852.45	0.00	27,872.49
lemon	26.63	726.65	391,108.46	78,221.69	7,822.17	0.00	
lime	0.32	6.35	2,065.47	619.64	557.68	0.00	61.96
orange	322.78	11,225.50	2,201,656.45	660,496.93	594,447.24	0.00	66,049.69
tangelo	3.93	88.00	12,829.29	5,131.72	4,618.54	0.00	513.17
tangerine	15.70	381.02	148,730.55	74,365.27	66,928.75	0.00	7,436.53
temple	1.90	63.50	8,251.15	2,475.34	2,227.81	0.00	247.53
**CUCURBITS**							
cucumber [fresh]	22.22	496.18	247,789.64	223,010.67	200,709.61	0.00	22,301.07
cucumber [pickled]	47.67	561.83	201,545.53	181,390.97	163,251.88	0.00	18,139.10
muskmelon [cantaloupe]	36.34	1,018.00	474,988.97	379,991.18	341,992.06	0.00	37,999.12
muskmelon [honeydew]	9.87	229.74	109,060.88	87,248.70	78,523.83	0.00	8,724.87
pumpkin	16.59	385.96	109,369.75	98,432.77	9,843.28	0.00	88,589.49
squash	21.17	398.80	242,054.74	217,849.27	21,784.93	0.00	196,064.34
watermelon	61.71	1,795.55	391,744.08	274,220.86	246,798.77	0.00	27,422.09
**GRAPES**							
grapes	384.43	6,657.74	3,388,669.75	338,866.98	33,886.70	0.00	304,980.28

[1] All value data in 2009 USD

Table S5. Production, cultivated area and value data[1] for crops requiring or benefiting from pollination in 2002 – PART II.

Commodity	Hectares (1,000's)	Production (1,000's tonnes)	Total value	Total value due to insect pollinators	Total value due to honey bees	Total value due to *M. rotundata*	Total value due to other insect pollinators
**LEGUMES**							
peanut	na	1,506.40	715,179.78	71,517.98	14,303.60	0.00	57,214.38
soybean	29,338.49	75,010.03	18,189,363.92	1,818,936.39	909,468.20	0.00	909,468.20
**SEEDS**							
alfalfa [seed]	44.77	26.32	69,191.41	69,191.41	20,362.25	43,956.93	4,872.23
almond	220.55	800.05	1,431,860.96	1,431,860.96	1,431,860.96	0.00	0.00
canola	518.40	695.55	194,048.06	97,024.03	87,321.63	0.00	9,702.40
cotton [seed]	na	5,609.94	737,645.55	147,529.11	117,603.39	0.00	29,400.85
macademia	7.20	24.04	36,026.47	32,423.83	29,181.44	0.00	3,242.38
non-alfalfa legume seed	32.75	8.64	22,711.14	22,711.14	20,440.03	0.00	2,271.11
rapeseed	1.25	1.82	465.09	465.09	418.58	0.00	46.51
sunflower	876.95	1,111.87	351,314.77	351,314.77	316,183.29	0.00	35,131.48
**TREE FRUITS**							
apple	159.77	3,866.42	1,885,707.49	1,885,707.49	1,697,136.74	0.00	188,570.75
apricot	7.02	81.65	34,064.75	23,845.33	19,076.26	0.00	4,769.07
avocado	26.57	180.89	455,772.47	455,772.47	410,195.22	0.00	45,577.25
cherry [sweet]	29.43	164.56	327,316.20	294,584.58	265,126.12	0.00	29,458.46
cherry [tart]	15.26	28.39	33,246.68	29,922.01	26,929.81	0.00	2,992.20
kiwifruit	1.82	23.68	21,581.30	19,423.17	17,480.85	0.00	1,942.32
nectarine	14.77	272.16	136,664.48	81,998.69	65,598.95	0.00	16,399.74
olive	14.57	93.44	70,339.28	7,033.93	703.39	0.00	6,330.53
peach	59.23	1,149.86	581,970.07	349,182.04	279,345.63	0.00	69,836.41
pear	25.95	807.39	315,227.48	220,659.23	198,593.31	0.00	22,065.92
plum	14.57	182.34	92,524.00	64,766.80	58,290.12	0.00	6,476.68
prune	29.95	471.01	157,450.36	110,215.25	99,193.73	0.00	11,021.53
prune and plum	1.62	14.24	5,052.77	3,536.94	3,183.24	0.00	353.69
**COTTON**							
cotton [lint]	sid	sid	4,578,245.11	915,649.02	732,519.22	0.00	183,129.80
**2002 Totals**	**32,538.06**	**119,668.28**	**40,707,571.67**	**12,258,398.04**	**9,389,975.16**	**43,956.93**	**2,823,941.07**

[1]All value data in 2009 USD; na = not available; sid = see indirectly dependent crops Table S8

Table S6. Production, cultivated area and value data[1] for crops requiring or benefiting from pollination in 2007 – PART I.

Commodity	Hectares (1,000's)	Production (1,000's tonnes)	Total value	Total value due to insect pollinators	Total value due to honey bees	Total value due to *M. rotundata*	Total value due to other insect pollinators
**BERRIES**							
blackberry	2.71	29.03	29,589.35	23,671.48	21,304.33	0.00	2,367.15
blueberry [cultivated]	21.62	130.27	549,503.90	549,503.90	494,553.51	0.00	54,950.39
blueberry [wild]	9.31	35.02	85,912.27	85,912.27	77,321.04	0.00	8,591.23
boysenberries	0.33	2.54	3,156.87	2,525.50	2,272.95	0.00	252.55
cranberry	15.42	297.28	317,894.33	317,894.33	286,104.90	0.00	31,789.43
loganberries	0.02	0.04	91.05	45.53	36.42	0.00	9.11
raspberry [all (CA)]	1.42	35.74	234,670.21	187,736.17	168,962.55	0.00	18,773.62
raspberry [black (OR)]	0.57	1.72	3,707.33	2,965.87	2,669.28	0.00	296.59
raspberry [red]	4.53	27.31	33,441.54	26,753.23	24,077.91	0.00	2,675.32
strawberry	21.12	1,109.17	1,811,873.41	362,374.68	36,237.47	0.00	326,137.21
**CITRUS**							
grapefruit	34.84	1,475.99	322,737.77	258,190.21	232,371.19	0.00	25,819.02
lemon	24.48	723.93	465,012.27	93,002.45	9,300.25	0.00	83,702.21
orange	274.09	6,917.28	2,293,385.03	688,015.51	619,213.96	0.00	68,801.55
tangelo	2.23	50.80	14,232.31	5,692.93	5,123.63	0.00	569.29
tangerine [and mandarins]	14.89	327.49	161,618.25	80,809.12	72,728.21	0.00	8,080.91
**CUCURBITS**							
cucumber [fresh]	20.62	439.98	247,215.97	222,494.37	200,244.93	0.00	22,249.44
cucumber [pickled]	41.08	491.00	181,923.22	163,730.90	147,357.81	0.00	16,373.09
muskmelon [cantaloupe]	29.87	926.51	312,981.57	250,385.26	225,346.73	0.00	25,038.53
muskmelon [honeydew]	7.10	187.97	76,068.12	60,854.50	54,769.05	0.00	6,085.45
pumpkin	18.58	519.73	127,805.25	115,024.72	11,502.47	0.00	103,522.25
squash	16.83	284.22	179,952.12	161,956.90	16,195.69	0.00	145,761.21
watermelon	52.20	1,694.12	437,208.82	306,046.17	275,441.56	0.00	30,604.62
**GRAPES**							
grapes	378.99	6,384.13	3,571,638.24	357,163.82	35,716.38	0.00	321,447.44

[1]All value data in 2009 USD

Table S7. Production, cultivated area and value data[1] for crops requiring or benefiting from pollination in 2007 – Part II.

Commodity	Hectares (1,000's)	Production (1,000's tonnes)	Total value	Total value due to insect pollinators	Total value due to honey bees	Total value due to *M. rotundata*	Total value due to other insect pollinators
**LEGUMES**							
peanut	483.60	1,665.70	784,951.17	78,495.12	15,699.02	0.00	62,796.09
soybean	25,958.97	72,859.19	27,910,448.15	2,791,044.81	1,395,522.41	0.00	1,395,522.41
**SEEDS**							
alfalfa [seed]	49.16	28.17	73,944.78	73,944.78	20,610.47	48,127.37	5,206.95
almond	259.00	1,100.32	2,485,222.76	2,485,222.76	2,485,222.76	0.00	0.00
canola	467.61	648.97	269,373.06	134,686.53	121,217.87	0.00	13,468.65
cotton [seed]	na	5,977.17	1,106,973.96	221,394.79	177,748.22	0.00	44,437.06
macademia	6.07	18.60	25,453.65	22,908.28	20,617.45	0.00	2,290.83
non-alfalfa legume seed	14.11	6.65	15,237.59	15,237.59	13,713.83	0.00	1,523.76
rapeseed	0.45	0.55	221.43	221.43	199.28	0.00	22.14
sunflower	814.23	1,301.30	636,068.03	636,068.03	572,461.23	0.00	63,606.80
**FRUIT TREES**							
apple	142.00	4,122.88	2,698,728.16	2,698,728.16	2,428,855.34	0.00	269,872.82
apricot	5.12	80.29	43,692.32	30,584.63	24,467.70	0.00	6,116.93
avocado	29.68	170.64	339,474.06	339,474.06	305,526.65	0.00	33,947.41
cherry [sweet]	33.05	281.86	576,386.47	518,747.82	466,873.04	0.00	51,874.78
cherry [tart]	14.06	114.85	70,280.00	63,252.00	56,926.80	0.00	6,325.20
kiwifruit	1.70	22.23	23,298.37	20,968.53	18,871.68	0.00	2,096.85
nectarine	13.11	256.73	99,646.89	59,788.13	47,830.51	0.00	11,957.63
olive	12.14	120.20	89,702.38	8,970.24	897.02	0.00	8,073.21
peach	50.71	1,022.58	519,509.98	311,705.99	249,364.79	0.00	62,341.20
pear	23.73	791.97	375,691.70	262,984.19	236,685.77	0.00	26,298.42
plum	11.94	137.89	104,584.49	73,209.14	65,888.23	0.00	7,320.91
prune	25.90	218.45	121,525.65	85,067.95	76,561.16	0.00	8,506.80
prune and plum	1.35	10.98	5,127.98	3,589.59	3,230.63	0.00	358.96
**COTTON**							
cotton [lint]	sid	sid	5,945,010.12	1,189,002.02	951,201.62	0.00	237,800.40
**2007 Totals**	**29,410.53**	**113,049.46**	**55,782,172.34**	**16,448,046.39**	**12,775,045.70**	**48,127.37**	**3,625,663.81**

[1]All value data in 2009 USD; na = not available; sid = see indirectly dependent crops Table S9

Table S8. Production, cultivated area and value data[1] for 2002 for crops grown from seeds that require pollination.

Commodity	Hectares (1,000's)	Production (1,000's tonnes)	Total value	Total value due to insect pollinators	Total value due to honey bees	Total value due to *M. rotundata*	Total value due to other insect pollinators
**FIELD CROPS**							
alfalfa [hay]	9,276.61	66,237.19	8,511,679.75	8,511,679.75	2,504,891.23	5,407,424.16	599,364.36
cotton [lint]	5,024.82	3,746.73	4,504,361.13	900,872.23	720,697.78	0.00	180,174.45
sugarbeet	550.66	25,135.37	1,308,602.96	130,860.30	26,172.06	0.00	104,688.24
**VEGETABLES**							
asparagus	26.71	84.73	206,160.64	206,160.64	185,544.57	0.00	20,616.06
broccoli	52.77	833.48	677,081.87	677,081.87	609,373.69	0.00	67,708.19
Carrot [fresh]	35.01	1,173.22	588,236.84	588,236.84	529,413.16	0.00	58,823.68
carrot [processing]	6.31	364.01	33,505.46	33,505.46	30,154.91	0.00	3,350.55
cauliflower	16.59	282.13	235,606.70	235,606.70	212,046.03	0.00	23,560.67
celery	10.97	849.90	286,024.69	286,024.69	228,819.75	0.00	57,204.94
onion	65.85	3,168.07	912,281.92	912,281.92	821,053.73	0.00	91,228.19
**2002 Totals**	**15,066.29**	**101,874.82**	**17,263,541.96**	**12,482,310.39**	**5,868,166.91**	**5,407,424.16**	**1,206,719.32**

[1]All value data in 2009 USD

Table S9. Production, cultivated area and value data[1] for 2007 for crops grown from seeds that require pollination.

Commodity	Hectares (1,000's)	Production (1,000's tonnes)	Total value	Total value due to insect pollinators	Total value due to honey bees	Total value due to *M. rotundata*	Total value due to other insect pollinators
**FIELD CROPS**							
alfalfa [hay]	8,549.39	63,394.07	9,162,323.96	9,162,323.96	2,553,794.38	5,963,349.01	645,180.57
cotton [lint]	4,244.79	4,181.81	5,849,069.21	1,169,813.84	935,851.07	0.00	233,962.77
sugarbeet	504.56	28,879.32	1,381,016.62	138,101.66	27,620.33	0.00	110,481.33
**VEGETABLES**							
asparagus	15.62	51.03	107,685.48	107,685.48	96,916.94	0.00	10,768.55
broccoli	52.57	870.35	719,036.57	719,036.57	647,132.92	0.00	71,903.66
carrot [fresh]	32.01	1,108.13	559,187.67	559,187.67	503,268.90	0.00	55,918.77
carrot [processing]	6.08	342.14	28,100.41	28,100.41	25,290.37	0.00	2,810.04
cauliflower	15.31	309.71	241,512.69	241,512.69	217,361.42	0.00	24,151.27
celery	11.49	907.68	422,159.09	422,159.09	337,727.27	0.00	84,431.82
onion	64.78	3,612.32	844,379.23	844,379.23	759,941.31	0.00	84,437.92
**2007 Totals**	**13,496.60**	**103,656.57**	**19,314,470.95**	**13,392,300.62**	**6,104,904.92**	**5,963,349.01**	**1,324,046.69**

[1]All value data in 2009 USD

Table S10. Legume seed production for 2002 (source: NASS 2002 Census of Agriculture).

Seed Crop 2002	Primary Pollinator	Farms	US total acres	US total hectares	US total production (lbs)	US total production (kg)	lbs/acre	kgs/acre
Alfalfa – US	*A. mellifera, M. rotundata, N. melanderi*	1,234	110,617.00	44,765.11	58,020,460	26,317,637.96	524.52	587.91
Alfalfa – CA	*A. mellifera, M. rotundata*	153	27,160	10,991.26	15,543,144	7,050,251.52	572.28	641.44
**Alfalfa – CA as % US Total**	**.**	**12.40%**	**24.55%**	**24.55%**	**26.79%**	**26.79%**	**.**	**.**
Birdsfoot trefoil	*A. mellifera*[1]	89	4,676.00	1,892.31	418,343.00	189,757.19	89.47	100.28
Crimson clover	*A. mellifera*[1]	56	3,166.00	1,281.23	2,002,569.00	908,350.02	632.52	708.96
Ladino clover	*A. mellifera*[1]	10	2,049	829.20	938,510	425,700.98	458.03	513.39
Lespedeza	*A. mellifera*[1]	358	23,898	9,671.18	5,413,440	2,455,495.08	226.52	253.90
Red clover	*A. mellifera*[1]	1,240	43,936	17,780.27	9,284,591	4,211,419.64	211.32	236.86
Vetch	*A. mellifera*[1]	69	3,190	1,290.95	956,272	433,757.68	299.77	336.00
Sweet clover	*A. mellifera*	4	na	na	30,700	13,925.29	na	na
White clover	*A. mellifera*[1]	21	na	na	na	na	na	na
**US Totals**		**3,081**	**191,532.00**	**77,510.25**	**77,064,885.00**	**34,956,043.83**	**402.36**	**450.99**

[1]Contribution from other bees not known; lbs = pounds; kgs = kilograms; na = not available

Table S11. Legume seed production for 2007 (source: NASS 2007 Census of Agriculture).

Seed Crop 2007	Primary Pollinator	Farms	US total acres	US total hectares	US total production (lbs)	US total production (kg)	lbs/acre	kgs/hectare
Alfalfa - US	*A. mellifera, M. rotundata, N. melanderi*	806.00	121,467.00	49,155.95	62,115,239.00	28,174,998.47	511.38	573.18
Alfalfa - CA	*A. mellifera, M. rotundata*	114.00	36,625.00	14,821.61	19,083,458.00	8,656,110.94	521.05	584.02
**Alfalfa – CA as % US Total**	**.**	**14.14%**	**30.15%**	**12.20%**	**30.72%**	**30.72%**	**.**	**.**
Birdsfoot trefoil	*A. mellifera*[1]	20.00	1,014.00	410.35	72,825.00	33,032.86	71.82	80.50
Crimson clover	*A. mellifera*[1]	67.00	3,496.00	1,414.78	2,602,578.00	1,180,509.52	744.44	834.41
Ladino clover	*A. mellifera*[1]	2.00	na	na	na	na	na	na
Lespedeza	*A. mellifera*[1]	66.00	4,909.00	1,986.60	1,132,473.00	513,681.11	230.69	258.57
Red clover	*A. mellifera*[1]	434.00	21,387.00	8,655.01	8,213,873.00	3,725,750.12	384.06	430.47
Vetch	*A. mellifera*[1]	61.00	na	na	1,157,122.00	524,861.71	na	na
White clover	*A. mellifera*[1]	32.00	4,059.00	1,642.62	1,472,657.00	667,985.98	362.81	406.66
**US Totals**		**1,488.00**	**156,332.00**	**63,265.32**	**76,766,767.00**	**34,820,819.78**	**491.05**	**550.39**

[1]Contribution from other bees not known; lbs = pounds; kgs = kilograms; na = not available
