# Supplementary material for: Insect Pollinated Crops, Insect Pollinators and US Agriculture: Trend Analysis of Aggregate Data for the Period 1992–2009
Source: PLoS One. 2012 May 22;7(5):e37235. doi: 10.1371/journal.pone.0037235 (PMC3358326; doi:10.1371/journal.pone.0037235)
Supplement: Text S3 — Update for individual crops for 2010: supporting text for “Insect pollinated crops, insect pollinators and US agriculture: Trend analysis of aggregate data for the period 1992–2009.” (PDF) [file pone.0037235.s003.pdf]

Supporting Text (S3) for:

# Insect pollinated crops, insect pollinators and US agriculture: Trend analysis of aggregate data for the period 1992 – 2009

## Update for individual crops for 2010

Nicholas W. Calderone
Department of Entomology
Cornell University
Ithaca, New York USA

This file includes:

Introduction
Materials and Methods
Results
References
Supplementary Tables S12-S14

## Introduction

Insect pollination contributes to US agriculture two ways. First, the production of many crops requires or benefits directly from pollination (directly dependent crops such as apples, almonds, cherries, oranges, squash, vegetable and legume seeds). Second, insect pollination is indirectly responsible for other crops that do not themselves require pollination but that are grown from seeds that do require pollination (indirectly dependent crops such as legume hay, sugar beets, asparagus, broccoli, carrots and onions). To provide a more recent data set, I report values for crop metrics for those two crop groups for 2010.

## Materials and Methods

Data for production, units of production, cultivated area and the value of production for 2010 were obtained from NASS [1-5]. Cultivated acres were converted hectares; production was converted to tonnes; values are given in 2010 USD. Data for legume seed production was not available.

## Results

In 2010, the value of directly dependent crops resulting from insect pollination amounted to $16.35 billion (Tables S12 and S13) while the value of indirectly dependent crops resulting from insect pollination was $12.65 billion (Table S14). Counting both directly and indirectly dependent crops, insect pollination added more than $29 billion (2010 USD) to US agricultural output in 2010. Honey bees added almost $12.4 billion worth of directly dependent crops and $6.8 billion worth of indirectly dependent crops to agricultural output in 2010. Insects other than honey bees added nearly $4.0 billion worth of directly dependent crops and $5.9 billion worth of indirectly dependent crops in 2010.

## References

1. NASS (2011) Noncitrus fruits and nuts 2010 summary (July 2011). National Agricultural Statistics Service, USDA. Washington, D.C. 83 p. Mann Library, USDA-ESMIS website. Available: http://usda01.library.cornell.edu/usda/nass/NoncFruiNu/2010s/2011/NoncFruiNu-07-07-2011.pdf. Accessed: 4/21/2012.
2. NASS (2011) Crop production 2010 summary. National Agricultural Statistics Service, USDA. . Washington, D.C. . 91 p. Mann Library, USDA-ESMIS website. Available: http://usda01.library.cornell.edu/usda/nass/CropProdSu//2010s/2011/CropProdSu-01-12-2011_revision.pdf. Accessed: 4/21/2012.
3. NASS (2011) Crop values 2010 summary. National Agricultural Statistics Service, USDA. . Washington, D.C. 50 p. Mann Library, USDA-ESMIS website. Available: http://usda.mannlib.cornell.edu/usda/current/CropValuSu/CropValuSu-02-16-2011.pdf. Accessed: 8/11/2011.
4. NASS (2011) Crop production. National Agricultural Statistics Service, USDA. Washington, D.C. . 18 p. Mann Library, USDA-ESMIS website. Available: http://usda01.library.cornell.edu/usda/nass/CropProd//2010s/2011/CropProd-01-12-2011.pdf. Accessed: 4/21/2012.
5. NASS (2011) Vegetables. National Agricultural Statistics Service, USDA. Washington, D.C. . 13 p. Mann Library, USDA-ESMIS website. Available: http://usda01.library.cornell.edu/usda/current/Vege/Vege-10-04-2011.pdf. Accessed: 4/21/2012.

**Table S12. Statistics from 2010 for crops that require or benefit directly from bee pollination – Part I.**

| Crop | Production (1,000 tonnes) | Hectares (1,000s) | Total Value (1,000s $) | PIP[1] | VIP[2] (1,000s $) | PHB[3] | VHBP[4] (1,000s $) | POI[5] | VOIP[6] (1,000s $) |
|---|---|---|---|---|---|---|---|---|---|
| **Berries** | | | | | | | | | |
| blackberry | 20.50 | 3.04 | 33,291.00 | 0.80 | 26,632.80 | 0.90 | 23,969.52 | 0.10 | 2,663.28 |
| blueberry [cultivated] | 188.97 | 28.17 | 593,407.00 | 1.00 | 593,407.00 | 0.90 | 534,066.30 | 0.10 | 59,340.70 |
| blueberry [wild] | 37.65 | 9.31 | 50,600.00 | 1.00 | 50,600.00 | 0.90 | 45,540.00 | 0.10 | 5,060.00 |
| raspberry [black (OR)] | 0.82 | 0.53 | 2,185.00 | 0.80 | 1,748.00 | 0.90 | 1,573.20 | 0.10 | 174.80 |
| raspberry [red] | 30.03 | 4.41 | 56,426.00 | 0.80 | 45,140.80 | 0.90 | 40,626.72 | 0.10 | 4,514.08 |
| raspberry [all (CA)] | 36.74 | 2.19 | 200,288.00 | 0.80 | 160,230.40 | 0.90 | 144,207.36 | 0.10 | 16,023.04 |
| cranberry | 308.99 | 15.58 | 316,486.00 | 1.00 | 316,486.00 | 0.90 | 284,837.40 | 0.10 | 31,648.60 |
| strawberry | 1,292.78 | 23.06 | 2,245,319.00 | 0.20 | 449,063.80 | 0.10 | 44,906.38 | 0.90 | 404,157.42 |
| boysenberries | 1.00 | 0.20 | 1,834.00 | 0.80 | 1,467.20 | 0.90 | 1,320.48 | 0.10 | 146.72 |
| grapefruit | 1,114.02 | 30.84 | 285,993.00 | 0.80 | 228,794.40 | 0.90 | 205,914.96 | 0.10 | 22,879.44 |
| lemon | 782.90 | 23.07 | 380,634.00 | 0.20 | 76,126.80 | 0.10 | 7,612.68 | 0.90 | 68,514.12 |
| orange | 7,439.82 | 260.13 | 1,934,982.00 | 0.30 | 580,494.60 | 0.90 | 522,445.14 | 0.10 | 58,049.46 |
| tangelo | 37.19 | 1.90 | 6,780.00 | 0.40 | 2,712.00 | 0.90 | 2,440.80 | 0.10 | 271.20 |
| tangerine [and mandarins] | 539.77 | 18.53 | 276,135.00 | 0.50 | 138,067.50 | 0.90 | 124,260.75 | 0.10 | 13,806.75 |
| **Cucurbits** | | | | | | | | | |
| muskmelon [cantaloupe] | 854.48 | 30.24 | 314,379.00 | 0.80 | 251,503.20 | 0.90 | 226,352.88 | 0.10 | 25,150.32 |
| cucumber [fresh] | 384.74 | 17.77 | 193,643.00 | 0.90 | 174,278.70 | 0.90 | 156,850.83 | 0.10 | 17,427.87 |
| cucumber [pickled] | 498.63 | 35.69 | 184,525.00 | 0.90 | 166,072.50 | 0.90 | 149,465.25 | 0.10 | 16,607.25 |
| muskmelon [honeydew] | 145.33 | 5.95 | 49,608.00 | 0.80 | 39,686.40 | 0.90 | 35,717.76 | 0.10 | 3,968.64 |
| pumpkin | 481.90 | 19.63 | 116,539.00 | 0.90 | 104,885.10 | 0.10 | 10,488.51 | 0.90 | 94,396.59 |
| squash | 296.74 | 17.60 | 203,592.00 | 0.90 | 183,232.80 | 0.10 | 18,323.28 | 0.90 | 164,909.52 |
| watermelon | 1,866.67 | 53.66 | 492,035.00 | 0.70 | 344,424.50 | 0.90 | 309,982.05 | 0.10 | 34,442.45 |
| **Grapes** | | | | | | | | | |
| grape | 6,726.05 | 383.52 | 3,626,760.00 | 0.10 | 362,676.00 | 0.10 | 36,267.60 | 0.90 | 326,408.40 |

*Data for 2010 from NASS; all values in 1000s of 2010 USD; [1,3,5]fraction of production due to pollination by all insects, by just honey bees and by insects other than honey bees, respectively; [2,4,6]Value due to all insect pollination, just honey bees and just other insects, respectively.

**Table S13. Statistics from 2010 for crops that require or benefit directly from bee pollination – Part II.**

| Crop | Production (1,000 tonnes) | Hectares (1,000s) | Total Value (1,000s $) | PIP[1] | VIP[2] (1,000s $) | PHB[3] | VHBP[4] (1,000s $) | POI[5] | VOIP[6] (1,000s $) |
|---|---|---|---|---|---|---|---|---|---|
| **Legumes** | | | | | | | | | |
| peanut | 1,884.95 | 507.88 | 901,347.00 | 0.10 | 90,134.70 | 0.20 | 18,026.94 | 0.80 | 72,107.76 |
| soybean | 90,609.82 | 31,005.40 | 38,915,328.00 | 0.10 | 3,891,532.80 | 0.50 | 1,945,766.40 | 0.50 | 1,945,766.40 |
| **Nuts and seeds** | | | | | | | | | |
| almond | 1,282.58 | 299.47 | 2,838,500.00 | 1.00 | 2,838,500.00 | 1.00 | 2,838,500.00 | 0.00 | . |
| Macadamia nuts | 18.14 | 6.07 | 30,000.00 | 0.90 | 27,000.00 | 0.90 | 24,300.00 | 0.10 | 2,700.00 |
| canola | 1,111.73 | 579.11 | 486,865.00 | 0.50 | 243,432.50 | 0.90 | 219,089.25 | 0.10 | 24,343.25 |
| cotton [seed] | 5,616.38 | . | 1,003,861.00 | 0.20 | 200,772.20 | 0.80 | 160,617.76 | 0.20 | 40,154.44 |
| rapeseed | 1.89 | 0.89 | 975.00 | 1.00 | 975.00 | 0.90 | 877.50 | 0.10 | 97.50 |
| sunflower | 1,240.83 | 758.30 | 582,448.00 | 1.00 | 582,448.00 | 0.90 | 524,203.20 | 0.10 | 58,244.80 |
| **Tree fruits** | | | | | | | | | |
| apple | 4,219.13 | 140.00 | 2,220,817.00 | 1.00 | 2,220,817.00 | 0.90 | 1,998,735.30 | 0.10 | 222,081.70 |
| apricot | 59.33 | 4.92 | 47,486.00 | 0.70 | 33,240.20 | 0.80 | 26,592.16 | 0.20 | 6,648.04 |
| avocado | 135.44 | 24.25 | 322,108.00 | 1.00 | 322,108.00 | 0.90 | 289,897.20 | 0.10 | 32,210.80 |
| cherry [sweet] | 283.68 | 35.62 | 721,154.00 | 0.90 | 649,038.60 | 0.90 | 584,134.74 | 0.10 | 64,903.86 |
| cherry [tart] | 86.36 | 14.43 | 40,516.00 | 0.90 | 36,464.40 | 0.90 | 32,817.96 | 0.10 | 3,646.44 |
| kiwifruit | 29.66 | 1.70 | 24,961.00 | 0.90 | 22,464.90 | 0.90 | 20,218.41 | 0.10 | 2,246.49 |
| nectarine | 211.56 | 11.86 | 129,075.00 | 0.60 | 77,445.00 | 0.80 | 61,956.00 | 0.20 | 15,489.00 |
| olive | 176.90 | 13.35 | 113,360.00 | 0.10 | 11,336.00 | 0.10 | 1,133.60 | 0.90 | 10,202.40 |
| peach | 1,043.53 | 47.60 | 614,908.00 | 0.60 | 368,944.80 | 0.80 | 295,155.84 | 0.20 | 73,788.96 |
| pear | 738.09 | 23.07 | 381,695.00 | 0.70 | 267,186.50 | 0.90 | 240,467.85 | 0.10 | 26,718.65 |
| plum | 128.19 | 10.60 | 78,422.00 | 0.70 | 54,895.40 | 0.90 | 49,405.86 | 0.10 | 5,489.54 |
| prune | 343.37 | 24.69 | 149,860.00 | 0.70 | 104,902.00 | 0.90 | 94,411.80 | 0.10 | 10,490.20 |
| prune and plum | 10.98 | 1.24 | 4,915.00 | 0.70 | 3,440.50 | 0.90 | 3,096.45 | 0.10 | 344.05 |
| **TOTALS PART I + PART II** | **132,318.25** | **34,495.46** | **$61,174,042.00** | **.** | **$16,344,809.00** | **.** | **$12,356,574.07** | **.** | **$3,988,234.93** |

*Data for 2010 from NASS; all values in 1000s of 2010 USD; [1,3,5]fraction of production due to pollination by all insects, by just honey bees and by insects other than honey bees, respectively; [2,4,6]Value due to all insect pollination, just honey bees and just other insects, respectively.

**Table S14. Statistics from 2010 for crops grown from seeds that require or benefit directly from bee pollination.**

| Crop | Production (1,000 tonnes) | Hectares (1,000s) | Total Value (1,000s $) | PIP[1] | VIP[2] (1,000s $) | PHB[3] | VHBP[4] (1,000s $) | POI[5] | VOIP[6] (1,000s $) |
|---|---|---|---|---|---|---|---|---|---|
| **FIELD CROPS** | | | | | | | | | |
| alfalfa | 61,600.57 | 8,075.91 | 7,519,469.00 | 1.00 | 7,519,469.00 | 0.33 | 2,507,286.13 | 0.67 | 5,012,182.87 |
| cotton | 3,987.51 | 4,332.85 | 7,317,704.00 | 0.20 | 1,463,540.80 | 0.80 | 1,170,832.64 | 0.20 | 292,708.16 |
| **VEGETABLES** | | | | | | | | | |
| asparagus | 36.24 | 11.33 | 90,777.00 | 1.00 | 90,777.00 | 0.90 | 81,699.30 | 0.10 | 9,077.70 |
| broccoli | 826.40 | 49.25 | 648,886.00 | 1.00 | 648,886.00 | 0.90 | 583,997.40 | 0.10 | 64,888.60 |
| carrot | 1,033.15 | 27.52 | 597,362.00 | 1.00 | 597,362.00 | 0.90 | 537,625.80 | 0.10 | 59,736.20 |
| carrot | 291.23 | 5.06 | 29,608.00 | 1.00 | 29,608.00 | 0.90 | 26,647.20 | 0.10 | 2,960.80 |
| cauliflower | 284.90 | 14.71 | 247,456.00 | 1.00 | 247,456.00 | 0.90 | 222,710.40 | 0.10 | 24,745.60 |
| celery | 920.11 | 11.53 | 398,854.00 | 1.00 | 398,854.00 | 0.80 | 319,083.20 | 0.20 | 79,770.80 |
| onion | 3,320.89 | 60.57 | 1,455,103.00 | 1.00 | 1,455,103.00 | 0.90 | 1,309,592.70 | 0.10 | 145,510.30 |
| sugarbeet | 28,980.02 | 467.70 | 1,968,389.00 | 0.10 | 196,838.90 | 0.20 | 39,367.78 | 0.80 | 157,471.12 |
| **TOTALS** | **101,281.02** | **13,056.43** | **20,273,608.00** | **.** | **$12,647,894.70** | **.** | **$6,798,842.55** | **.** | **$5,849,052.15** |

*Data for 2010 from NASS; all values in 1000s of 2010 USD; [1,3,5]fraction of production due to pollination by all insects, by just honey bees and by insects other than honey bees, respectively; [2,4,6]Value due to all insect pollination, just honey bees and just other insects, respectively.
