# Supplementary material for: Insect Pollinated Crops, Insect Pollinators and US Agriculture: Trend Analysis of Aggregate Data for the Period 1992–2009
Source: PLoS One. 2012 May 22;7(5):e37235. doi: 10.1371/journal.pone.0037235 (PMC3358326; doi:10.1371/journal.pone.0037235)
Supplement: Text S4 — Decline in number of honey bee colonies and the pollinator shortage: supporting text for “Insect pollinated crops, insect pollinators and US agriculture: Trend analysis of aggregate data for the period 1992–2009.” (PDF) [file pone.0037235.s004.pdf]

Supporting Text (S4) for:
Insect pollinated crops, insect pollinators and US agriculture: Trend analysis of aggregate data for the period 1992 – 2009

Decline in number of honey bee colonies and the pollinator shortage

Nicholas W. Calderone
Department of Entomology
Cornell University
Ithaca, New York USA

This file includes:

Introduction
Materials and Methods
Results
References
Supplemental Figures S1 – S5
Supplemental Tables S15 - S16

Introduction

The trends reported in this study are likely shaped by various combinations of factors that affect the contributions from individual crops in unique ways, pollinator populations being only one such factor. Other factors include the cost of agricultural inputs (including labor, buildings, equipment and supplies); the price received for goods; the selection and use of pesticides [1,2], fertilizers [3] and growth regulators; the selection of cultivars [4]; the level of irrigation; adoption of precision agriculture [5], IPM [6] and organic methods [7-11]; adjacent land use patterns; climate trends [12-15] and changes in the design of fields and orchards so as to protect and promote wild pollinator populations. Some of these factors contribute directly to increased production, others create a more pollinator-friendly environment, and some do both. A comprehensive, multivariate analysis of the trends presented herein is not possible due to a lack of trend data specific to insect pollinated crops. However, one factor contributing to the production of each of the crops reviewed in this study is the honey bee, *Apis mellifera*. The large annual losses of honey bee colonies have raised concern over the sustainability of insect pollinated crops. This addendum examines trends in colony numbers and compares those trends to trends in crop production, cultivated area and yield for directly dependent crops. It is intended only to provide a preliminary insight into the relationship between the decline in colony numbers and the adequacy of pollination services.

Materials and Methods

Number of managed honey bee colonies and recommended number of colonies:

Data for the number of colonies in the US each year were obtained from NASS Honey - Final Estimates reports for 1986-1992, 1993-1997, 1998-2002 and 2003-2007 [16-19]; and from

NASS Annual Honey Reports for other years [20-22] (Table S15). The recommended number of colonies per hectare for pollination for each crop was obtained from published sources [23,24]. The total number of colony rentals required to meet current recommendations for each year was calculated by summing the products of the average recommended number of colonies per hectare and the total number of hectares for each crop (Table S15).

***Trends*:** I examined trends for 1) the total number of US honey bee colonies and 2) the number of colony rentals required to meet current recommendations for pollination. Data were analyzed using regression analysis (PROC AUTOREG [25] with corrections for serial autocorrelation and/or heteroscedacity of variances where required to satisfy the assumptions of the analysis) with year as the independent variable. The predicted values for production of directly dependent crops, cultivated hectares of directly dependent crops and yield of directly dependent crops were obtained from Fig. 5, Fig. 9 and Fig. 11 in the originating manuscript.

Results

Number of managed honey bee colonies in the US: The analysis included data for the period 1989 to 2010. Data for the years 1986 to 1988 are included in the figure for reference but could not be modeled; NASS did not collect data for the number of colonies for 1982 – 1985. The total number of colonies in the US (Table S15) declined from 3.53 million in 1989 (five years after detection of the tracheal mite *A. woodi* in the US [26] and two years after detection of *V. destructor* [27]) to 2.30 million in 2008, a decline of 34.81 % (Fig. S1; Table S15); however, there was an increase to 2.46 and 2.68 million colonies in 2009 and 2010, respectively (Table S16). Despite those increases, the overall trajectory maintains a downward trend. The total

number of colony rentals needed to meet current pollination recommendations (not including colonies for cotton lint) was 5.94 million in 1992 and 8.98 million in 2009 (28.18 million and 30.40 million, respectively, including colonies for cotton lint) (Fig. S2; Table S16).

Discussion

Decline in number of honey bee colonies: The number of colonies in the US declined from 5.68 million in 1947, to 3.53 million in 1989, and to 2.30 million in 2008, but increased to 2.46 million colonies in 2009 and to 2.68 million in 2010 (Fig. S1). While the overall trend remains negative, the latter figure is the highest level since 1994 when the estimate was 2.78 million colonies. The downward trend in the US managed honey bee population runs counter to global trends [28,29].

Prior to 1984, the major threats to honey bees in the US were the bacterial pathogens *Paenibacillus larvae* (formerly *Bacillus larvae*) [30-32], the causative agent of American foulbrood (AFB) and *Melissococcus pluton*, the causative agent of European foulbrood (EFB) [33]. Sulfathiazole [34] was effective against AFB [35,36] but not EFB. After the introduction of Terramycin® (oxytetracycline HCl) [34], both pathogens were well-controlled until 1998 when antibiotic resistance by AFB was confirmed [37]. The introduction of Tylan® (tylosin tartrate) [38-40] quickly restored control, and losses were relatively low and of short duration.

Significant losses of US honey bee colonies began in the mid to late 1980s when two parasitic mites, *Acarapis woodi* and *Varroa destructor* Anderson and Trueman (formerly *V. jacobsoni*) [41] were reported in 1984 and 1987, respectively [26,27]. *V. destructor* and a suite of associated

pathogens vectored by the parasite [42-48] are thought to be responsible for the greatest proportion of annual colony losses reported by beekeepers since 1987, a period characterized by frequent and often dramatic episodes of colony mortality [29,49].

Beginning with the winter of 2006/2007, several commercial migratory beekeepers began reporting high losses over the course of a year, many of uncertain etiology. Surveys of beekeepers suggested that the majority of losses were due to parasitic mites, but about 25-35% were inconsistent with that explanation [50-53]. This new condition was named Colony Collapse Disorder (CCD); however, it has not been established that all of the losses attributed to CCD are due to a single causal agent or cluster of agents. Suggested causes for CCD include beekeeper management practices, pesticides and pesticide use patterns, diminished resistance to pesticides, nutritional deficits associated with extensive monocultures and habitat alteration, climate change, exotic parasites and pathogens, diminished immunity to pathogens and interactions between two or more factors [29,54,55]. To date, no cause of CCD has been identified [56,57], but studies suggest a relationship between CCD and a variety of exotic pathogens [58-66] and possibly pesticides [67], especially the neo-nicotinoids [68-70], or a synergism between pathogens and neonicotinoids [71]; however, data are mixed [72-74].

Despite attention given to pathogens, parasites and pesticides, those factors may not bear primary responsibility for the long-term downward trend in the number of US honey bee colonies which was underway well before the arrival of parasitic mites and problems with CCD [29]. Although those factors may cause large losses each year, those losses are not directly reflected in the annual NASS estimates of the number of colonies because the beekeeping industry has proven

remarkably resilient, replacing colonies that die throughout the year in time for the next season. This resiliency may be based on economic factors, especially the price of honey and opportunities for pollination contracts. Economic opportunities may play a greater role in explaining the long-term trend than other factors [29,75]. If true, the number of colonies available for pollination should rise and fall in concert with demand for pollination contracts and other market opportunities, problems with parasites, pathogens and pesticides notwithstanding. The immediate problems for beekeepers and growers are the rise in operating costs associated with replenishing lost stock and the increase in pollination rental fees, respectively.

The pollinator shortage: Data presented here (Table S15) indicate that the available number of colonies falls short of the number required to meet current recommendations [23,24]. However, many beekeepers rent colonies more than once; so, total colony rentals are a more appropriate measure. The total number of rentals required to meet current recommendations was 30.40 million in 2010 (8.98 million w/o colonies for cotton lint). If all colonies were rented for pollination, an overly optimistic assumption, each of the 2.68 million colonies in 2010 would need to have been rented 11.34 times (3.35 times w/o colonies for cotton lint). Surveys show that commercial beekeepers rent colonies an average of 2.2 times per year [76] or 1.7 - 2.5 times per year [77].

The difference between the actual and expected number of rentals can be explained several ways: 1) crops are not being adequately pollinated; 2) crops are adequately pollinated and recommendations for honey bees are greater than required, and/or 3) native bees are contributing more than previously recognized. Although a comprehensive multi-factor analysis of trends is

not possible due to a lack of trend data specific to insect-pollinated crops, one factor common to the production of all insect pollinated crops for which such data are available is the honey bee. Data on the number of managed honey bee populations is available for the period covered by the present study (Table 1) and the contribution honey bees make to the production of each individual crop has also been estimated [78,79].

A comparison of temporal patterns for colony numbers and various crop metrics (production, cultivated area, aggregate yield and number of colonies) provides preliminary insight into the impact of the declining number of honey bee colonies on the adequacy of pollination. The cultivated area of directly dependent crops (DD Crops: e.g. apples, almonds, cherries, oranges, squash, vegetable and legume seeds, etc.) increased from 1992 through 2004, declining slightly thereafter (Fig. S3). That might suggest a response by growers to maintain production in the face of a decline in the honey bee population [80,81]; however, other data do not support that hypothesis. The production of DD Crops actually increased rapidly between 1992 and 2003, after which there was a slight downward trend (Fig. S4). The most rapid growth occurred as the number of colonies declined most rapidly. Additionally, the yield of DD Crops remained steady from 1992 through 2009 despite a declining number of colonies (Fig. S5). Those findings suggest that the decline of managed honey bee colonies has not yet resulted in a pollinator shortage. However, aggregate data mask variation among crops; and pollinator shortages may disproportionately affect crops with differing degrees of dependency on insect pollinators [80]; therefore, this conclusion should be considered tentative pending further analysis.

Determining whether or not current recommendations for the number of honey bee colonies are too high or too low will require additional studies; however, a growing body of evidence suggests that current contributions of wild, non-*Apis* pollinators are underestimated. Recently, non-*Apis* pollinators were found to be the most abundant bees across all land-use gradients for 23 farms (watermelon, muskmelon, tomato and pepper) studied in NJ and PA in 2004 and 2005 [82]; further, simulation studies suggested that non-*Apis* bees alone could provide sufficient pollination for > 90% of some 23 watermelon farms studied in NJ and PA [83]. Additional research is needed to determine the degree to which other crops in specific landscape situations may already be benefiting from wild non-*Apis* pollinators and the degree to which sustainable populations of wild non-*Apis* pollinators can be encouraged, preserved or enhanced [84,85]. Findings that non-*Apis* bees can interact synergistically with honey bees [86] suggests another interesting management strategy that merits further study.

References

1. Leng P, Zhang Z, Pan G, Zhao M, Leng PF, et al. (2011) Applications and development trends in biopesticides. African Journal of Biotechnology 10: 19864-19873.
2. Knowles A (2008) Recent developments of safer formulations of agrochemicals. Environmentalist 28: 35-44.
3. Hazarika BN, Ansari S (2007) Biofertilizers in fruit crops - a review. Agricultural Reviews 28: 69-74.
4. Devilliers SM, Hoisington DA (2011) The trends and future of biotechnology crops for insect pest control. African Journal of Biotechnology 10: 4677-4681.
5. Schimmelpfennig D, Ebel R (2011) On the doorstep of the information age: Recent adoption of precision agriculture. Economic Information Bulletin - USDA Economic Research Service.
6. Kranthi KR, Russell DA (2009) Changing trends in cotton pest management; Peshin RDAK, editor. 499-541 p.
7. Sandhu HS, Wratten SD, Cullen R (2010) The role of supporting ecosystem services in conventional and organic arable farmland. Ecol Complex 7: 302-310.
8. Sandhu HS, Wratten SD, Cullen R (2010) Organic agriculture and ecosystem services. Environmental Science & Policy 13: 1-7.
9. Drummond F, Smagula J, Annis S, Yarborough D (2009) Organic wild blueberry production. Bulletin - Maine Agricultural and Forest Experiment Station.
10. Gibson RH, Pearce S, Morris RJ, Symondson WOC, Memmott J (2007) Plant diversity and land use under organic and conventional agriculture: A whole-farm approach. J Appl Ecol 44: 792-803.
11. Gabriel D, Tscharntke T (2007) Insect pollinated plants benefit from organic farming. Agric Ecosyst Environ 118: 43-48.
12. Thomson LJ, Macfadyen S, Hoffmann AA (2010) Predicting the effects of climate change on natural enemies of agricultural pests. Biol Control 52: 296-306.
13. Luedeling E, Girvetz EH, Semenov MA, Brown PH (2011) Climate change affects winter chill for temperate fruit and nut trees. PLoS ONE 6.
14. Sutherst RW, Constable F, Finlay KJ, Harrington R, Luck J, et al. (2011) Adapting to crop pest and pathogen risks under a changing climate. Wiley Interdisciplinary Reviews-Climate Change 2: 220-237.
15. Deschenes O, Kolstad C (2011) Economic impacts of climate change on California agriculture. Clim Change 109: 365-386.
16. NASS (1995) Honey: Final estimates for 1986-1992, statistical bulletin number 912. Washington, D.C. National Agricultural Statistics Service, USDA. Mann Library, USDA-ESMIS website. http://jan.mannlib.cornell.edu/usda/nass/SB992/sb912.txt. 8/11/2011.
17. NASS (1999) Honey final estimates 1993-97, statistical bulletin number 956. Washington, D.C. National Agricultural Statistics Service, USDA. 10 p. Mann Library, USDA-ESMIS website. http://jan.mannlib.cornell.edu/usda/nass/SB992/sb956.pdf. 8/11/2011.
18. NASS (2004) Honey final estimates 1998-2002, statistical bulletin number 992. Washington, D.C. National Agricultural Statistics Service, USDA. 11 p. Mann Library, USDA-ESMIS website. http://jan.mannlib.cornell.edu/usda/nass/SB992/sb992.pdf. 8/11/2011.
19. NASS (2009) Honey final estimates 2003-2007, statistical bulletin number 1025. Washington, D.C. National Agricultural Statistics Service, USDA. 13 p. Mann Library, USDA-ESMIS website. http://jan.mannlib.cornell.edu/usda/nass/SB992/sb1025.pdf. 8/11/2011.

20. NASS (2009) Honey, hny 1 (2-09). Washington, D.C. National Agricultural Statistics Service, USDA. 6 p. Mann Library, USDA-ESMIS website. http://jan.mannlib.cornell.edu/usda/nass/Hone/2000s/2009/Hone-02-27-2009.pdf. 8/11/2011.
21. NASS (2010) Honey, hny 1 (2-10). Washington, D.C. National Agricultural Statistics Service, USDA. 6 p. Mann Library, USDA-ESMIS website. http://usda.mannlib.cornell.edu/usda/nass/Hone//2010s/2010/Hone-02-26-2010.pdf. 8/11/2011.
22. NASS (2011) Honey, hny 1 (2-11). Washington, D.C. National Agricultural Statistics Service, USDA. 6 p. Mann Library, USDA-ESMIS website. http://usda.mannlib.cornell.edu/usda/current/Hone/Hone-02-25-2011.pdf. 8/11/2011.
23. Mcgregor SE (1976) Insect pollination of cultivated crop plants: Agriculture handbook 496. . Washington, D.C.: USDA - ARS. pp. 411.
24. Delaplane KS, Mayer DF (2000) Crop pollination by bees. NY: CABI. 344 p.
25. Sas (1999) SAS/ETS user's guide, version 8. Cary, NC: SAS Institute Inc. 1,546 p.
26. Delfinado-Baker M (1984) Acarapis woodi in the United States. Am Bee J 124: 805-806.
27. Anon (1987) Varroa mites found in the USA. Am Bee J 127: 745-746.
28. Aizen MA, Harder LD (2009) The global stock of domesticated honey bees is growing slower than agricultural demand for pollination. Curr Biol 19: 915-918.
29. Vanengelsdorp D, Meixner MD (2010) A historical review of managed honey bee populations in Europe and the United States and the factors that may affect them. J Invertebr Pathol 103: S80-S95.
30. White GF (1920) American foulbrood. Washington, D.C. 46 p. website.
31. Barrett D (1955) History of american foulbrood in michigan. Bee Culture 83: 460-461.
32. Shimanuki H (1997) Bacteria. In: Morse RA, Flottum K, editors. Honey bee pests, predators, & diseases Medina, OH: A. I. Root Company. pp. 33-56.
33. Gochnauer TA. An historical review of the occurrence, diagnosis and treatment of European foulbrood disease in minnesota. Ministere de l'Agriculture, des Pecheries et de l'Alimentation. pp. 13-27.
34. Gochnauer TA (1951) Drugs fight foul brood diseases in bees. Minnesota Farm and Home Science 9: 15.
35. Haseman L, Childers LF (1944) Controlling american foulbrood with sulfa drugs / l. Haseman and l.F. Childers. Bulletin / university of missouri, college of agriculture, agricultural experiment station ; 482: Columbia, Mo. : University of Missouri, College of Agriculture, Agricultural Experiment Station, 1944.
36. Katznelson H, Jamieson CA (1952) Antibiotics and other chemotherapeutic agents in the control of bee diseases. Sci Agric [Ottawa] 32: 219-225.
37. Miyagi T, Peng C-Y-S, Chuang R-Y, Mussen E-C, Spivak M-S, et al. (2000) Verification of oxytetracycline-resistant american foulbrood pathogen paenibacillus larvae in the United States. J Invertebr Pathol 75: 95-96.
38. Hitchcock JD, Moffett JO, Lackett JJ, Elliott JR (1970) Tylosin for control of american foulbrood disease in honey bees [bacillus larvae, Apis mellifera]. J Econ Entomol 63: 204-207.
39. Elzen P-J, Westervelt D, Causey D, Ellis J, Hepburn H-R, et al. (2002) Method of application of tylosin, an antibiotic for american foulbrood control, with effects on small hive beetle (coleoptera: Nitidulidae) populations. J Econ Entomol 95: 1119-1122.
40. Pettis JS, Feldlaufer MF (2005) Efficacy of lincomycin and tylosin in controlling american foulbrood in honey bee colonies. J Apic Res 44: 106-108.

41. Anderson D-L, Trueman J-W-H (2000) Varroa jacobsoni (acari: Varroidae) is more than one species. Exp Appl Acarol 24: 165-189.
42. Bowen Walker PL, Martin SJ, Gunn A (1999) The transmission of deformed wing virus between honeybees (Apis mellifera l.) by the ectoparasitic mite varroa jacobsoni oud. J Invertebr Pathol 73 (1): 101-106.
43. Chen Y, Zhao Y, Hammond J, Hsu H-T, Evans J, et al. (2004) Multiple virus infections in the honey bee and genome divergence of honey bee viruses. J Invertebr Pathol 87: 84-93.
44. Chen YP, Pettis JS, Evans JD, Kramer M, Feldlaufer MF (2004) Transmission of kashmir bee virus by the ectoparasitic mite varroa destructor. Apidologie 35: 441-448.
45. Chen YP, Smith IB, Collins AM, Pettis JS, Feldlaufer MF (2004) Detection of deformed wing virus infection in honey bees, Apis mellifera l., in the United States. Am Bee J 144: 557-559.
46. Chen YP, Pettis JS, Collins A, Feldlaufer MF (2006) Prevalence and transmission of honeybee viruses. Appl Environ Microbiol 72: 606-611.
47. Chen YP, Siede R (2007) Honey bee viruses. In: Maramorosch KSAJMFA, editor. Advances in virus research, vol 70 70. pp. 33-80.
48. Prisco GD, Pennacchio F, Caprio E, Boncristiani HF, Jr., Evans JD, et al. (2011) Paralysis virus in the honeybee, Apis mellifera. J Gen Virol 92: 151-155.
49. Sammataro D, Gerson U, Needham G (2000) Parasitic mites of honey bees: Life history, implications, and impact. Annu Rev Entomol 45: 519-548.
50. Vanengelsdorp D, Underwood R, Caron D, Hayes J, Jr. (2007) An estimate of managed colony losses in the winter of 2006-2007: A report commissioned by the apiary inspectors of America. Am Bee J 147: 599-603.
51. Vanengelsdorp D, Hayes J, Jr., Underwood RM, Pettis J (2008) A survey of honey bee colony losses in the US, fall 2007 to spring 2008. PLoS ONE 3: Article No.: e4071.
52. Vanengelsdorp D, Hayes J, Jr., Underwood RM, Pettis JS (2010) A survey of honey bee colony losses in the United States, fall 2008 to spring 2009. J Apic Res 49: 7-14.
53. Vanengelsdorp D, Hayes J, Jr., Underwood RM, Caron D, Pettis J (2011) A survey of managed honey bee colony losses in the USA, fall 2009 to winter 2010. J Apic Res 50: 1-10.
54. Committee on the Status of Pollinators in North America NRC (2007) Status of pollinators in North America. National Academies Press. Washington, D.C. 322 p.
55. Oldroyd BP (2007) What's killing american honey bees? PLoS Biol 5: e168.
56. Vanengelsdorp D, Evans JD, Saegerman C, Mullin C, Haubruge E, et al. (2009) Colony collapse disorder: A descriptive study. PLoS ONE 4.
57. Vanengelsdorp D, Speybroeck N, Evans JD, Nguyen BK, Mullin C, et al. (2010) Weighing risk factors associated with bee colony collapse disorder by classification and regression tree analysis. J Econ Entomol 103: 1517-1523.
58. Higes M, Martcn-Hernclndez R, Botcas C, Bailcdn EG, Gonzcllez-Porto AV, et al. (2008) How natural infection by nosema ceranae causes honeybee colony collapse. Environ Microbiol 10: 2659-2669.
59. Higes M, Martin R, Meana A (2006) Nosema ceranae, a new microsporidian parasite in honeybees in Europe. J Invertebr Pathol 92: 93-95.
60. Higes M, Martin-Hernandez R, Botias C, Garrido Bailon E, Gonzalez-Porto AV, et al. (2008) How natural infection by nosema ceranae causes honeybee colony collapse. Environ Microbiol 10: 2659-2669.
61. Higes M, Martin-Hernandez R, Garcia-Palencia P, Marin P, Meana A (2009) Horizontal transmission of nosema ceranae (microsporidia) from worker honeybees to queens (Apis mellifera). Environmental Microbiology Reports 1: 495-498.

62. Higes M, Martin-Hernandez R, Garrido-Bailon E, Gonzalez-Porto AV, Garcia-Palencia P, et al. (2009) Honeybee colony collapse due to nosema ceranae in professional apiaries. Environmental Microbiology Reports 1: 110-113.
63. Higes M, Martin-Hernandez R, Martinez-Salvador A, Garrido-Bailon E, Virginia Gonzalez-Porto A, et al. (2010) A preliminary study of the epidemiological factors related to honey bee colony loss in spain. Environmental Microbiology Reports 2: 243-250.
64. Higes M, Martin-Hernandez R, Meana A (2010) Nosema ceranae in Europe: An emergent type c nosemosis. Apidologie 41: 375-392.
65. Cox-Foster DL, Conlan S, Holmes EC, Palacios G, Evans JD, et al. (2007) A metagenomic survey of microbes in honey bee colony collapse disorder. Science (Wash) 318: 283-286.
66. Bromenshenk JJ, Henderson CB, Wick CH, Stanford MF, Zulich AW, et al. (2010) Iridovirus and microsporidian linked to honey bee colony decline. PLoS ONE 5: Article No.: e13181.
67. Wu JY, Anelli CM, Sheppard WS (2011) Sub-lethal effects of pesticide residues in brood comb on worker honey bee (Apis mellifera) development and longevity. PLoS ONE: e14720.
68. Mullin CA, Frazier M, Frazier JL, Ashcraft S, Simonds R, et al. (2010) High levels of miticides and agrochemicals in north american apiaries: Implications for honey bee health. PLoS ONE 5: Article No.: e9754.
69. Johnson RM, Ellis MD, Mullin CA, Frazier M (2010) Pesticides and honey bee toxicity - USA. Apidologie 41: 312-331.
70. Cresswell JE (2011) A meta-analysis of experiments testing the effects of a neonicotinoid insecticide (imidacloprid) on honey bees. Ecotoxicology 20: 149-157.
71. Pettis JS, Vanengelsdorp D, Johnson J, Dively G (2012) Pesticide exposure in honey bees results in increased levels of the gut pathogen nosema. Die Naturwissenschaften 99: 153-158.
72. Bernal J, Garrido-Bailon E, Del Nozal MJ, Gonzalez-Porto AV, Martin-Hernandez R, et al. (2010) Overview of pesticide residues in stored pollen and their potential effect on bee colony (Apis mellifera) losses in spain. J Econ Entomol 103: 1964-1971.
73. Genersch E (2010) Honey bee pathology: Current threats to honey bees and beekeeping. Appl Microbiol Biotechnol 87: 87-97.
74. Genersch E, Von Der Ohe W, Kaatz H, Schroeder A, Otten C, et al. (2010) The german bee monitoring project: A long term study to understand periodically high winter losses of honey bee colonies. Apidologie 41: 332-352.
75. Sumner DA, H. Boriss. (2006) Beeconomics and the leap in pollination fees. University of California Giannini Foundation of Agricultural Economics: 9-11.
76. Cheung SNS (1973) The fable of the bees: An economic investigation. J Law Econ 16: 11–33.
77. Burgett M, Rucker R, Thurman W (2009) Honey bee colony mortality in the Pacific Northwest (USA) winter 2007/2008. Am Bee J 149: 573-575.
78. Robinson WS, Nowogrodzki R, Morse RA (1989) The value of honey bees as pollinators of USA crops. Part II of a two-part series. Am Bee J 129: 477-487.
79. Robinson WS, Nowogrodzki R, Morse RA (1989) The value of honey bees as pollinators of USA crops. Part I of a two-part series. Am Bee J 129: 411-423.
80. Garibaldi LA, Aizen MA, Cunningham S, Klein AM (2009) Pollinator shortage and global crop yield: Looking at the whole spectrum of pollinator dependency. Communicative & Integrative Biology 2: 37-39.
81. Southwick EE, Southwick L, Jr. (1992) Estimating the economic value of honey bees (Hymenoptera: Apidae) as agricultural pollinators in the United States. J Econ Entomol 85 (3): 621-633.

82. Winfree R, Williams NM, Gaines H, Ascher JS, Kremen C (2008) Wild bee pollinators provide the majority of crop visitation across land-use gradients in New Jersey and pennsylvania, USA. J Appl Ecol 45: 793-802.
83. Winfree R, Williams NM, Dushoff J, Kremen C (2007) Native bees provide insurance against ongoing honey bee losses. Ecol Lett 10: 1105-1113.
84. Klein A-M, Vaissiere BE, Cane JH, Steffan-Dewenter I, Cunningham SA, et al. (2007) Importance of pollinators in changing landscapes for world crops. Proc R Soc Biol Sci Ser B 274: 303-313.
85. Winfree R (2010) The conservation and restoration of wild bees. Ann N Y Acad Sci 1195: 169-197.
86. Greenleaf SS, Kremen C (2006) Wild bees enhance honey bees' pollination of hybrid sunflower. Proc Natl Acad Sci USA 103: 13890-13895.

Table S15. Number of managed honey bee colonies and total number of rentals required to meet current recommendations for pollination.

Year	Total number managed hone colonies[1]	Recommended number colony rentals[1,2]	Recommended number colony rentals[1,3]
**1989**	3.5280	**nc**	**nc**
**1990**	3.2200	**nc**	**nc**
**1991**	3.2110	**nc**	**nc**
**1992**	3.0450	28.18	5.94
**1993**	2.8750	32.06	6.49
**1994**	2.7830	34.35	7.71
**1995**	2.6550	39.80	7.79
**1996**	2.5810	32.61	6.83
**1997**	2.6310	34.48	7.67
**1998**	2.6370	30.58	9.22
**1999**	2.6520	36.04	9.19
**2000**	2.6220	35.61	9.50
**2001**	2.5500	36.98	9.33
**2002**	2.5740	33.49	8.67
**2003**	2.5990	32.25	8.24
**2004**	2.5540	33.49	7.37
**2005**	2.4090	36.34	8.74
**2006**	2.3940	33.27	7.80
**2007**	2.4430	29.29	8.31
**2008**	2.3010	23.56	8.42
**2009**	2.4620	22.83	7.78
**2010**	2.6800	30.40	8.98

[1]millions; [2]with cotton lint; [3]w/o cotton lint; nc = not calculated

Table S16. Statistics for number of managed colonies of honey bees in the US.

Variable	*y*-intercept	B_1x	B_2x^2	B_3x^3
**Millions of colonies: 1989 – 2010**				
Estimate[2] ± SE	3.4574 ± 0.0613	-0.1790 ± 0.0252	0.0116 ± 0.002964	-0.000253 ± 0.000103
t	56.40	-7.10	3.90	-2.45
$P > \|t\|$	<0.0001	<0.0001	<0.0001	<0.0145
Total R^2	0.9086	na	na	na
**Recommended number of colonies – millions[1]: 1992 - 2009**				
Estimate ± SE	6.3819 ± 0.6051	0.4295 ± 0.1362	-0.0193 ± 0.0066	na
t	10.88	3.15	-2.93	na
$P > \|t\|$	<0.0001	<0.0016	<0.0034	na
Total R^2	0.5040	na	na	na

[1]does not include colonies for cotton lint; x = year; na = not applicable; *df* = 1 all effects

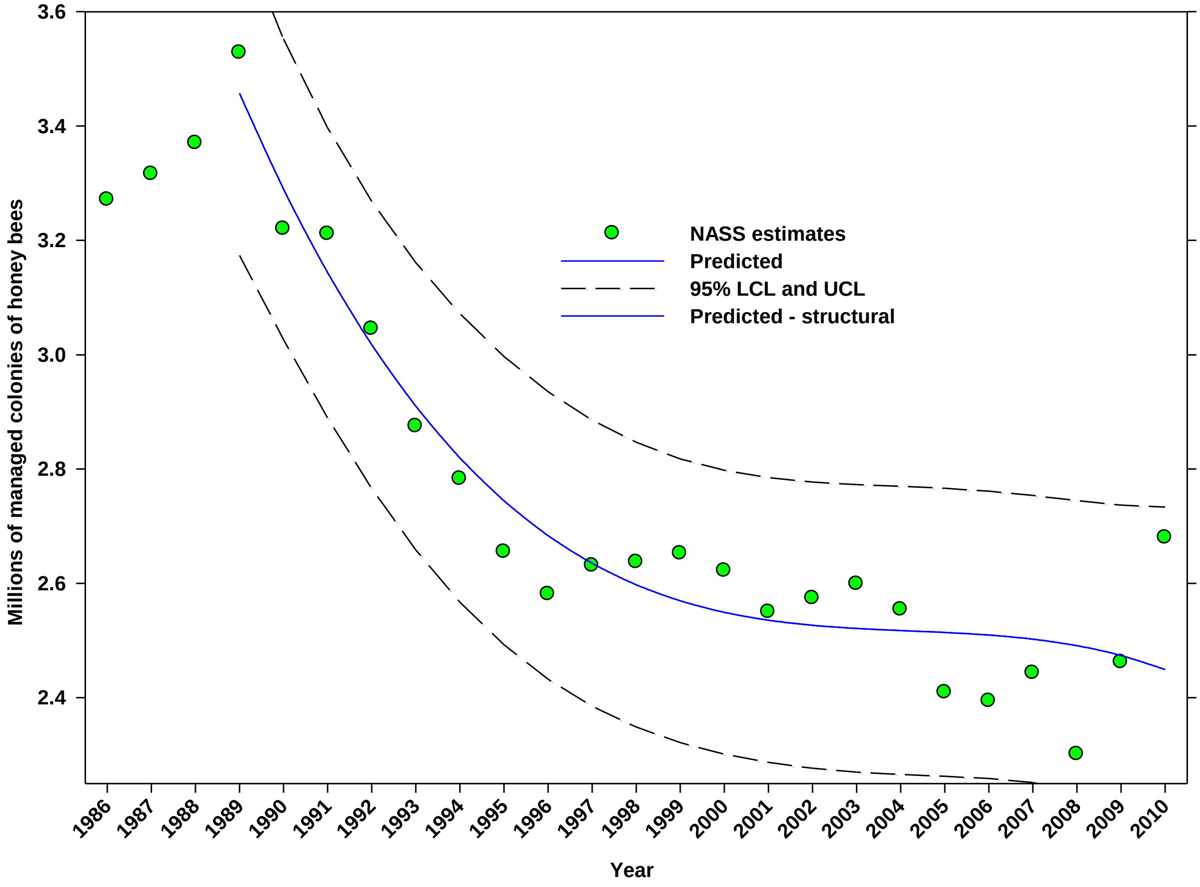

Figure S1. Number of managed colonies of honey bees in the United States. Predicted values (blue) include adjustments for serial autocorrelation and are the same as the predicted – structural values (also blue) based solely on the structural elements of the model.

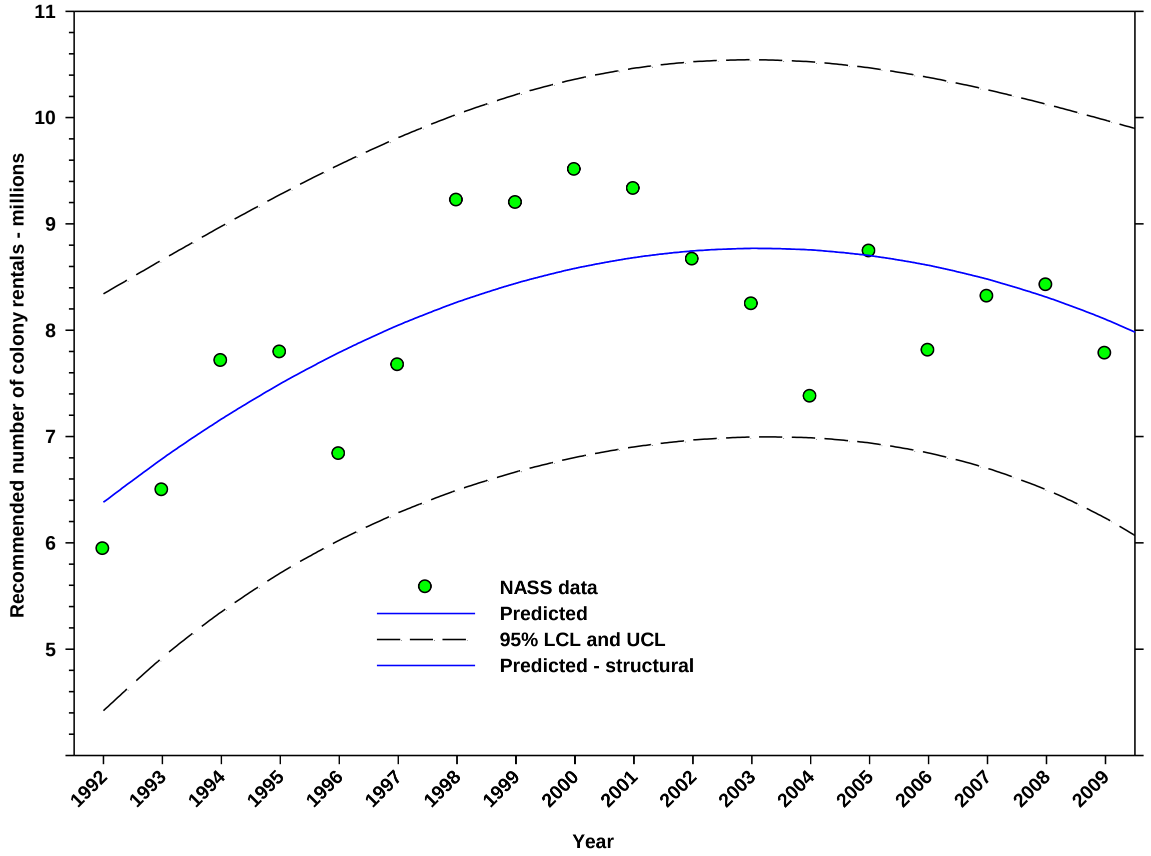

Figure S2. Number of managed colonies required to meet current recommendations for pollination. Data includes recommendations for all crops except cotton lint. Predicted values (blue) include adjustments for serial autocorrelation and are the same as the predicted – structural values (also blue) based solely on the structural elements of the model.

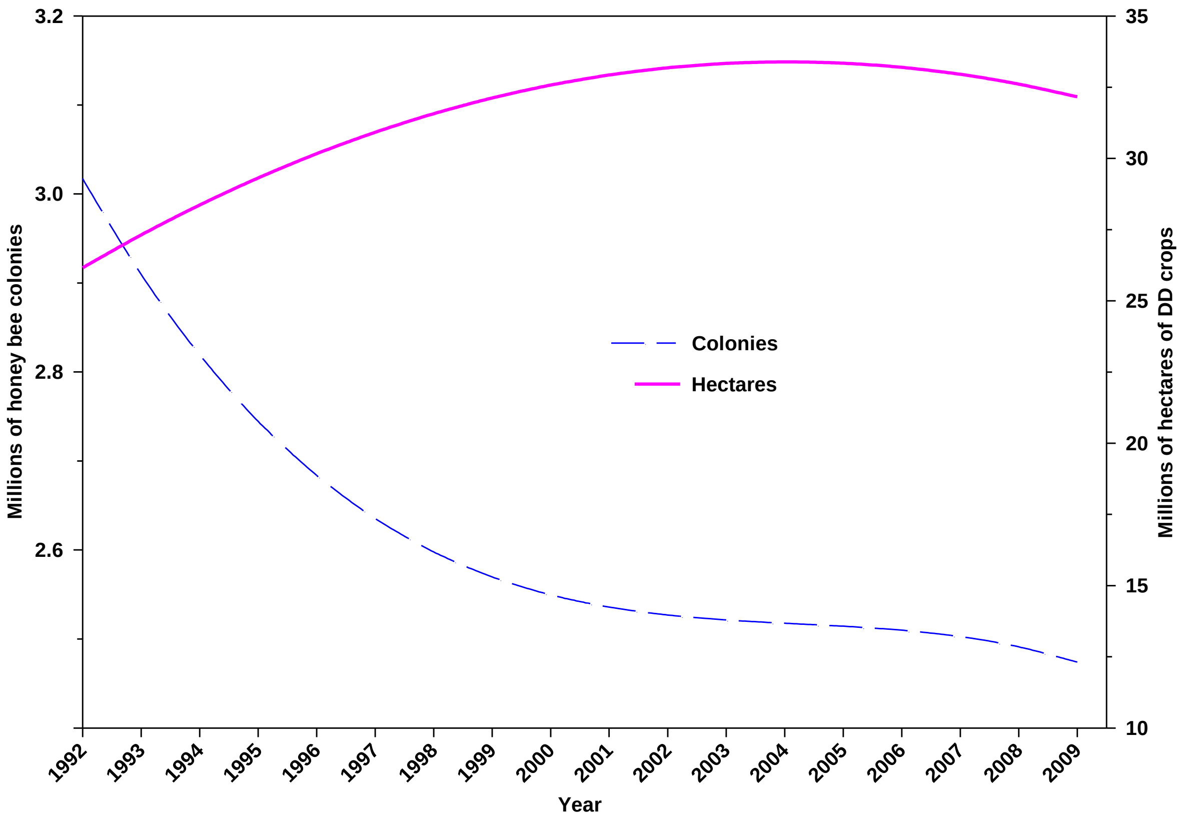

Figure S3. Predicted values for the number of managed colonies and hectares of directly dependent crops. DD = directly dependent.

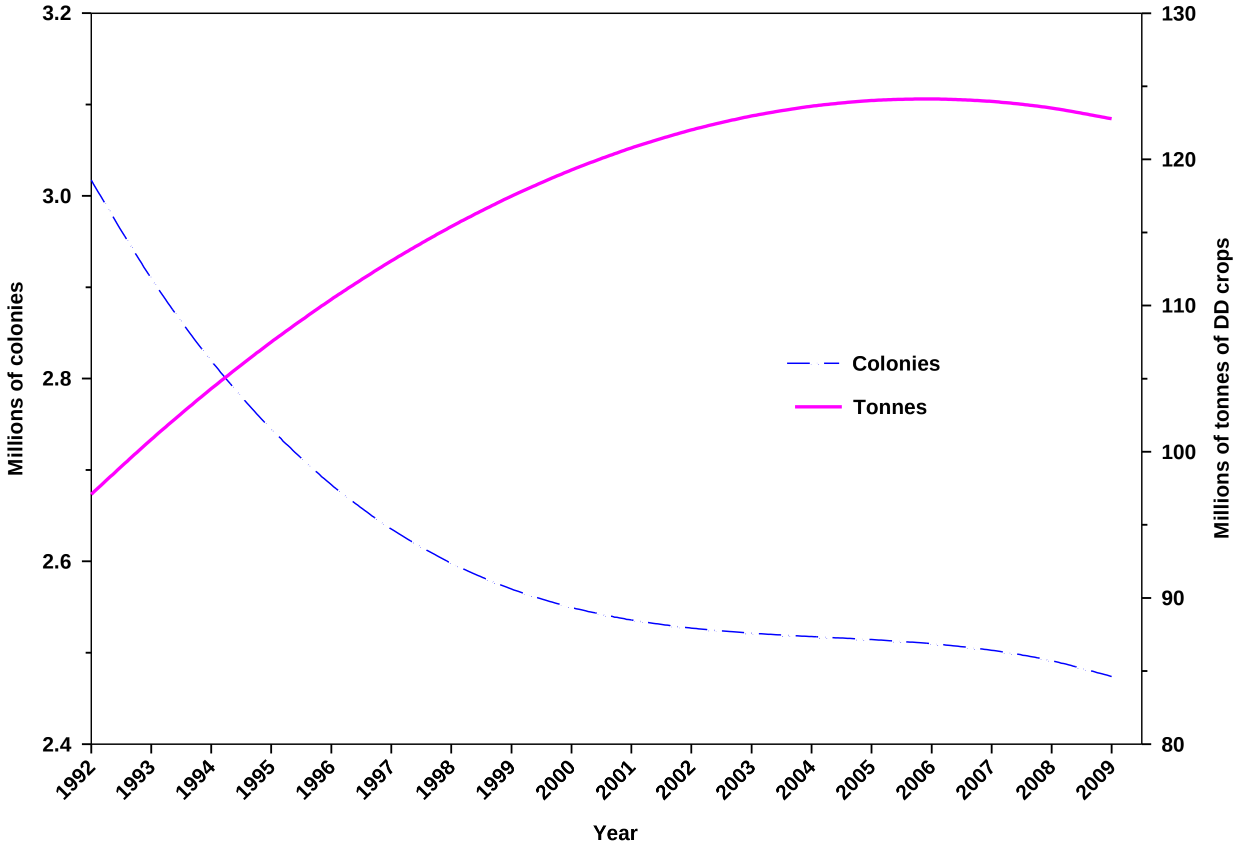

Figure S4. Predicted values for the number of managed colonies and tonnes of directly dependent crops. DD = directly dependent.

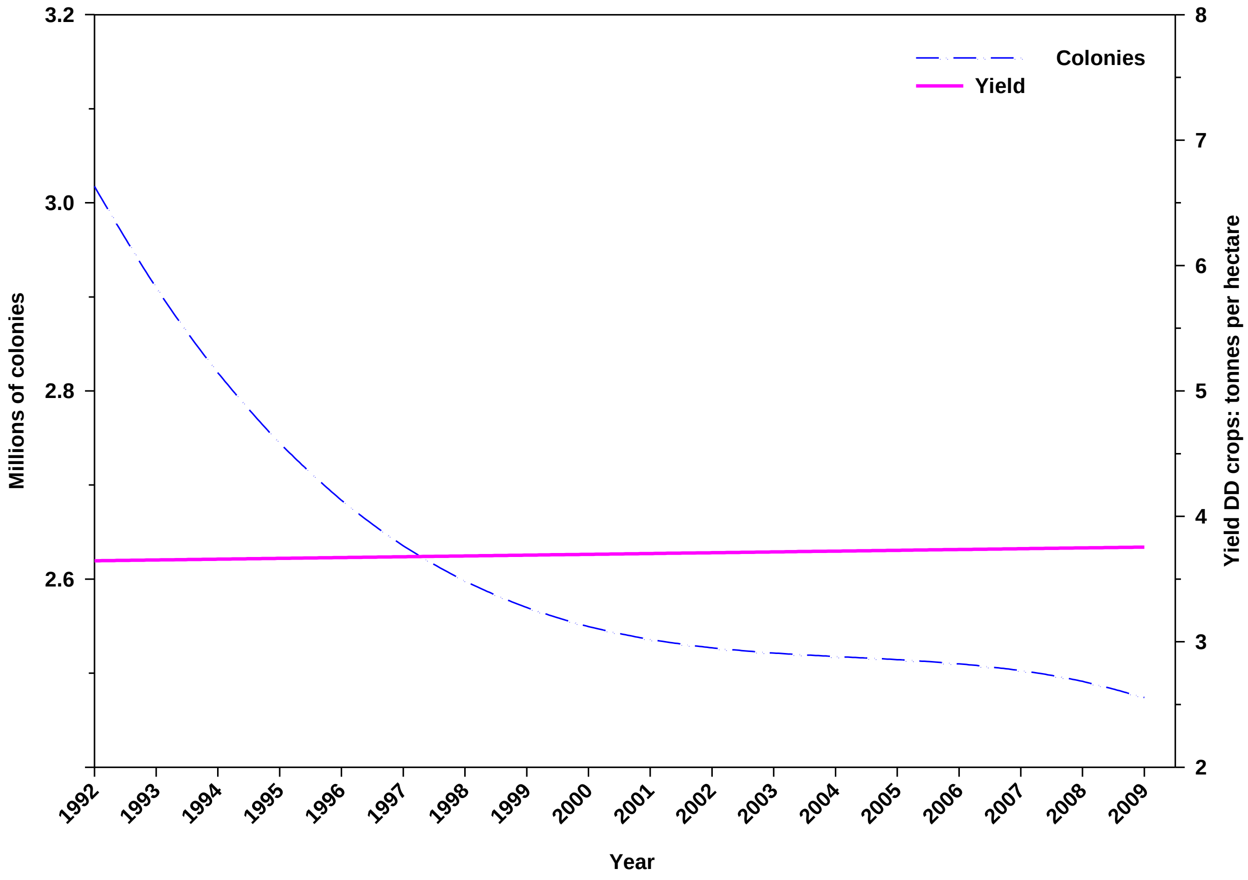

Figure S5. Predicted values for the number of managed colonies and yield of directly dependent crops. DD = directly dependent.
