# Supplementary figures and images for: Insect Pollinated Crops, Insect Pollinators and US Agriculture: Trend Analysis of Aggregate Data for the Period 1992–2009
Source: PLoS One. 2012 May 22;7(5):e37235. doi: 10.1371/journal.pone.0037235 (PMC3358326; doi:10.1371/journal.pone.0037235)

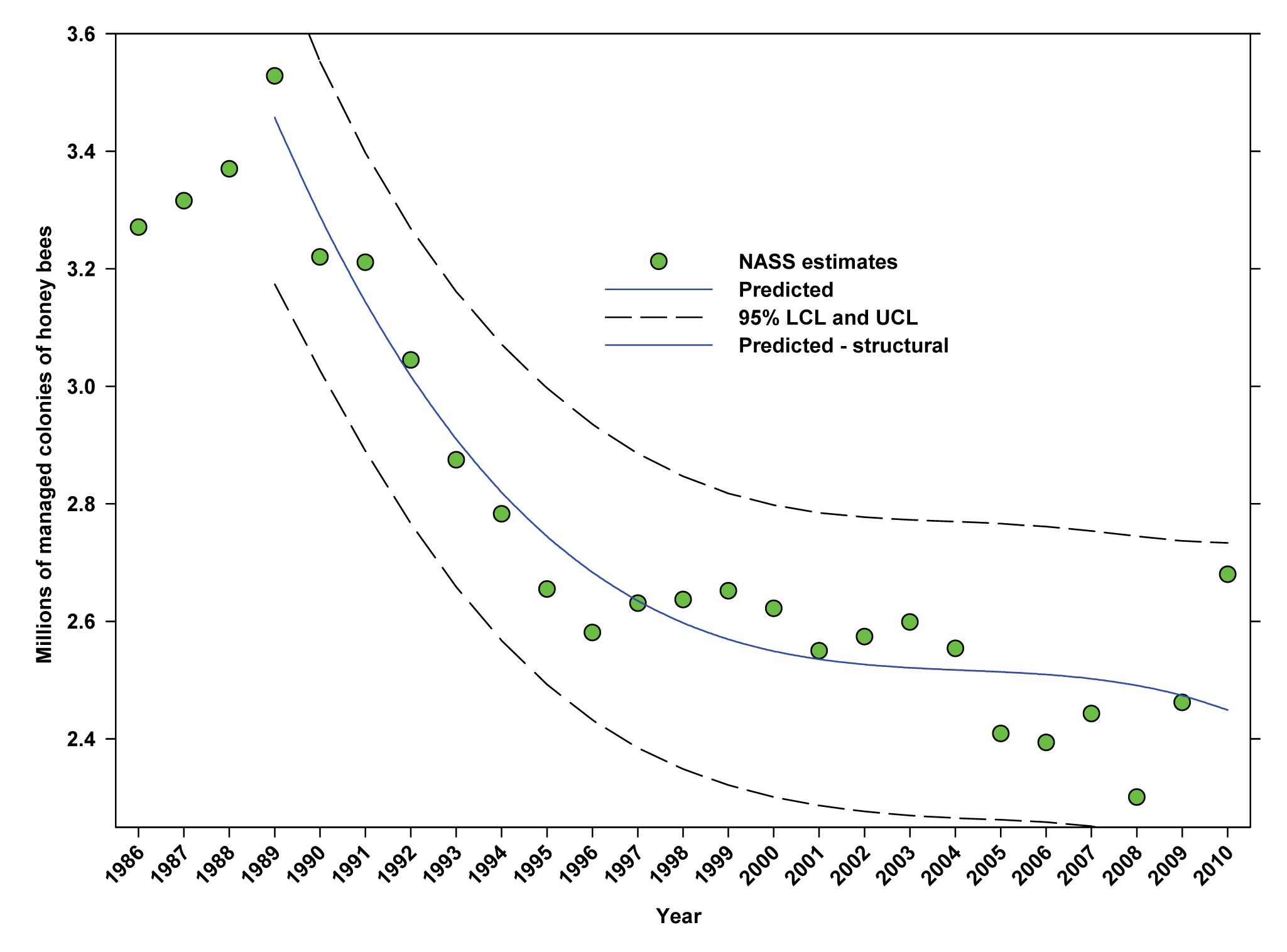

Supplement: Figure S1 — Number of managed colonies of honey bees in the United States. Predicted values (blue) include adjustments for serial autocorrelation and are the same as the predicted – structural values (also blue) based solely on the structural elements of the model. (TIF) [file pone.0037235.s005.tif]

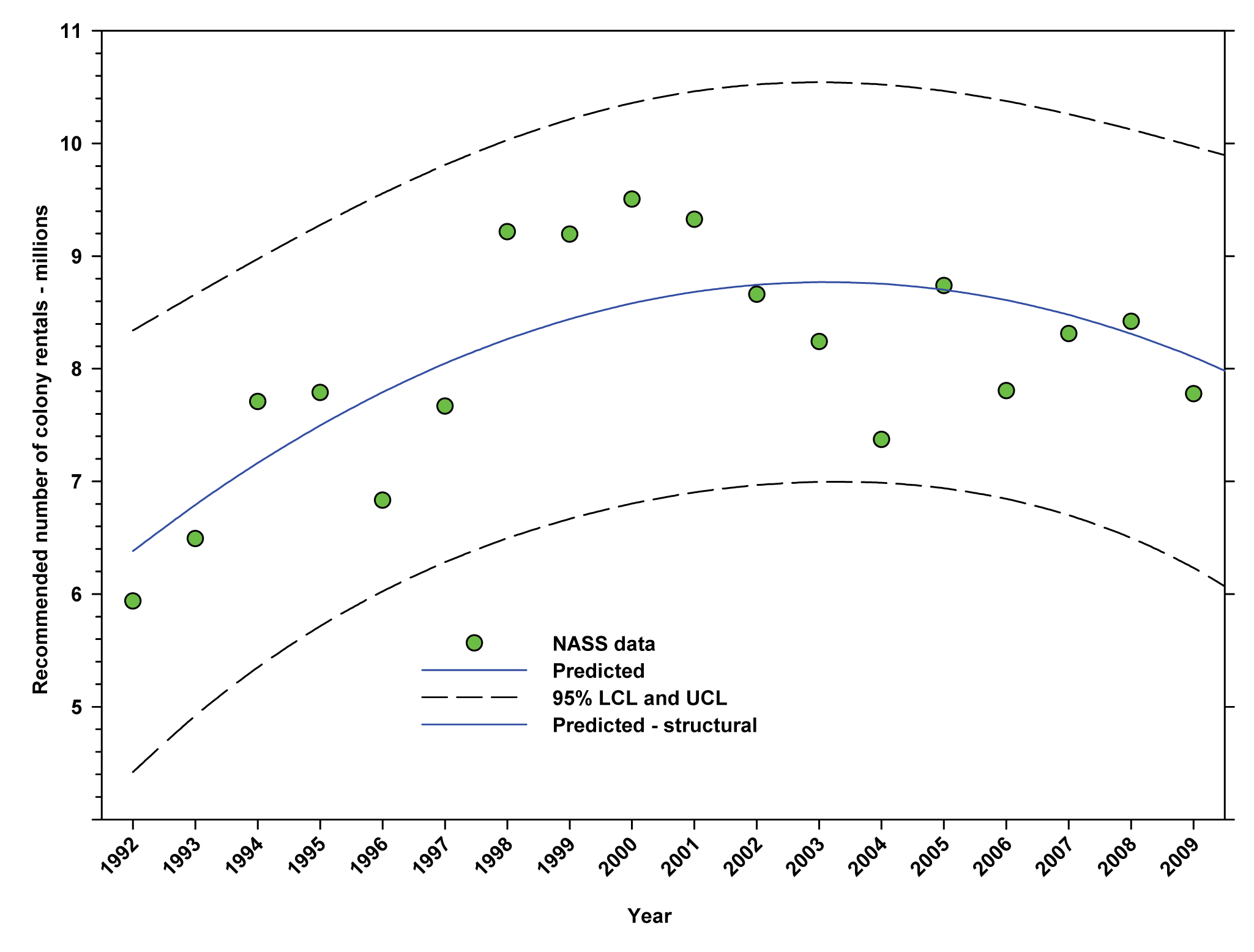

Supplement: Figure S2 — Number of managed colonies required to meet current recommendations for pollination. Data includes recommendations for all crops except cotton lint. Predicted values (blue) include adjustments for serial autocorrelation and are the same as the predicted – structural values (also blue) based solely on the structural elements of the model. (TIF) [file pone.0037235.s006.tif]

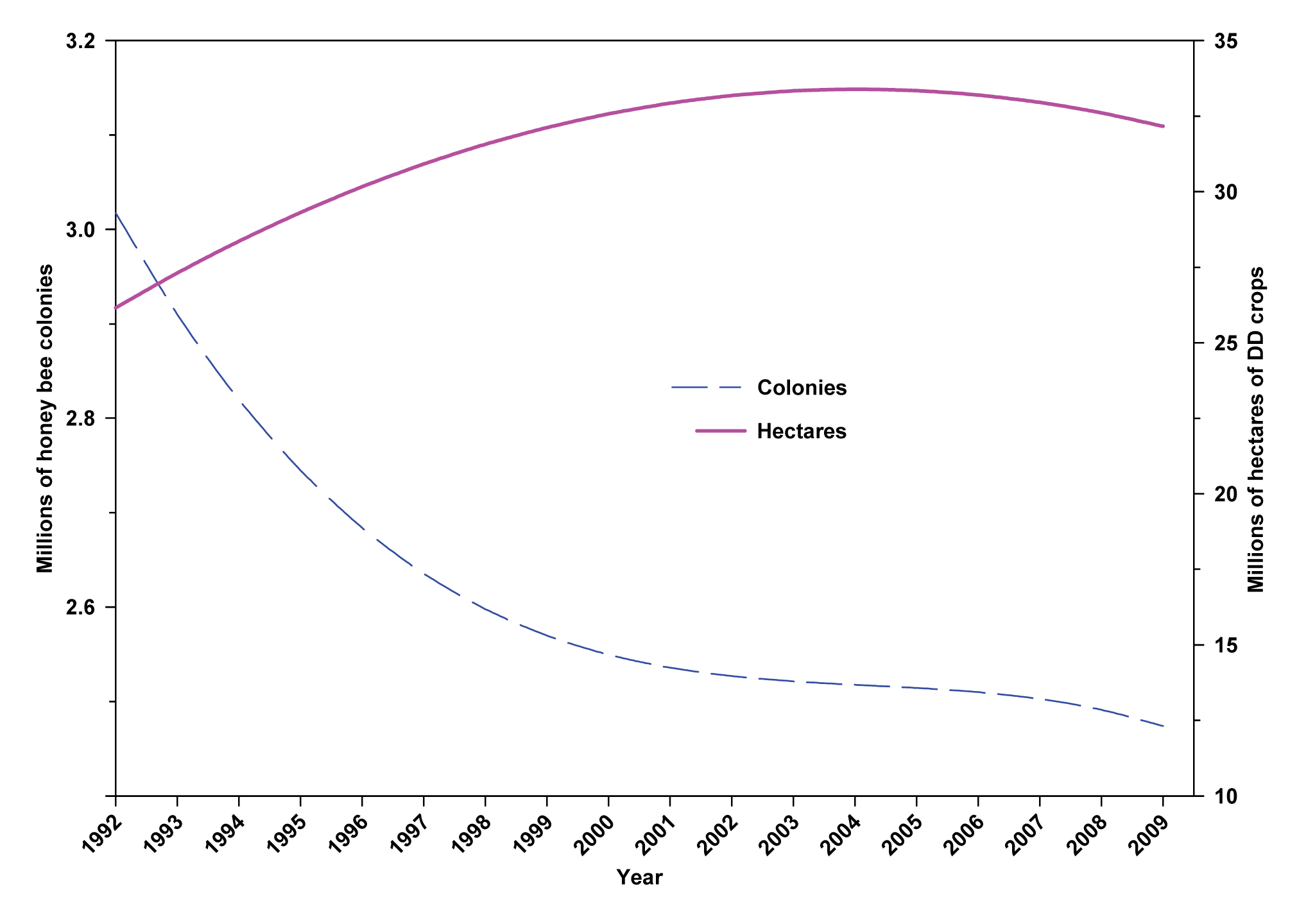

Supplement: Figure S3 — Predicted values for the number of managed colonies and hectares of directly dependent crops. DD = directly dependent. (TIF) [file pone.0037235.s007.tif]

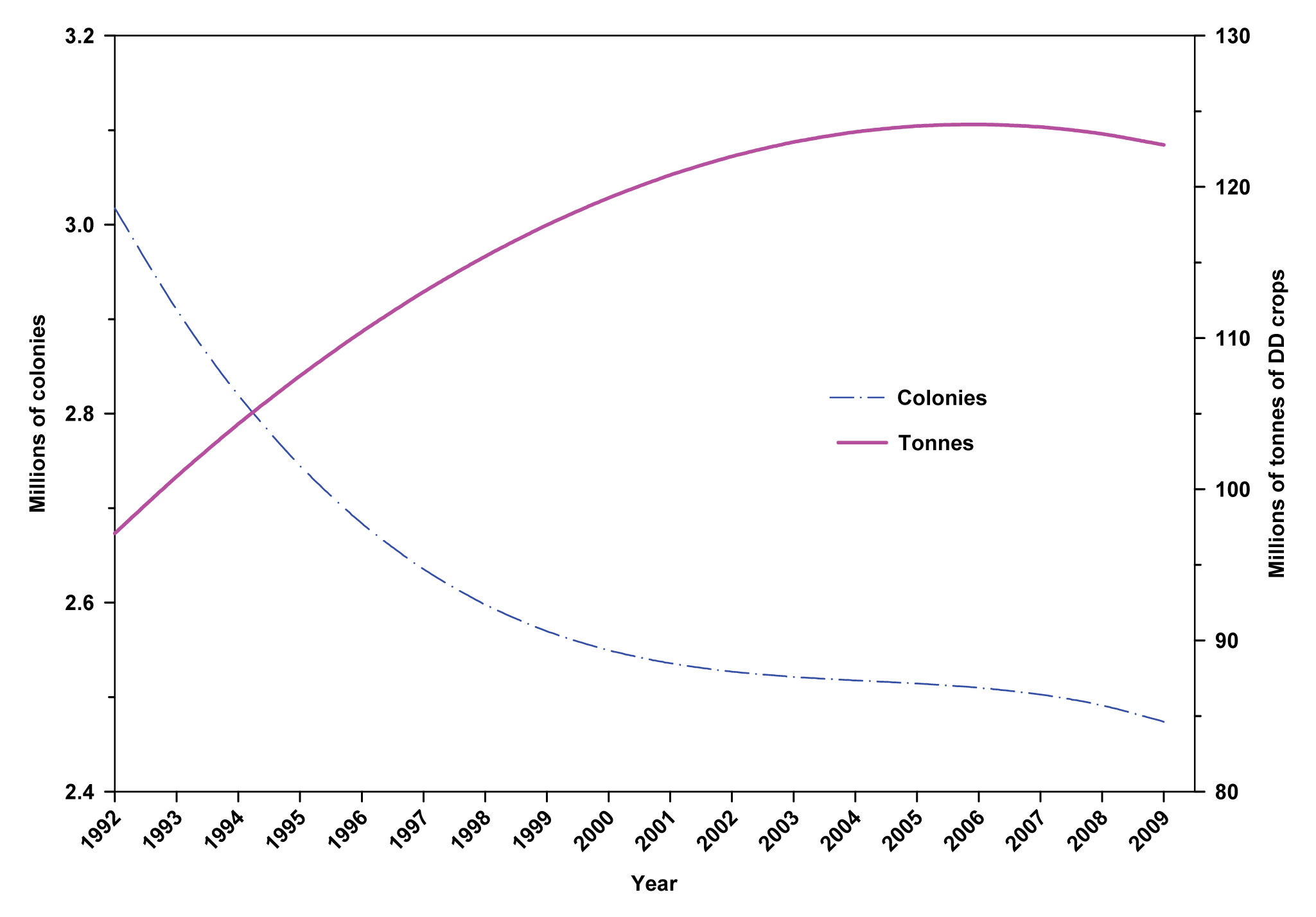

Supplement: Figure S4 — Predicted values for the number of managed colonies and tonnes of directly dependent crops. DD = directly dependent. (TIF) [file pone.0037235.s008.tif]

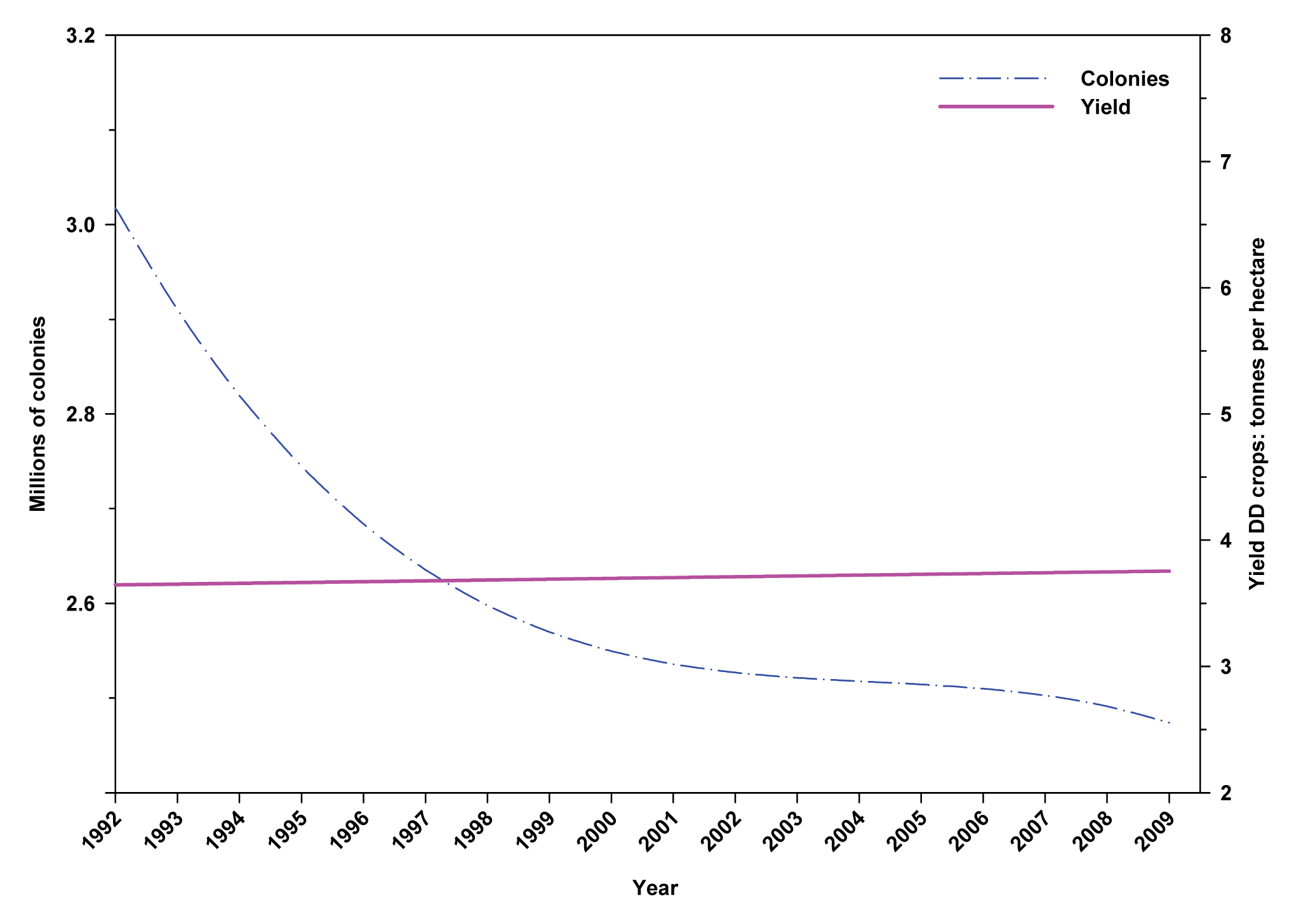

Supplement: Figure S5 — Predicted values for the number of managed colonies and yield of directly dependent crops. DD = directly dependent. (TIF) [file pone.0037235.s009.tif]
